# Surfactants promote the transport of hydrophilic compounds through hydrophobic nanopores in leaves: mechanistic insights

**DOI:** 10.1038/s41598-026-41943-z

**Published:** 2026-03-07

**Authors:** Takeshi Kobayashi, Alex Moriarty, Kristo Kotsi, Teng Dong, Ian McRobbie, Panagiota Angeli, Alberto Striolo

**Affiliations:** 1 Department of Chemical Engineering, University College London, London, WC1E 7JE United Kingdom; 2 Innospec Ltd, Ellesmere Port, Cheshire, CH65 4EY United Kingdom; 3 School of Sustainable Chemical, Biological and Materials Engineering, University of Oklahoma, Norman, OK 73019-1004 USA

**Keywords:** Epicuticular wax, Foliar spraying, Foliar uptake, Agrichemical formulations, Accelerators, Active ingredients, Biochemistry, Chemistry, Materials science

## Abstract

Conventional wisdom identifies two pathways for the uptake of active ingredients through the wax layers found on plant leaf surfaces: (1) lipophilic ingredients dissolve into the waxy substrate, and (2) hydrophilic ingredients are transported through hypothetical hydrophilic channels. Using molecular dynamics simulations, we reveal an additional mechanism by which surfactants with specific molecular structures - known as accelerators or penetrators - can enable the uptake of hydrophilic active ingredients. Depending on their structures, accelerator surfactants can penetrate nanometer-scale hydrophobic voids in the topmost wax layer, known as the epicuticular wax, promoting the formation of water nanoclusters that facilitate the uptake of hydrophilic molecules. This mechanism enables the uptake of hydrophilic nutrients such as methylglucose and certain types of electrolytes. The computational findings explain experimentally observed antagonistic effects in hard water (containing Ca$^{2+}$), which arise from selective ion binding to the waxy leaf surface. This study establishes a framework for designing next-generation agrichemical delivery systems to optimize active ingredient uptake through plant leaves by spray application.

## Introduction

Population growth, combined with the quest for safe and clean environments, places enormous demands on the agricultural sector^1,2^. More and more food is required, with smaller and smaller environmental impact. Foliar spraying (application of agrichemicals via liquid droplets sprayed on plant leaves), commonly employed in the field, is affected by several limitations^3,4^, including runoff, splashing, bounce-off, and poor rainfastness^5–12^. Of particular concern, studies suggest that less than 10% of the applied active ingredients (AIs), including nutrients and herbicides, are ultimately absorbed by the target plant cuticles, the remainder being lost^13–15^. To enhance uptake, surface-active chemicals, such as accelerator surfactants, are incorporated into commercial agrichemical formulations. Due to their function, accelerators are also referred to as penetrators. Even though these surfactants promote AI uptake, as documented at the field scale, particularly in the context of lipophilic AIs^16–27^, a detailed molecular-level understanding of the mechanisms of action of accelerators is currently lacking.

**Fig. 1:**
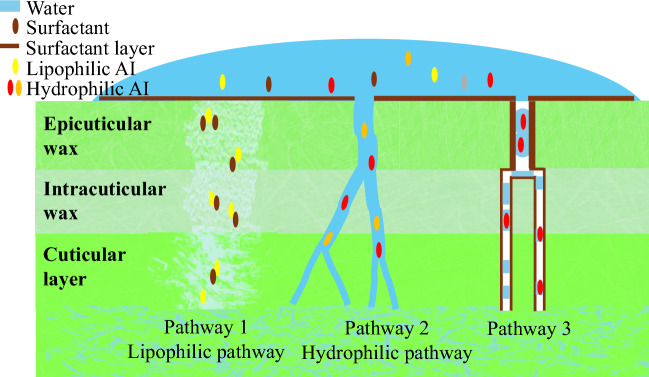
A schematic illustration of the three distinct AI uptake pathways considered in this work, with pathway 3 being the one proposed based on simulation results. Pathway 1: Lipophilic AIs dissolve into the amorphous wax and diffuse together with surfactants. Pathway 2: Hydrophilic AIs diffuse through hydrophilic aqueous pores formed under certain humidity conditions. Pathway 3: Surfactants penetrate hydrophobic nanopores together with water and certain types of hydrophilic AIs.

According to conventional wisdom, lipophilic and hydrophilic AIs are absorbed within the plant cuticle (top layer of a leaf) via two distinct transport pathways^4,21,28–30^. The uptake of lipophilic AIs is explained by the solution–diffusion pathway^21,31^. According to this mechanism, the chemical compounds are initially solvated into the wax layer found on the leaf, and then diffuse to the underlying cells. This process is controlled by two key parameters: the wax–water partition coefficient, which describes the solubility of the compound in the wax layers, and the diffusion constant, which governs the rate of movement through the wax. Because hydrophilic AIs are little soluble in the wax layer, their foliar uptake pathway must be different. According to one hypothesis, hydrophilic AIs diffuse through aqueous nanopores embedded within the wax layers^32–34^. Unfortunately, it has been challenging to confirm this mechanism^32–34^ because the pore radius should range from 0.3 to 2.4 nm, depending on the plant species^33^. Further, experiments show that uptake occurs when humidity levels exceed the AIs deliquescence point^32,35,36^, which complicates direct observation. Hence, additional pathways have been suggested for the foliar uptake of hydrophilic AIs. This manuscript explores the possibility that certain types of surfactants, known as accelerators, promote a third uptake mechanism. In Fig. 1, the mechanism explored here (pathway 3) is schematically compared to pathway 1 (lipophilic pathway) and pathway 2 (hydrophilic pathway).

**Fig. 2:**
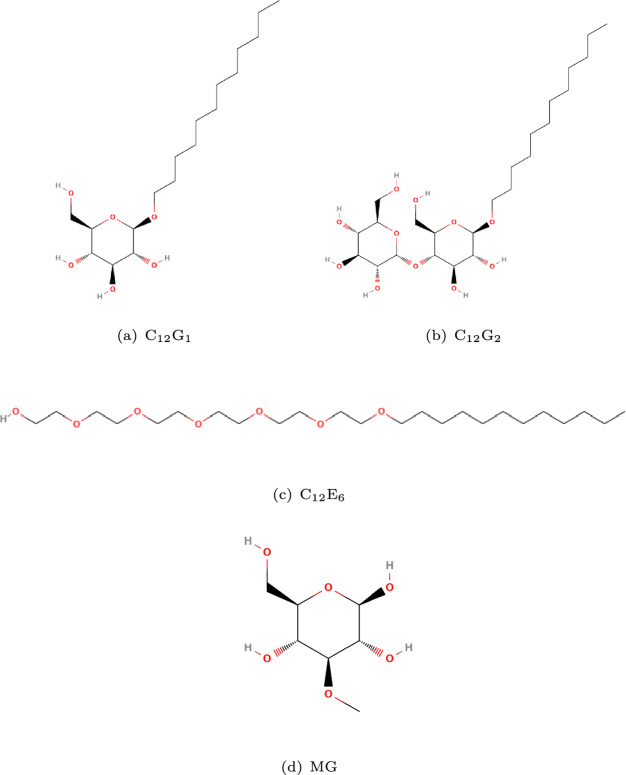
Chemical structures of non-ionic surfactants (**a**) n-dodecyl $\beta$-D-glucopyranoside (C$_{12}$G$_{1}$), (**b**) n-dodecyl $\beta$-D-maltoside (C$_{12}$G$_{2}$), (**c**) hexaethylene glycol monododecyl ether (C$_{12}$E$_{6}$), and non-ionic hydrophilic AI (**d**) 3-O-methyl-$\beta$-*D*-glucopyranose (MG).

Two emergent classes of nonionic surfactants used to increase hydrophilic AIs uptake are alcohol ethoxylates (AE), abbreviated as C$_\textrm{x}$E$_\textrm{y}$ or C$_\textrm{x}$EO$_\textrm{y}$ (x: number of carbon atoms in the hydrophobic alkyl tail, y: number of ethylene oxide units (EO) in the hydrophilic head group), and the sugar-based alkyl polyglycosides (APGs), abbreviated as C$_\textrm{x}$G$_\textrm{y}$ (x: number of carbon atoms in the hydrophobic alkyl tail, y: number of glucoside units in the hydrophilic head group)^37^. The chemical structure of AEs and APGs are shown in Fig. 2. Both AEs and APGs can reduce surface and interfacial tension, but only AEs can effectively plasticize the wax layer^38–42^. When AEs are added to formulated agrichemical products, the hydrophobic AI uptake rate can increase up to 100-fold^18,20,24–26,43–45^ because AEs can increase the diffusion rate of lipophilic AIs through wax^19–22,25–28,44,46,47,48,49,50,51^. However, AEs also increase the uptake of hydrophilic nutrients, such as methylglucose (MG). In contrast, other accelerator surfactants, such as diethylsuberate (DESU) and tributyl phosphate (TBP), do not enhance the uptake of hydrophilic AIs; rather, they manifest antagonistic effects^52^. On the other hand, APGs enhance the uptake of both water and ionic compounds^32,36,53,54^, but not that of hydrophobic AIs. Presumably, this is because APGs help the aqueous system fill partially hydrophilic pores^55^.

Experimental observations are difficult to generalize if only two mechanisms are responsible for foliar AI uptake. For example, MG can achieve higher uptake than CaCl$_2$, although both should diffuse presumably only through Pathway 2. This suggests that MG may engage transport pathways not accessible to CaCl$_2$^52^. Further, while MG does not prevent AE penetration, other lipophilic AIs do, suggesting that AEs might traverse the amorphous wax layer together with lipophilic solutes, excluding the lipophilic pathway for MG^18^. Synergy among different surfactants is possible, as described in Ref^56^, which would be easier to explain in the presence of additional uptake mechanisms. Antagonistic effects could also be explained more easily if multiple uptake pathways were present. For example, it has been reported that hard water can compromise AEs adsorption^57,58^, and a correlation has been reported between the slow penetration of AEs and the slow uptake of MG in the presence of CaCl$_{2}$^57^. If the role of AEs was only relevant for the uptake of hydrophilic AIs through Pathway 2, this antagonistic effect would not occur. On the other hand, Ca$^{2+}$ ions could affect many interfacial properties, and also strongly interact with charged species, leading, e.g, to the formation of supra-molecular complexes, whose large molecular size would hinder penetration through Pathway 2^36,59–61^.

Explaining these experimental observations requires one additional uptake pathway, accessible only to specific combinations of surfactants/hydrophilic AIs. It is hypothesized here that AEs enable this pathway for MG, and that electrolytes such as CaCl$_2$ prevent the activation of this pathway. As discussed by others^52^, the mechanism hypothesized here might account for $\sim$ 40% of the total MG uptake.

To rationalize the observations above, it helps considering that the plant cuticle consists of multiple layers. To reach the underlying cells, any AIs on the leaf surface must first traverse the topmost epicuticular wax layer, which extends several hundreds of nanometers^62–64^ and serves as initial barrier^22,65,66^. The removal of this wax layer increases solute uptake 10- to 1000-fold for water or organic solutes^34^, and three-fold for ionic solutes^54^. These differences may reflect the distinct pathways utilized by nonionic and ionic solutes, where the crystalline regions of the wax predominantly influence tortuosity, while the amorphous regions govern the diffusion and solubility of lipophilic AIs^19^. Once a solute penetrates the epicuticular wax layer, its subsequent transport may occur either through the wax matrix or via aqueous pores, depending on the solute’s hydrophilicity or lipophilicity. Of note, once the epicuticular wax is removed, the remaining cuticle exhibits permanent aqueous pores^54,67^.

The epicuticular wax comprises hydrocarbons with various functional groups, including alkanes and carboxyl groups (-COOH), which dissociate into the deprotonated form at ambient conditions (pH = 7)^67^. The isoelectric point of the plant cuticle is $\sim$ 3^68^, indicating that the surface is negatively charged when in contact with aqueous systems. Experimental results^69,70^ report binding of Ca$^{2+}$, Rb$^{+}$, Cl$^{-}$, and urea to the cuticular surface. A significantly higher adsorption of Ca$^{2+}$ (2.73 $\times$ 10$^{-9}$ mol/cm$^{2}$) was observed compared to Rb$^{+}$ (0.018 $\times$ 10$^{-9}$ mol/cm$^{2}$) on the outer surface of the leaf. Strong ion binding to other plant surfaces was also observed, in the order H$^{+}$ > Ba$^{2+}$ > Ca$^{2+}$ > Mg$^{2+}$ > Rb$^{+}$ > NH$_{4}^{+}$ > K$^{+}$ > Na$^{+}$ > Li$^{+}$^71^. MD simulations, ab initio simulations, and Raman spectroscopy explain, in part, these trends^72^. Mathematical modeling also suggests the importance of ion binding on the surface^73–75^.

Molecular dynamics (MD) simulations are implemented to elucidate the mechanisms of action of AEs and APGs for hydrophilic AI uptake through hydrophobic pores carved out of wax layers meant to model epicuticular layers, and to probe the antagonistic effect due to Ca$^{2+}$. The level of detail implemented in MD simulations does not allow us to explicitly consider effects due to surface roughness, and stomatal opening on uptake^32,33^. However, MD simulations allow us to investigate the impact of atomic-level features of surfactants, AIs, and wax molecules. The nonionic surfactant hexaethylene glycol monododecyl ether (C$_{12}$E$_{6}$) is chosen as representative of the AE family, while n-Dodecyl $\beta$-D-glucopyranoside (C$_{12}$G$_{1}$) is chosen to represent the APG family. Although in practical formulations surfactants are present as mixtures^41^, studying single surfactants allows us to conduct a comparative analysis. C$_{12}$E$_{6}$ and C$_{12}$G$_{1}$ have identical hydrophobic alkyl tail group, but differ in their hydrophilic head groups. To connect with available experiments, MG was chosen as the nonionic hydrophilic AI. The molecular structures used in this study are shown in Fig. 2. Building on the experimental observations summarized above, the epicuticular wax layer is modeled in our MD simulations with the following atomistic details:

1. The crystalline wax structure reproduces that suggested by X-ray diffraction data.
2. The target area is a 6 nm $\times$ 6 nm region containing one nanoscale pore.
3. Surfactants and AIs are present at very high concentration but not crystallized, representing the final stages of droplet evaporation, when the hydrophilic AIs penetration begins.
4. To model surface charge density, selected CH$_3$ groups on the wax surface or within the pores were replaced by COO$^-$/COOH groups.

**Fig. 3:**
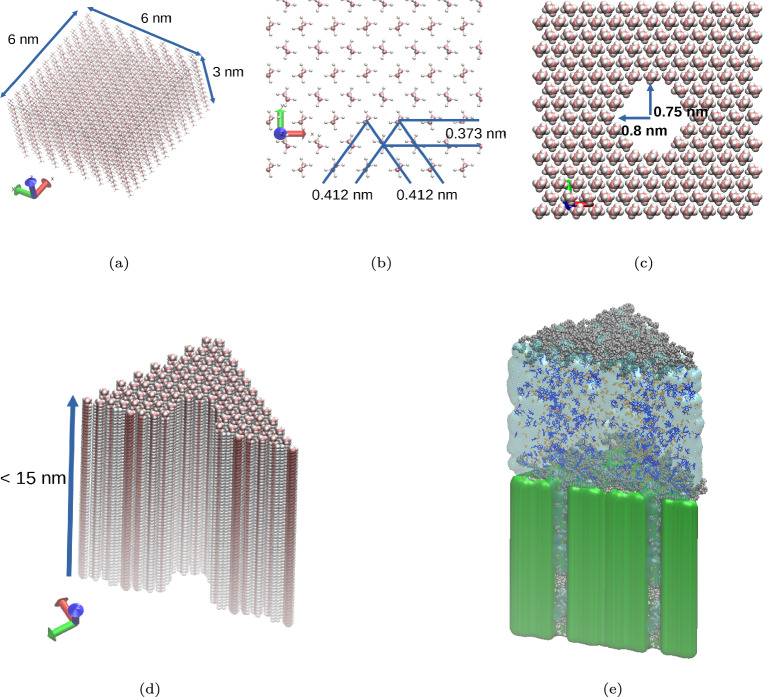
(**a**) and (**b**): The orthorhombic symmetric crystalline structure of the model plant epicuticular wax, as developed based on experimental X-ray diffraction results^[84,85]^. (**a**) the overall view of the unit cell, and (**b**) a close-up view of the top surface of the model epicuticular wax layer. (**c**) and (**d**): Simulation snapshots of the model leaf with a nanometer-scale pore. (**c**) the top view of a unit cell where 14 alkane molecules are removed to create a pore. (**d**) the corresponding cross-sectional view. The molecular representations in (**c**) and (**d**) differ from those in (**a**) and (**b**) to enhance the visibility of the pore structure. (**e**): A representative cross-sectional snapshot for an aqueous solution containing MG and C$_{12}$E$_{6}$ placed on top of the model leaf containing three pores. The color scheme is as follows: blue for MG, gray for C$_{12}$E$_{6}$, transparent for water, and green for the epicuticular wax, which includes three pores across three unit cells.

More simulation details are provided in Section “Methods” - ("The model epicuticular wax on plant leaves", "Nano-scale pore within the epicuticular wax", and "Surfactants and active ingredients on the leaf wax"), and shown schematically in Fig. 3.

## Results

The properties of AEs surfactants, potential accelerators, are compared to those of APGs, wetting agents. Because the focus is on hydrophilic AIs, Pathway 1 (see Fig. 1) is not relevant. Uptake via the hypothetical Pathway 2 is expected to be governed by surface/interfacial tension. Although this pathway is not considered further here, simulation results for quantities such as surface/interfacial tension are shown in the Methods section (Surfactants and active ingredients on the leaf wax) and Supplementary Information (SI Section C.2).

### Surfactant adsorption on the model leaf wax

**Fig. 4:**
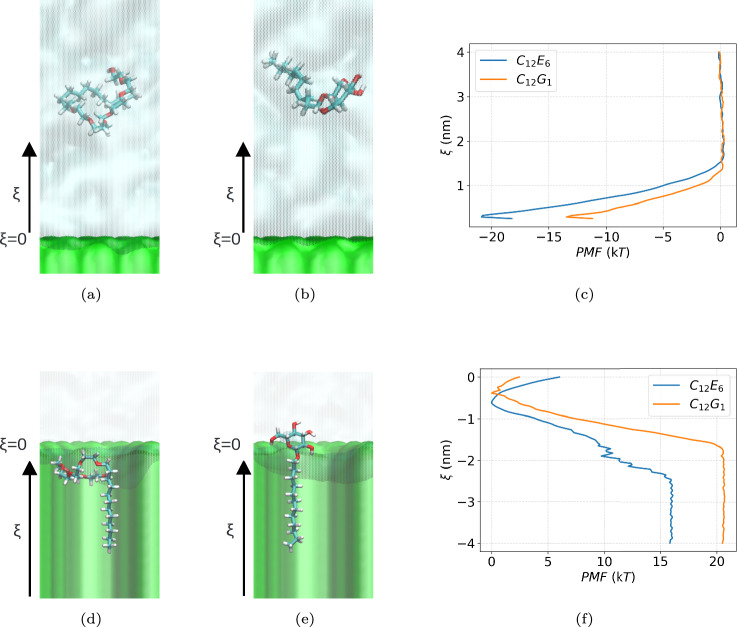
(**a**)(**b**)(**c**): Schematic illustrations of the calculation of adsorption free energy $\Delta G^\textrm{adsorption}$ on the leaf wax. Snapshots of the system containing a single surfactant molecule of (**a**) C$_{12}$E$_{6}$ and (b) C$_{12}$G$_{1}$. (**c**) The calculated potential of mean force (PMF) for (blue) C$_{12}$E$_{6}$ and (orange) C$_{12}$G$_{1}$. (**d**)(**e**)(**f**): Schematic illustration of the calculation of the single-surfactant penetration free energy barriers $\Delta G^{\textrm{penetration1}}$ into nanopore in the wax. Snapshots of the system containing a single surfactant molecule of (**d**) C$_{12}$E$_{6}$ and (e) C$_{12}$G$_{1}$ penetrating into the pore. (**f**) The calculated PMFs for the penetration for (blue) C$_{12}$E$_{6}$ and (orange) C$_{12}$G$_{1}$. In both cases, the reaction coordinate $\xi$ is defined as the center-of-mass coordinate of the surfactant molecule along the surface-normal (vertical) direction ($\xi$ = 0 nm at the surface).

To quantify the propensity of AEs and APGs to adsorb on the leaf wax, adsorption free energy profiles, referred to as potentials of mean force (PMF), are calculated for C$_{12}$E$_{6}$ and C$_{12}$G$_{1}$ as they transfer from the aqueous system to the wax surface. The PMF profiles are expressed as a function of $\xi$, the center-of-mass position of the surfactant perpendicular to the wax surface. The algorithms used for the calculations and for estimating errors are summarized in the Methods (Section Adsorption/penetration free energy calculations) and SI (Section D.2). The computed PMFs are shown in the top panels of Fig. 4. It can be seen that the free energy decreases for both surfactants as they move from the aqueous phase to the wax surface, where the free energy is at a minimum. This indicates spontaneous adsorption, expected because the wax surface is hydrophobic, and both surfactants are surface active. However, the results differ for the two surfactants. To quantify the differences, the adsorption free energy $\Delta G^\textrm{ads}$ is defined as $\mathrm{PMF(\xi _{surface}=3.0~nm)} - \mathrm{PMF(\xi _{bulk} = 0.25~nm)}$, which yields $\Delta G^\textrm{ads}_{{\textrm{C}}_{12}{\textrm{E}}_{6}} \approx -14~kT$ and $\Delta G^\textrm{ads}_{\textrm{C}_{12}{\textrm{G}}_{1}} \approx -21~kT$. These results reflect the more hydrophilic nature of the C$_{12}$G$_{1}$ headgroup compared to C$_{12}$E$_{6}$.

PMF profiles were also computed as the surfactants move from the entrance facing the aqueous systems to the interior of the nanoscale pores, as depicted in the bottom panels of Fig. 4. In this case, the free energy goes through a local minimum, indicating that the surfactants favor having their tailgroups in the hydrophobic pores, but then the free energy increases as the surfactants further penetrate the pores, indicating that the process is not thermodynamically spontaneous. The penetration free energy barrier, defined as $\mathrm{PMF(\xi _{entrace})} - \mathrm{PMF(\xi _{inside} = -3.0~nm)}$ ($\xi _\textrm{entrace}$ = 0.7 nm for C$_{12}$E$_{6}$, and 0.4 nm for C$_{12}$G$_{1}$), is found to be $\Delta G^{{\textrm{penetration1}}}_{\textrm{C}_{12}{\textrm{E}}_{6}} \approx 16~kT$ and $\Delta G^{{\textrm{penetration1}}}_{\textrm{C}_{12}{\textrm{G}}_{1}} \approx 20~kT$. This difference is consistent with the more hydrophilic nature of the headgroup of C$_{12}$G$_{1}$ compared to that of the other surfactant.

**Fig. 5:**
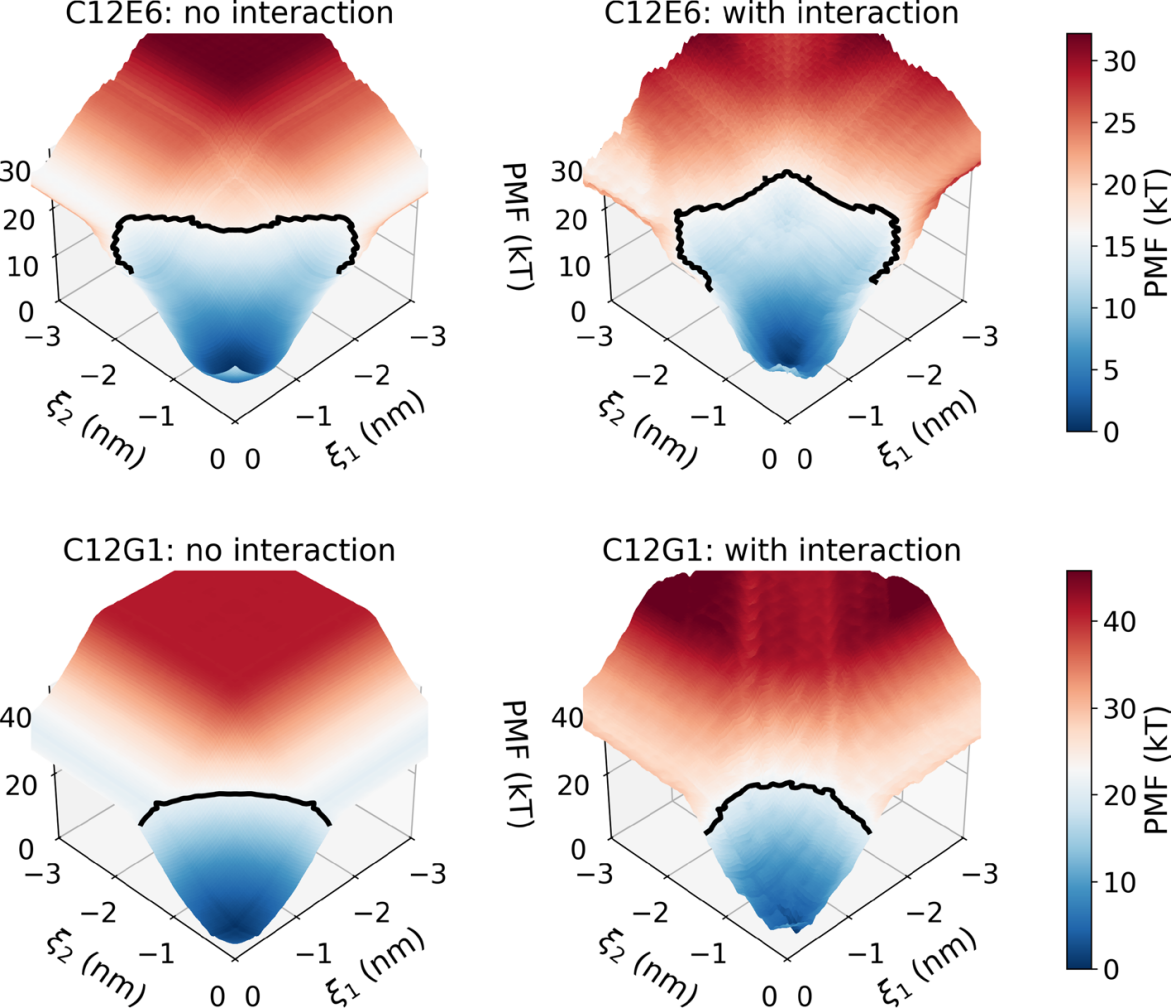
The potential of mean force (PMF) used to estimate $\Delta G^\textrm{penetration2}$ (top panels) for C$_{12}$E$_{6}$ and (bottom panels) for C$_{12}$G$_{1}$. (Left panels) PMFs computed for the ideal case without interactions between the two surfactant molecules. (Right panels) PMFs computed for the case including interactions between the two surfactant molecules. The black contour lines corresponding to $\textrm{PMF} = \Delta G^{\textrm{penetration1}}_{\textrm{C}_{12}{\textrm{E}}_{6}}$ ($16~\textrm{kT}$) and $\textrm{PMF} = \Delta G^{{\textrm{penetration1}}}_{\textrm{C}_{12}{\textrm{G}}_{1}}$ ($20~\textrm{kT}$) are included to visualize changes in the PMF due to interactions between the two surfactants.

The results however differ when multiple surfactants are present simultaneously. $\Delta G^\textrm{penetration2}$ quantifies the free energy barrier experienced by two surfactant molecules as they penetrate the pore. In this analysis, two reaction coordinates, $\xi _1$ and $\xi _2$, identify the center-of-mass position of surfactant 1 and surfactant 2, respectively, with respect to the pore entrance. The results are summarized in Fig. 5 for C$_{12}$E$_{6}$ and C$_{12}$G$_{1}$. The left panels represent the two-dimensional PMFs used as reference cases, when there is no interaction between surfactants. Interactions between surfactants induce deviations, as shown in the right panels of Fig. 5. In the case of C$_{12}$E$_{6}$, these deviations show that the co-penetration of two surfactant molecules is facilitated ($\xi _1 \approx \xi _2 \rightarrow -3~\textrm{nm}$), which can be appreciated by observing the extended region in which the free energy is below 16 *kT* (black line), which corresponds to $\Delta G^{{\textrm{penetration1}}}_{{\textrm{C}}_{12}{\textrm{E}}_{6}}$. In contrast, for C$_{12}$G$_{1}$, co-penetration is only slightly enabled, as highlighted by the black line marking (21 *kT*), corresponding to $\Delta G^{{\textrm{penetration1}}}_{{\textrm{C}}_{12}{\textrm{G}}_{1}}$, but it is interesting to note that this co-penetration is possible only when the two surfactants are in the same position. Accordingly, the PMF for co-penetration of C$_{12}$E$_{6}$ is expected to decrease with further increasing surfactant number, whereas it remains nearly unchanged for C$_{12}$G$_{1}$.

**Fig. 6:**
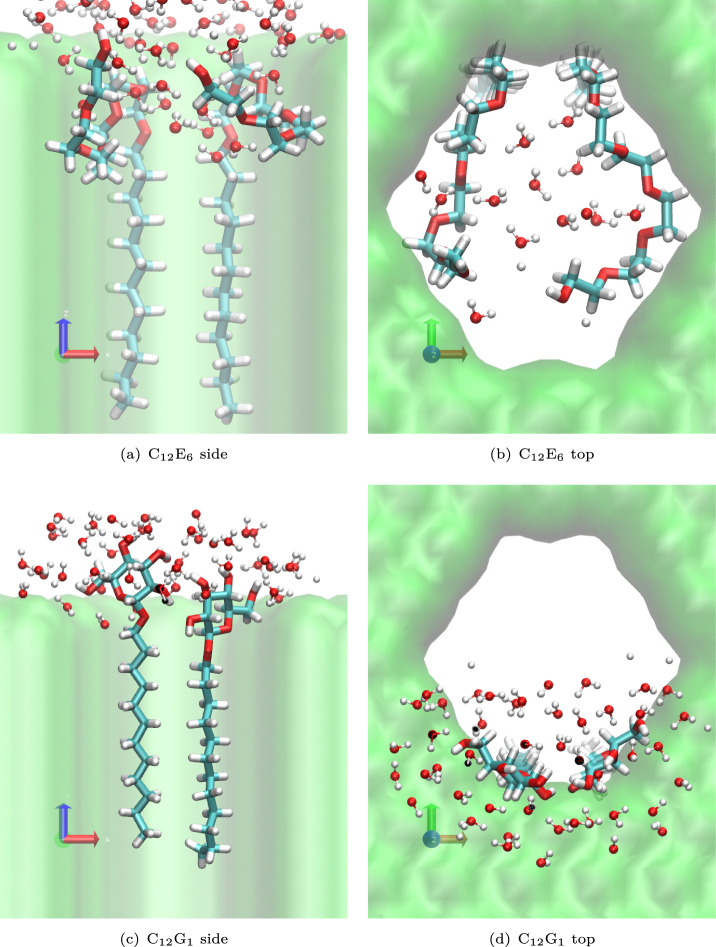
Snapshots illustrating the conformations of two surfactants and their interactions with water near the pore entrance. (**a**) Top view of C$_{12}$E$_{6}$. (**b**) Side view of C$_{12}$E$_{6}$. (**c**) Top view of C$_{12}$G$_{1}$. (**d**) Side view of C$_{12}$G$_{1}$. Red, cyan, and white spheres represent oxygen, carbon, and hydrogen atoms, respectively. Water molecules within 0.5 nm of the surfactants are shown.

Because C$_{12}$E$_{6}$ and C$_{12}$G$_{1}$ surfactants have the same tail groups, the differences in their behavior is likely to be attributed to their different headgroups. C$_{12}$E$_{6}$ presents repeating −CH$_2$–CH$_2$–O− units. By pointing the −CH2–CH$2$− segments toward the pore surface and the oxygen atoms toward the pore center, a small hydrophilic environment can be obtained within the pore, where water molecules can reside, as shown in the top panels of Fig. 6. This makes C$_{12}$E$_{6}$ more likely to penetrate into the pore, dragging water molecules along, especially when multiple surfactants are present. As a result, the mean penetration depth of water molecules increases with the number of surfactants; for one, two, and three surfactants, the values are −0.42 nm, −0.55 nm, and −0.66 nm, respectively, with more negative values indicating deeper penetration. The penetration depth of water is calculated by averaging, over the simulation trajectory, the center-of-mass position of the water molecule that penetrates most deeply into the nanopore. In contrast, the rigid headgroup of C$_{12}$G$_{1}$ cannot form such a hydrophilic environment within the pore. Therefore, the headgroup remains in the aqueous phase near the pore entrance, as shown in the bottom panels of Fig. 6. A second surfactant tends to reside at nearly the same depth, and only a negligible increase in the penetration depth of water molecules is observed with increasing numbers of surfactants; for one, two, and three surfactants, the values are $-0.24$ nm, $-0.27$ nm, and $-0.31$ nm, respectively.

### Surfactants penetration into wax nanopores

**Fig. 7:**
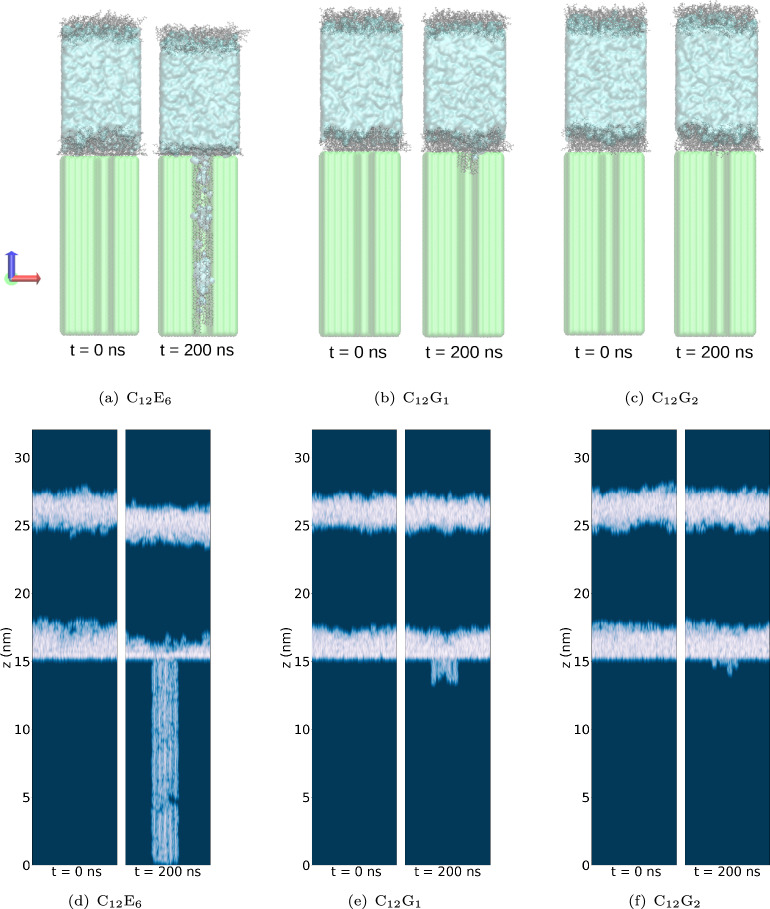
(**a**), (**b**), (**c**): Simulation snapshots showing the penetration of surfactants into the model epicuticular wax layer for (**a**) C$_{12}$E$_{6}$, (**b**) C$_{12}$G$_{1}$, and (c) C$_{12}$G$_{2}$. In each subfigure, the left panel represents the system at the start of the simulation (t = 0 ns), while the right panel shows the system at t = 200 ns. Extending the simulation time to 500 ns did not show any changes in the behavior of C$_{12}$G$_{1}$ and C$_{12}$G$_{2}$. The gray regions correspond to surfactant molecules, the transparent cyan region represents the aqueous phase, and the green transparent region depicts the wax layer. (**d**), (**e**), (**f**): Scans showing the location of molecules for (**d**) C$_{12}$E$_{6}$, (**e**) C$_{12}$G$_{1}$, and (**f**) C$_{12}$G$_{2}$. Surfactant molecules are indicated by the bright regions.

To facilitate Pathway 3, it is necessary that surfactants penetrate the hydrophobic wax nanopores. The likelihood of this phenomenon occurring was probed by conducting extensive non-equilibrium MD simulations for aqueous systems containing C$_{12}$E$_{6}$, C$_{12}$G$_{1}$, and n-dodecyl-$\beta$-D-maltoside (C$_{12}$G$_{2}$) for comparison. The results are shown in the top panels of Fig. 7. The distributions of surfactant molecules at the end of the simulations (t = 200 ns) are displayed in the bottom panels. Out of the three surfactants considered, only C$_{12}$E$_{6}$ spontaneously penetrates into the wax nanopore. This behavior is expected because the presence of multiple surfactant molecules leads to the penetration free energy barrier eventually becoming negative, as discussed above. Although our simulations are short compared to experimental timeframes, extending the simulation time would increase the penetration depth until the concentration gradient vanishes throughout the length of the pore across the wax layer. Consistent with previous findings^57^, and consistent with the weak free energy barriers decrease for multiple surfactants discussed above, the simulation results show that APGs exhibit negligible penetration. Clearly, the surfactant structure determines their ability to spontaneously penetrate existing nanopores. Increasing the length of EO groups from 6 to 8 (C$_{12}$E$_{8}$) has no significant difference in terms of surfactant penetration (results shown in SI, Section H).

### Hydrophilic AI uptake

**Fig. 8:**
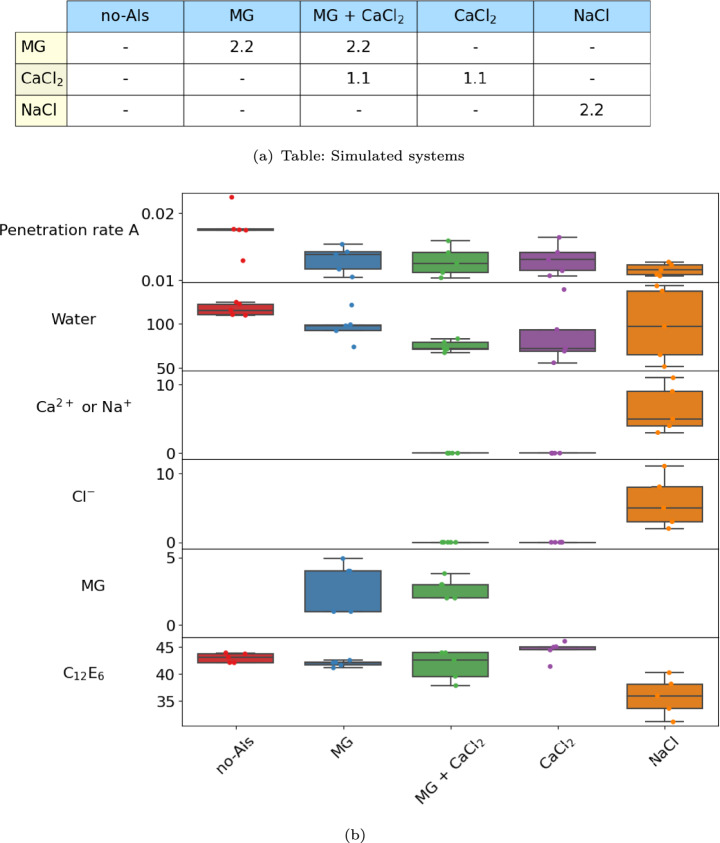
(**a**) A table of the concentration (mol/L$\cdot$H$_2$O) of the compounds (rows) used in each simulated system (column). MG:Methyl-$\beta$-D-Glucose, CaCl$_2$: Ca$^{2+}$ + 2Cl$^{-}$, and NaCl: Na$^{+}$ + Cl$^{-}$. All systems contain C$_{12}$E$_{6}$ at the surface concentration of $\Gamma$ = 3.5 $\times$ 10$^{-6}$ mol/m$^{2}$ both at top (air/solution) and the bottom (leaf/solution) interfaces. (**b**) (column): Simulated systems listed in panel (**a**). Different colors represent the different systems. Each row represent the simulated penetration rate A (in Eqn. (1)) of C$_{12}$E$_{6}$, and number of molecules (Water, Ca$^{2+}$ or Na$^{+}$, Cl$^-$, MG, and C$_{12}$E$_{6}$) uptaken through the nanopore in 200 ns in each system.

**Fig. 9:**
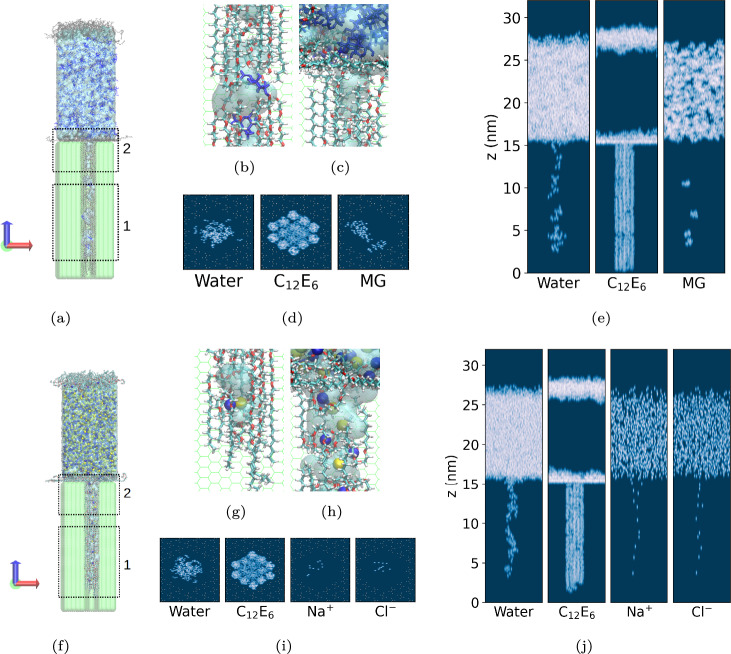
Snapshots at t = 200 ns showing the penetration of AIs and C$_{12}$E$_{6}$ into a cuticular wax film. (**a**)-(**e**) for MG. (**f**)-(**j**) for NaCl. (**a**), (**f**): The entire system, featuring a pore with a depth of 15 nm. The green region represents the leaf wax, blue indicates Na$^+$ or MG, yellow represents Cl$^-$, and grey corresponds to C$_{12}$E$_{6}$. (**b**), (**c**): Close-up views of regions 1, 2 from (**a**). (**g**), (**h**): The close up view of each region 1, 2 from (**f**). (**d**), (**i**): The scan of the location of the each molecule in the system in the top view. (**e**),(**j**): The scan of the location of the each molecule in the system in the side view. In (**b**), (**c**), (**g**), and (**h**), the surfactant atoms are color-coded as cyan for carbon, red for oxygen, and white for hydrogen. Water is shown as a transparent light blue layer.

The penetration of MG, CaCl$_2$, and NaCl through the wax nanopores was modeled in the presence of C$_{12}$E$_{6}$. The systems studied, summarized in Fig. 8 (a), contain C$_{12}$E$_{6}$ at the surface concentration of $\Gamma$ = 3.5 $\times$ 10$^{-6}$ mol/m$^{2}$. The simulation results show that the AIs follow the surfactants into the pore, as illustrated by the snapshots at $t = 200$ ns and by the spatial density distributions shown in Fig. 9.

Experimentally, hydrophilic AIs penetration rate is quantified by the “simulation of foliar uptake (SOFU)” method^25^, according to which - penetration rate data $R(t) = -\ln {(1-\frac{M_{t}}{M_{0}})}$ are plotted as a function of time. $M_{t}$ is the penetrated amount at time t, and $M_{0}$ is the applied AI amount. $R(t) = At$ is usually a linear function of time, where A ($t^{-1}$) is the penetration rate. It is worth observing that SOFU experiments are conducted over up to 100 hours, while MD simulations can only access hundreds of nanoseconds. In the experiments, surfactants are continuously supplied from the bulk phase to the wax surface, while the present simulations maintain the system composition fixed. These differences imply that simulations can only provide results indicative of the phenomena occurring in experimental systems. With this in mind, to interpret the MD results, it is assumed that the uptake amount, $M_{t}$, is proportional to the penetration depth achieved by the surfactants within the wax nanopores, $z(0) - z(t)$, where *z*(*t*) represents the depth reached by the surfactant at time *t*, and *z*(0) corresponds to the position at $t = 0$, on the surface. Under this approximation, AI uptake can be expressed as $M_{t} = B(z(0) - z(t))$, where the coefficient *B* represents the quantity of AIs that penetrate with the surfactants. Accordingly, *z*(*t*) can be casted as:

1 $$\begin{aligned} z(t) = \frac{M_{0}}{B}(1-e^{-At}) \end{aligned}$$

Since the prefactor $\frac{M_{0}}{B}$ is constant, plotting *z*(*t*) as a function of time and fitting it to an exponential function estimates the penetration rate coefficient *A*. The procedural details are presented in SI (Sections C.3 and E). Penetration rate *A* and AI uptake amount ($M_\mathrm{t=200~ns}$) for each system considered are shown in Fig. 8 (b). While *A* represent the speed of penetration of C$_{12}$E$_{6}$, $M_\mathrm{t=200ns}$ represent the total uptake amount.

Simulation snapshots in Fig. 9 reveal discrete water clusters formed within the hydrophobic nanopores. These ’discrete water clusters’ are nanodroplets of water molecules stabilized by the hydrophilic head groups of C$_{12}$E$_{6}$. They are not connected to the aqueous phase deposited on the wax surface, yet they provide an environment that stabilizes hydrophilic AIs. Charge neutrality is maintained within the pores when NaCl is considered as the number of cations and anions is balanced. In contrast, CaCl$_2$ was unable to penetrate the pore even when C$_{12}$E$_{6}$ was present. Adding CaCl$_2$ to the aqueous solution did not change significantly the penetration of MG nor that of C$_{12}$E$_{6}$. On the other hand, NaCl penetrates the nanopores, indicating that co-solvation with the surfactant plays an important role to explain ion-specific effects. Indeed, Na$^{+}$ is well solvated in C$_{12}$E$_6$, as shown in Fig. 9(h), and as previously demonstrated in Ref^76^ with EO-based surfactants. In contrast, CaCl$_2$ shows no solvation of Ca$^{2+}$ near the C$_{12}$E$_6$ headgroup, and Cl$^{-}$ is repelled by the negatively charged oxygen atoms (Figures F.6 (h) and G.10 (b) in SI).

The penetration rate *A* decreases upon the addition of AIs. However, when comparing the total C$_{12}$E$_{6}$ uptake, differences are trivial when MG or CaCl$_{2}$ are present. Since much C$_{12}$E$_{6}$is expected to absorb within the amorphous part of the wax, these small difference may not be detectable experimentally, which is consistent with results presented in Ref^57^. In contrast, when a significant amount of AIs penetrates together with the surfactant, the amount of C$_{12}$E$_{6}$ within the nanopore is noticeably reduced, presumably because of steric effects.

### Antagonistic effect due to calcium ions

Changes in surface tension are unlikely to explain observed antagonistic effects, as MG penetration shows no significant difference in the presence of CaCl$_2$. Furthermore, the solvation free energy of MG in a CaCl$_2$ solution does not differ significantly from that in pure water, indicating no preferential interactions between Ca$^{2+}$ and MG (see Figure F.1 in SI). It could be speculated that preferential interactions between AEs and Ca$^{2+}$ could be responsible for the low adsorption of AEs on the wax surface. To probe this possibility, changes in adsorption free energy were calculated for C$_{12}$E$_{6}$ due to the addition of CaCl$_2$. The results indicate that C$_{12}$E$_{6}$ is more strongly adsorbed on the leaf wax when CaCl$_2$ is present (see Figure F.3 and Figure F.6 (i)in SI), excluding this mechanism as a possibility. It could be that C$_{12}$E$_{6}$ surfactants assemble more strongly in CaCl$_2$ solutions compared to pure water, increasing their hydrodynamic radius, which in turn slows diffusion. Indeed, the aggregation free energy of C$_{12}$E$_{6}$ decreases in the presence of CaCl$_2$, indicating an increased preference for aggregation (see Figure F.3 and Figure F.6, (c) and (d), in SI). To test whether these differences are ion-specific, NaCl solutions were also simulated at the same ionic strength (3.3 mol/L). The results show that C$_{12}$E$_{6}$ more preferably aggregate in presence of NaCl than CaCl$_2$ (compare Figure F.3 and Figure F.6 (d) and (e) in SI), ruling out aggregation as the primary cause responsible for antagonistic effects. Another possible explanation, such as the slow diffusion of C_12_E_6_ due to the presence of CaCl$_2$, is also excluded, since the increase in viscosity is not specific to CaCl$_2$ (see Figure F.4 in the SI).

**Fig. 10:**
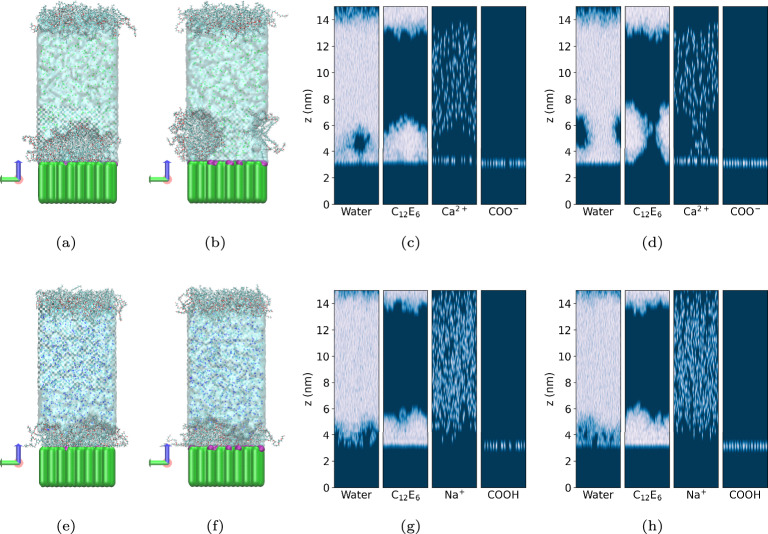
(**a**) and (**b**): Simulation snapshots obtained at t = 100 ns showing the deformation, or detachment, of C$_{12}$E$_{6}$ surfactant films initially adsorbed on the model leaf wax in 1.1 mol/L CaCl$_{2}$ solution. Results are presented for different surface properties: (a) for COO$^{-}$ 0.5 %, (b) for COO$^{-}$ 1.5 %. (**e**) and (**f**): Simulation snapshots obtained at t = 100 ns showing the negligible deformation of C$_{12}$E$_{6}$ surfactant films initially adsorbed on the model leaf wax in 3.3 mol/L NaCl solution, (**e**) for COOH 0.5 %, and (**f**) COOH 1.5 %. The green region represents the leaf wax, purple indicates COO$^{-}$ or COOH groups, and gray represents C$_{12}$E$_{6}$. (**c**), (**d**): The scan of the location of the each molecule in the system in the side view for (**a**) and (**b**), respectively. (**g**),(**h**): The scan of the location of the each molecule in the system in the side view for (**e**) and (**f**), respectively.

The remaining possibility is a change in surface properties due to Ca$^{2+}$ ions. To investigate this hypothesis, a surface was prepared that exposes carboxyl groups (-COOH or -COO$^{-}$). Surfaces were prepared in which 0.5% and 1.5% of the CH$_3$/CH$_2$ groups were replaced by COO$^{-}$ groups, for the case of 1.1 mol/L CaCl$_{2}$ solution, and by COOH group for 3.3 mol/L NaCl as depicted in Fig. 10 (a), (b), (e) and (f). This surface density is realistic, as discussed in the Methods section ("The model epicuticular wax on plant leaves"). To probe the stronger binding affinity of Ca$^{2+}$ ions compared to C$_{12}$E$_{6}$, once C$_{12}$E$_{6}$ film was pre-formed on the surface then CaCl$_{2}$ ions were introduced. The structural transition of the adsorbed surfactant film was monitored over 100 ns. The results are presented in Fig. 10 (a)-(d).

The simulation results show that Ca$^{2+}$ ions bound to the surface COO$^{-}$ groups are hydrated, which hampers the adsorption of C$_{12}$E$_{6}$. At 0.5$\%$ COO$^{-}$, the deformation of the C$_{12}$E$_{6}$ monolayer is observable in panels (a) and (c). Further increasing the concentration of COO$^{-}$ to 1.5 % results in the detachment of the surfactant film from the surface. This mechanism is likely responsible for the antagonistic effect due to Ca$^{2+}$ observed experimentally^57,58^.

The results obtained when the surface functional groups were COOH instead of COO$^{-}$ are shown in Fig. 10 (e)-(h). To demonstrate that this antagonism occurs only in the presence of divalent cations, a 3.3 mol/L NaCl solution was also tested. As discussed in the Methods section ("The model epicuticular wax on plant leaves"), H$^{+}$ binds more strongly to the COO$^{-}$ group than Na$^{+}$ ions; therefore, the carboxyl group is considered protonated (COOH). Under these conditions, a slight deformation of the C$_{12}$E$_{6}$ film is observed, but not the detachment of the C$_{12}$E$_{6}$ film even at the highest COOH concentration considered, suggesting that antagonistic effects are absent for Na$^{+}$ ions. These findings are further supported by the changes in the adsorption free energy of C$_{12}$E$_{6}$ in the presence of Ca$^{2+}$ ions bound to the surface. Specifically, surfaces functionalized with COO$^{-}$ groups and bound Ca$^{2+}$ exhibit positive adsorption free energies (+5 to +10 *kT*), promoting desorption of C$_{12}$E$_{6}$. In contrast, surfaces functionalized with COOH groups show negligible differences from the clean surface, with adsorption free energies around –12.5 *kT*, indicating favorable adsorption of C$_{12}$E$_{6}$ (see F.6 and Figure F.9 (d) in SI). Simulation results suggest that Ca$^{2+}$ ions adsorb primarily onto the surface even when the functional groups are inside the pores, because of long-ranged electrostatic interactions (see Figure F.9 (d) in SI).

## Discussion

The simulation results presented above show that APGs (C$_{12}$G$_{1}$, C$_{12}$G$_{2}$, or their mixtures) do not spontaneously penetrate wax nanopores of size comparable to those predicted to exist in the epicuticular layers (< 2.4 nm^33^), which is consistent with experimental findings^57^. For individual surfactants, our free energy calculation shows weaker adsorption for C$_{12}$G$_{1}$ than C$_{12}$E$_{6}$ on the model leaf wax surface, as illustrated in Fig. 4.

The free energy profiles representing penetration into the nanopore suggest a higher hydrophilic nature for C$_{12}$G$_{1}$. This may not be the reason for the reduced penetration of C$_{12}$G$_{1}$, because penetration still occurs for AEs even when the hydrophilic headgroup is increased to C$_{12}$E$_{8}$ (see Figure H.15 in SI).

These data suggest lack of uptake, which might be attributed to slightly higher surface tension (ST) of C$_{12}$G$_{1}$ compared to C$_{12}$E$_{6}$. However, even when the surface tension of systems containing fewer C$_{12}$E$_{6}$ is higher than that of systems containing C$_{12}$G$_{1}$, C$_{12}$E$_{6}$ penetrates nanopores. This suggests that factors other than surface tension govern uptake.

Therefore, the propensity for penetration into the nanopore is likely governed by surfactant-specific interactions with the nanopore. The free energy calculations for two surfactant molecules reveal that co-penetration of the surfactants is facilitated as the number of surfactant molecules increases for the case of C$_{12}$E$_{6}$. C$_{12}$E$_{6}$ forms a localized hydrophilic environment within the nanopore by accommodating water molecules through its flexible, partially hydrophobic headgroup, thereby promoting further surfactant penetration. This mechanism is absent for C$_{12}$G$_{1}$. Consequently, only specific accelerator surfactants, such as AEs (or TBP), activate the third uptake Pathway shown in Fig. 1.

Our results show that MG, by itself, is not well solvated into C$_{12}$E$_{6}$ layer on the leaf wax, which is consistent with experimental data^18^. The higher solvation free energy of MG into the film of C$_{12}$E$_{6}$ surfactants on the model leaf wax than in aqueous phase further supports this possibility (see Figure G.10 (b) in SI). Therefore, water clustering within wax nanopores, promoted by C$_{12}$E$_{6}$, is necessary to facilitate MG uptake. Furthermore, the slightly positive surface adsorption free energy for MG hinders MG adsorption on the leaf wax when it penetrates the nanopore with AEs (see Figure G.10 (a) in SI). Ions that are well-solvated in C$_{12}$E$_{6}$ such as Na$^{+}$ can penetrate the pores by overcoming their relatively weak surface adsorption.

The antagonistic effects due to calcium salts on MG uptake^57,77^ can be explained by the binding of Ca$^{2+}$ ions, which hinder the adsorption of C$_{12}$E$_{6}$, thereby slowing its penetration. The possible reasons for the experimentally observed slow penetration of C$_{12}$E$_{8}$^57,58,77^ (or C$_{12}$E$_{6}$) in the presence of Ca$^{2+}$ could include the strong interaction between MG and Ca$^{2+}$ ions, increased aggregation of C$_{12}$E$_{6}$, an increase in surface tension due to CaCl$_{2}$, or slow diffusion of C$_{12}$E$_{6}$ in CaCl$^2$ solutions. However, our simulations show that none of these factors alone can definitively explain the slow uptake of C$_{12}$E$_{6}$. Therefore, the possible reason is the change of surface properties in the presence of Ca$^{2+}$, which does not occur with monovalent cations^58^. The simulation results clearly show strong binding of Ca$^{2+}$ ions onto COO$^{-}$ functional groups on the model leaf wax. These surface Ca$^{2+}$ ions reduce the area where C$_{12}$E$_{6}$ can adsorb. On the other hand, the protonated COOH group does not show any significant change on the structure of C$_{12}$E$_{6}$ monolayer on the surface. Since monovalent ions are bound to COO$^{-}$ much less than Ca$^{2+}$, most of the COO$^{-}$ groups are solvated by H$^{+}$ as H$^{+}$ has the strongest binding in the case of the plant root surface^71^. This is consistent with the pH effect in the presence of various cations which indicated the strong binding of Ca$^{2+}$ ions on COO$^{-}$ groups^67^.

Pathway 3 should be distinguished from the conventional hydrophilic pathway (Pathway 2), when experimental observations are to be interpreted. Since CaCl$_2$ alone does not penetrate the wax nanopores, a significant portion must follow uptake via Pathway 2 localized near cuticular ledges of guard cells, basal cells of trichomes, and anticlinal walls^32^. Both APGs and AEs likely enhance CaCl$_2$ uptake through these hydrophilic pathways^54^. Antagonistic effects have been documented for MG in the presence of TBP and DESU^52^; the analysis of simulation results suggests that these effects may be attributed to multiple mechanisms. For instance, TBP and diethyl sebacate (DES), similar to DESU, exhibit an antagonistic effect on CaCl$_2$ uptake, which exclusively engages via Pathway 2^54^. In this context, the penetration of CaCl$_2$ after 100 hours with only APG was 95%, while the addition of TBP or DES reduced it to 33% and 18%, respectively. DESU does not enhance MG penetration through wax nanopores, leading to stronger antagonism compared to TBP.

The findings presented here can contribute to the optimal formulation of surfactant/AI combinations for efficient AI uptake via foliar spray, thereby enhancing sustainable crop growth.

## Methods

### Molecular dynamics simulations

All simulations were carried out using the GROMACS 2021.5 software package^78–80^. The force field parameters were carefully selected by comparing the surface tension (ST) and interfacial tension (IFT) with available experimental data. In this study, we employed the combination of the OPLS/2020 force field^81,82^ and the TIP4P/2005 water model. Reliability of this combination of force fields, as well as details of the subsequent simulations, are provided in SI (C). All the simulations are performed under ambient conditions (1 bar, 300 K). It should be noted that, in all simulations, neither the actual water droplet on the leaf nor the evaporation of water was considered, as these would require large-scale simulations. Instead, we focused on a portion of the droplet where a nearly flat aqueous phase is formed on the leaf wax.

### The model epicuticular wax on plant leaves

As a model leaf, we consider the outermost layer of the cuticle proper, the epicuticular wax. Since the simulation is conducted at a scale of several tens of nanometers, we do not account for the micrometer-scale surface roughness resulting from the three-dimensional structure of the epicuticular wax crystals. This roughness primarily governs properties such as wettability, adhesivity, and self-cleaning behavior of the leaf wax^83^.

The model leaf consists of alkanes with an orthorhombic symmetric crystalline structure, as described in Refs^84,85^. While real plant epicuticular wax is composed of various compounds with differing chain lengths and functional groups^28,30,32,63,86–91^, including esters, fatty alcohols, and fatty acids, we simplify our model by considering only alkanes. Given that the majority of epicuticular wax consists of CH$_2$ and CH$_3$ groups from hydrocarbon chains (97%), with polar components such as OH or COOR groups accounting for less than 3%^28,32,92,93^, this model is expected to capture the fundamental properties of epicuticular wax. The hydrocarbon chains are predominantly oriented perpendicular to the surface, as indicated by Raman imaging^94^ and polarization modulation-infrared reflection-absorption spectroscopy (PM-IRRAS)^95^. Therefore, the topmost surface of the plant epicuticular wax primarily consists of terminal CH$_3$ groups of alkanes. For convenience, we define this surface as the (001) surface. In our study we only consider this surface as depicted in Fig. 3 (a) and (b). For the adsorption simulations without a pore, the total size of the unit cell of the model leaf is 6 nm $\times$ 6 nm $\times$ 3 nm. A depth of 3 nm was chosen to reduce computational cost while still capturing the essential interactions between the molecules in the liquid phase above the leaf and the leaf wax. Periodic boundary conditions are applied along xy directions parallel to the surface, resulting in an infinite slab of the leaf wax. In addition to the (001) surface, other surface orientations can also be considered to account for different structural configurations. The surfaces exposing the hydrocarbon chains of the alkane to the liquid phase have two possible configurations, as illustrated in Fig. B.2 (c), (d), (e), and (f) in SI. These surfaces are defined as the (100) and (010) surfaces, respectively. While the adsorption free energy of surfactants may vary across the (001), (100), and (010) surfaces, the fundamental adsorption mechanism of the surfactants remains consistent across these orientations (results not shown).

To study ion binding, some of the terminal CH$_3$ groups on top or on the bottom of the surface, depicted in Fig. 3 are replaced by COO$^{-}$ or COOH. To neutralize the positive charge experimentally observed by the number of bound Ca$^{2+}$ (2.73 nmol/cm$^{2}$)^69^, more than 2% of total carbons (CH$_2$ or CH$_3$) have to be replaced by COO$^{-}$ groups. Although this represents a relatively high number for simulations, the strong antagonistic effect can already be observed at approximately 1.5% replacement of CH$_3$ groups. Since the total amount of the functional groups are less than 3% including the saturated COOR groups^28,32,92,93^, 1.5 % replacement with unsaturated COO$^{-}$ group may be a reasonable choice or higher. Ion binding is negligible for monovalent ions such as Rb$^{+}$ compared to Ca$^{2+}$ due to their weaker interaction with COO$^{-}$ groups relative to H$^{+}$ ions dissociated from COOH groups. Incorporating the dynamics of H$^{+}$ ions in classical MD simulations is challenging due to their short lifetime ($\sim$ps) interacting with other water molecules. Therefore, to enable a systematic comparison of the influence of Ca$^{2+}$, we approximate that the COO$^{-}$ groups are protonated to COOH. The absence of a pH-dependent effect on AE penetration^96,97^ and spreading on the hydrophobic surfaces^98^ confirms the validity of this approximation.

All wax molecules were kept frozen during the simulations except for the COO$^{-}$ or COOH groups. The thermal vibrations of the wax molecules showed no significant differences within the penetration time frame discussed later. Dewaxing was also not considered, as no diffusion of wax molecules into the surfactant solutions was observed without constraints on the wax molecules within the simulation timescale (200 ns). Considering that the dewaxing process typically takes several minutes^47^, this assumption is reasonable.

### Nano-scale pore within the epicuticular wax

As ionic solutes and lipophilic solutes are believed to traverse different pathways, we consider the presence of voids or pores within or between crystalline wax structures, where surfactants can penetrate^55^. Assuming the pore size is similar to pathway 2, the estimated pore radii range from $r = 0.3 \sim 2.4$ nm^33^. Small pores ($r < 0.4$ nm) are excluded from this study, as only surfactant molecules could occupy such pores, with other solutes unable to penetrate. Instead, we focus on pore radii of approximately $r = 0.75 \sim 0.8$ nm, corresponding to the absence of approximately fourteen alkane molecules within the crystalline wax, as illustrated in Fig. 3 (c). Naturally, larger pore sizes could lead to higher penetration rates for AIs, assuming similar behavior occurs with smaller pores. To study the penetration of molecules from the liquid phase above the surface into the pore, as well as their diffusion into the inner part of the wax, the depth of the pore was set to 15 nm, corresponding to approximately four layers of alkane wax. Each layer has an approximate thickness of 4 nm^62^, which corresponds to an average of 30 carbons per alkyl chain. Dividing the pore into four layers revealed no significant differences in the penetration behavior of the molecules. The structure of the leaf with a pore is depicted in Fig. 3 (c) and (d). It is worth noting that various pore shapes, such as slit-like or zigzag configurations, could potentially influence the AI uptake. However, this study focuses on a circular pore shape to capture the fundamental behavior of molecular diffusion into the pore.

### Surfactants and active ingredients on the leaf wax

The concentration of AIs, *c* (mol/L), within a sessile droplet deposited on the leaf wax, and the adsorbed amount of surfactants at the leaf wax, $\Gamma$ (mol/m$^2$), were determined as follows. The adsorbed amount of surfactant varies depending on the molecular size and surface activity of the compounds. For C$_{12}$E$_{6}$, the adsorption onto the wax surface has been estimated at different concentrations, as reported in Ref^47^. The maximum adsorption is observed at the critical micelle concentration (CMC) of C$_{12}$E$_{6}$, beyond which the wax begins to dissolve due to the surfactant. Assuming that the saturated amount of surfactants is comparable between the air/water interface and the solid/water interface for surfactant monolayers^99^, the saturated surface densities can be estimated using experimental data from either the air/water or solid/water interfaces. In the simulation, the concentration of surfactants at the interface is determined by the surface surfactant density, where the ST and IFT closely match the experimental values. The surface surfactant density and corresponding surface tension values are summarized in Table 1. Details of the calculations for ST and IFT are provided in SI (C.2).

In most experiments investigating the penetration of hydrophilic AIs, AIs are added to the original water droplet in concentrations of the same order of magnitude or higher (10$^{-1}$ $\sim$ 1 g/L or mM)^18,54,57,77,100–103^. The uptake of ionic or hydrophilic solutes typically begins as the evaporation of the water droplet nears completion^35,77,101,104^, while the remaining deposit is still in a liquid state. Consequently, it can be assumed that the uptake process commences under conditions of very high concentrations of AIs and surfactants. In this study, AIs are added to the aqueous phase at molar ratios of AI:surfactant as follows: 8:3 for C$_{12}$E$_{6}$, 1:1 for C$_{12}$G$_{1}$, and 5:2 for C$_{12}$G$_{2}$. This corresponds to an approximate concentration of $c_\textrm{AI} = 2.2$ mol/L for the AIs, with saturated surfactant monolayers present at both the water/air and air/leaf interfaces at CMC. At this concentration, the solution remains in a liquid state with sufficient fluidity to allow diffusion within a reasonable computational timeframe, up to 200 ns. Although the concentration of AIs may influence the total penetration amount, even above the CMC of the surfactant^18,52,56,77^, the primary objective here is to purely investigate the effects of surfactants. This is achieved by maintaining the same AI concentration across different surfactant mixtures.

**Table 1:** Experimental (at CMC) vs simulated values of $\Gamma$ ($\times$ 10$^{-6}$ mol/m$^{2}$), ST, and IFT (mN/m). The IFT is calculated at water/octane interface for C$_{12}$E$_{6}$, and at water/decane interface for C$_{12}$G$_{1}$ and C$_{12}$G$_{2}$. $^{*a}$From Refs^47,105–108^. $^{*b}$From Refs^108,109^. $^{*c}$From Ref^110^. $^{*d}$From Refs^41,111^. $^{*e}$From Refs^108,109^. $^{*f}$From Refs^41,42^. $^{*g}$From Ref^99^. $^{*h}$From Refs^38–40^. $^{*i}$From Ref^42^.

		$$\Gamma$$ ($$\times$$ 10$$^{-6}$$ mol/m$$^{2})$$	ST (mN/m)	IFT (mN/m)
C$_{12}$E$_{6}$	Experiment	3.2 $\sim$ 3.7$^{*a}$	32 $\sim$ 33$^{*b}$	3$^{*d}$
	Simulation	3.5	31.0 ± 0.7	1.5 ± 1.3
C$_{12}$G$_{1}$	Experiment	3.3 $\sim$ 5.5	28 $\sim$ 38$^{*e}$	0.5$^{*f}$
	Simulation	4.8	37.3 ± 0.9	3.7 ± 1.5
C$_{12}$G$_{2}$	Experiment	3.7	35 $\sim$ 36$^{*h}$	3$\sim$ 5$^{*i}$
	Simulation	3.8	37.2 ± 2.7	2.3 ± 2.4

### Adsorption/penetration free energy calculations

The adsorption/penetration free energy profiles discussed in 2.1 was calculated using the accelerated weight histogram (AWH) method^112,113^ (see D.2 in SI for simulation parameters). In this method, bias potentials are applied on the fly during the simulation to update the potential of mean force (PMF) until it converges, thereby enabling efficient sampling of high free-energy regions. This approach does not suffer from directional differences between desorption and adsorption processes, particularly in the case of two-surfactant penetration. Simulations were run until the PMFs converged, requiring approximately $500~\textrm{ns}$ for adsorption and single-surfactant penetration, and $1~\mu \textrm{s}$ for two-surfactant penetration. Due to its rigid structure, the required simulation time is shorter for C$_{12}$G$_{1}$ than for C$_{12}$E$_{6}$.

**Fig. 11:**
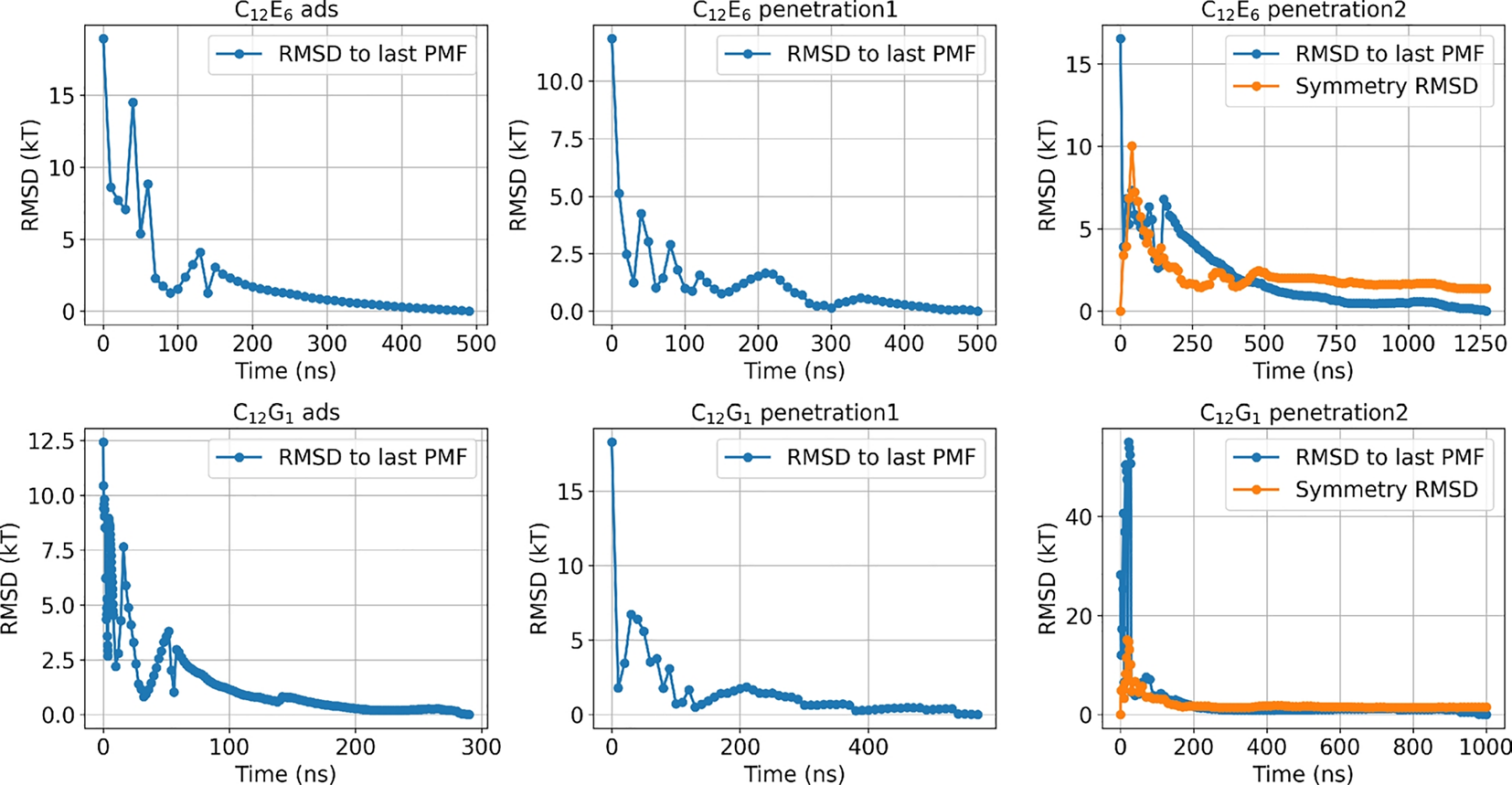
RMSDs relative to the last PMFs and the symmetry RMSDs for C$_{12}$E$_{6}$ (top panels) and C$_{12}$G$_{1}$ (bottom panels). From left to right, the panels show adsorption, single-surfactant penetration, and two-surfactant penetration. Blue lines represent the RMSD, and orange lines represent the symmetry RMSD ($RMSD_\textrm{symm}$).

Convergence was assessed by calculating the root mean square deviation (RMSD) of the PMF as

2 $$\begin{aligned} RMSD(t) = \sqrt{\sum _{\vec {\xi }}\frac{\left( PMF(\vec {\xi }, t) - PMF_\textrm{ref}(\vec {\xi })\right) ^2}{N}}, \end{aligned}$$

where $\vec {\xi }$ spans the grid points of the reaction coordinates at which the PMF is evaluated, and $PMF_\textrm{ref}(\vec {\xi })$ denotes the final PMF value, at which changes in the PMF become negligible, as shown in Fig. 11.

Furthermore, for the case of two surfactant molecules, the reaction coordinates $\xi _1$ and $\xi _2$ are expected to be symmetric. Therefore, instead of performing multiple independent simulations, symmetry convergence was evaluated by calculating the RMSD as

3 $$\begin{aligned} RMSD_\textrm{symm}(t) = \sqrt{\sum _{\xi _1, \xi _2}\frac{\left( PMF(\xi _1, \xi _2, t) - PMF(\xi _2, \xi _1, t)\right) ^2}{N}}. \end{aligned}$$

For all RMSD calculations, the minimum of the PMF was shifted to 0 kT.

Due to the nature of the AWH method, a single simulation yields a converged PMF without an explicit error estimate. To assess the accuracy, a second independent AWH simulation was performed for the penetration of a single surfactant molecule, yielding a difference in $\Delta G^\textrm{ads}$ of approximately 1.5 kT. Combined with the $RMSD_\textrm{symm}$ values, an overall uncertainty of approximately $\pm 1.5~kT$ is expected.

Additionally, thermodynamic integration (TI)^114^ and the umbrella sampling method^115,116^ are used for several free energy calculations in the SI (see Sections D.1 and D.2).

It is important to note that the presence of AIs and additional surfactants may influence the PMF values. However, the primary objective of this study is to estimate the adsorption and penetration behavior of an individual surfactant molecule, driven predominantly by its intrinsic chemical structure.

### Penetration of molecules into the model epicuticular wax

The penetration of molecules begins when a water droplet of several microliters dries out within 0.5 to 1 hour^35,77,101,104^. Meanwhile, the equilibration of the dynamic surface tension (DST) or IFT occurs on the order of $10^2$ to $10^3$ seconds for AEs^117,118^ and on the order of $10^1$ seconds for APGs^39,119^. The presence of ions may influence the DST for ionic surfactants but has negligible effects on nonionic surfactants above the CMC^118^. Consequently, by the time penetration begins, it can be assumed that the surfactants are adsorbed onto the surface. The snapshots are depicted in Figure C.4 in SI. In the simulations, already equilibrated solutions on the flat leaf depicted in Fig. 3 (a), were placed on top of the model leaf with a pore depicted in Fig. 3 (c). The penetration of molecules into the pore was then monitored for a minimum of 200 ns. This simulation duration was sufficient to observe significant differences in penetration behavior of surfactants as well as AIs.

## Supplementary Information

Supplementary Information.

## Data Availability

Data generated during the current study are available from the corresponding author on reasonable request.

## References

[1] van Dijk, M., Morley, T., Rau, M. L. & Saghai, Y. A meta-analysis of projected global food demand and population at risk of hunger for the period 2010–2050. *Nat. Food* **2**, 494–501. 10.1038/s43016-021-00322-9 (2021). 37117684 10.1038/s43016-021-00322-9

[2] Tilman, D., Balzer, C., Hill, J. & Befort, B. L. Global food demand and the sustainable intensification of agriculture. *Proc. Natl. Acad. Sci. U. S. A.* **108**, 20260–20264 (2011). 22106295 10.1073/pnas.1116437108 PMC3250154

[3] Ishfaq, M. et al. Foliar nutrition: Potential and challenges under multifaceted agriculture. *Environ. Exp. Bot.* **200**, 104909 (2022).

[4] Fernandez, V., Gil-Pelegrin, E. & Eichert, T. Foliar water and solute absorption: An update. *Plant J.* **105**, 870–883 (2021). 33219553 10.1111/tpj.15090

[5] de Perre, C. et al. Assessing the fate and effects of an insecticidal formulation. *Environ. Toxicol. Chem.* **34**, 197–207 (2015). 25331413 10.1002/etc.2786

[6] Reddy, K. N. & Locke, M. A. Imazaquin spray retention, foliar washoff and runoff losses under simulated rainfall. *Pestic. Sci.* **48**, 179–187 (1996).

[7] Wauchope, R. D., Johnson, W. C. & Sumner, H. R. Foliar and soil deposition of pesticide sprays in peanuts and their washoff and runoff under simulated worst-case rainfall conditions. *J. Agric. Food Chem.* **52**, 7056–7063. 10.1021/jf049283v (2004). 15537318 10.1021/jf049283v

[8] Andrew H. Cobb, J. P. R. Herbicide Uptake and Movement, Ch. 3, 50–69 https://onlinelibrary.wiley.com/doi/abs/10.1002/9781444327793.ch3 (John Wiley & Sons Ltd, 2010).

[9] Fernandez-Raga, M. et al. Splash erosion: A review with unanswered questions. *Earth Sci. Rev.* **171**, 463–477 (2017).

[10] Sosnoskie, L. M., Culpepper, A. S., Braxton, L. B. & Richburg, J. S. Evaluating the volatility of three formulations of 2,4-d when applied in the field. *Weed Technol.* **29**, 177–184 (2015).

[11] Bish, M., Oseland, E. & Bradley, K. Off-target pesticide movement: A review of our current understanding of drift due to inversions and secondary movement. *Weed Technol.* **35**, 345–356 (2021).

[12] Peterson, M. A., McMaster, S. A., Riechers, D. E., Skelton, J. & Stahlman, P. W. 2,4-d past, present, and future: A review. *Weed Technol.* **30**, 303–345 (2016).

[13] Magnoli, K., Carranza, C. S., Aluffi, M. E., Magnoli, C. E. & Barberis, C. L. Herbicides based on 2,4-d: Its behavior in agricultural environments and microbial biodegradation aspects. A review. *Environ. Sci. Pollut. Res. Int.* **27**, 38501–38512. 10.1007/s11356-020-10370-6 (2020). 32770339 10.1007/s11356-020-10370-6

[14] Hunsche, M., Damerow, L., Schmitz-Eiberger, M. & Noga, G. Mancozeb wash-off from apple seedlings by simulated rainfall as affected by drying time of fungicide deposit and rain characteristics. *Crop Prot.* **26**, 768–774 (2007).

[15] Pimentel, D. Amounts of pesticides reaching target pests: Environmental impacts and ethics. *J. Agric. Environ. Ethics* **8**, 17–29. 10.1007/BF02286399 (1995).

[16] Kerstiens, G. Cuticular water permeability and its physiological significance. *J. Exp. Bot.* **47**, 1813–1832. 10.1093/jxb/47.12.1813 (1996).

[17] Baur, P., Marzouk, H., Schönherr, J. & Grayson, B. T. Partition coefficients of active ingredients between plant cuticle and adjuvants as related to rates of foliar uptake. *J. Agric. Food Chem.* **45**, 3659–3665. 10.1021/jf970233i (1997).

[18] Baur, P., Schonherr, J. & Grayson, B. T. Polydisperse ethoxylated fatty alcohol surfactants as accelerators of cuticular penetration. 2: Separation of effects on driving force and mobility and reversibility of surfactant action. *Pestic. Sci.* **55**, 831–842 (1999).

[19] Buchholz, A. Characterization of the diffusion of non-electrolytes across plant cuticles: Properties of the lipophilic pathway. *J. Exp. Bot.* **57**, 2501–2513. 10.1093/jxb/erl023 (2006). 16829545 10.1093/jxb/erl023

[20] Burghardt, M., Schreiber, L. & Riederer, M. Enhancement of the diffusion of active ingredients in barley leaf cuticular wax by monodisperse alcohol ethoxylates. *J. Agric. Food Chem.* **46**, 1593–1602. 10.1021/jf970737g (1998).

[21] Jeffree, C. E. The Fine Structure of the Plant Cuticle, Ch. 2, 11–125 https://onlinelibrary.wiley.com/doi/abs/10.1002/9780470988718.ch2 (John Wiley & Sons Ltd, 2006).

[22] Kirkwood, R. C. Recent developments in our understanding of the plant cuticle as a barrier to the foliar uptake of pesticides. *Pestic. Sci.* **55**, 69–77 (1999).

[23] Liu, Z. Effects of surfactants on foliar uptake of herbicides – A complex scenario. *Colloids Surf. B. Biointerfaces* **35**, 149–153 (2004). 15261025 10.1016/j.colsurfb.2004.02.016

[24] Schonherr, J. Effects of monodisperse alcohol ethoxylates on mobility of 2,4-d in isolated plant cuticles. *Pestic. Sci.* **38**, 155–164 (1993).

[25] Schonherr, J. & Baur, P. Modelling penetration of plant cuticles by crop protection agents and effects of adjuvants on their rates of penetration. *Pestic. Sci.* **42**, 185–208 (1994).

[26] Schreiber, L., Riederer, M. & Schorn, K. Mobilities of organic compounds in reconstituted cuticular wax of barley leaves: Effects of monodisperse alcohol ethoxylates on diffusion of pentachlorophenol and tetracosanoic acid. *Pestic. Sci.* **48**, 117–124 (1996).

[27] Zeisler-Diehl, V. V. et al. Alcohol ethoxylates enhancing the cuticular uptake of lipophilic epoxiconazole do not increase the rates of cuticular transpiration of leaf and fruit cuticles. *J. Agric. Food Chem.* **70**(3), 777–784. 10.1021/acs.jafc.1c06927 (2022). 35025485 10.1021/acs.jafc.1c06927

[28] Schreiber, L. & Schonherr, J. *Water and solute permeability of plant cuticles* (Springer, 2010).

[29] Popp, C., Burghardt, M., Friedmann, A. & Riederer, M. Characterization of hydrophilic and lipophilic pathways of *Hedera helix* L. cuticular membranes. *J. Exp. Bot.* **56**, 2797–2806. 10.1093/jxb/eri272 (2005). 16143718 10.1093/jxb/eri272

[30] Stagnari, F. A review of the factors influencing the absorption and efficacy of lipophilic and highly water-soluble post-emergence herbicides. *Eur. J. Plant Sci. Biotechnol. 1(1),* 22–35 (2007).

[31] Riederer, M. & MUller, C. Frontmatter, i-xviii https://onlinelibrary.wiley.com/doi/abs/10.1002/9780470988718.fmatter (John Wiley & Sons Ltd, 2006).

[32] Schonherr, J. Characterization of aqueous pores in plant cuticles and permeation of ionic solutes. *J. Exp. Bot.* **57**, 2471–2491. 10.1093/jxb/erj217 (2006). 16825315 10.1093/jxb/erj217

[33] Fernandez, V. & Eichert, T. Uptake of hydrophilic solutes through plant leaves: Current state of knowledge and perspectives of foliar fertilization. *Crit. Rev. Plant Sci.* **28**, 36–68. 10.1080/07352680902743069 (2009).

[34] Schreiber, L. Polar paths of diffusion across plant cuticles. *Ann. Bot.* **95**, 1069–1073. 10.1093/aob/mci122 (2005). 15797897 10.1093/aob/mci122 PMC4246894

[35] Schönherr, J. & Luber, M. Cuticular penetration of potassium salts: Effects of humidity, anions, and temperature. *Plant Soil* **236**, 117–122. 10.1023/A:1011976727078 (2001).

[36] Schönherr, J. & Schreiber, L. Interactions of calcium ions with weakly acidic active ingredients slow cuticular penetration: A case study with glyphosate. *J. Agric. Food Chem.* **52**(21), 6546–6551. 10.1021/jf049500s (2004). 15479021 10.1021/jf049500s

[37] Garst, R. Alkyl Polyglycosides—New Solutions for Agricultural Applications, Ch. 7, 131–137 https://onlinelibrary.wiley.com/doi/abs/10.1002/9783527614691.ch7 (John Wiley & Sons Ltd, 1996).

[38] Drummond, C. J., Warr, G. G., Grieser, F., Ninham, B. W. & Evans, D. F. Surface properties and micellar interfacial microenvironment of n-dodecyl.beta.-d-maltoside. *J. Phys. Chem.* **89**, 2103–2109. 10.1021/j100256a060 (1985).

[39] Niraula, B. B., Chun, T. K., Othman, H. & Misran, M. Dynamic-interfacial properties of dodecyl-beta-d-maltoside and dodecyl-beta-d-fructofuranosyl-alpha-d-glucopyranoside at dodecane/water interface. *Colloids Surf. A Physicochem. Eng. Asp.* **248**, 157–166 (2004).

[40] Balzer, D. Cloud point phenomena in the phase behavior of alkyl polyglucosides in water. *Langmuir* **9**, 3375–3384. 10.1021/la00036a009 (1993).

[41] Kutschmann, E.-M., Findenegg, G. H., Nickel, D. & von Rybinski, W. Interfacial tension of alkylglucosides in different APG/oil/water systems. *Colloid Polym. Sci.* **273**, 565–571. 10.1007/BF00658686 (1995).

[42] Iglauer, S., Wu, Y., Shuler, P., Tang, Y. & Goddard, W. A. Analysis of the influence of alkyl polyglycoside surfactant and cosolvent structure on interfacial tension in aqueous formulations versus n-octane. *Tenside Surfactants Deterg.* **47**, 87–97. 10.3139/113.110056 (2010).

[43] Schönherr, J., Baur, P. & Uhlig, B. A. Rates of cuticular penetration of 1-naphthylacetic acid (NAA) as affected by adjuvants, temperature, humidity and water quality. *Plant Growth Regul.* **31**, 61–74. 10.1023/A:1006354732358 (2000).

[44] Schreiber, L. Review of sorption and diffusion of lipophilic molecules in cuticular waxes and the effects of accelerators on solute mobilities. *J. Exp. Bot.* **57**, 2515–2523. 10.1093/jxb/erj173 (2006). 16882646 10.1093/jxb/erj173

[45] Zeisler-Diehl, V., Muller, Y. & Schreiber, L. Epicuticular wax on leaf cuticles does not establish the transpiration barrier, which is essentially formed by intracuticular wax. *J. Plant Physiol.* **227**, 66–74 (2018). 29653782 10.1016/j.jplph.2018.03.018

[46] Stock, D. & Holloway, P. J. Possible mechanisms for surfactant-induced foliar uptake of agrochemicals. *Pestic. Sci.* **38**, 165–177 (1993).

[47] Pambou, E. et al. Structural features of reconstituted cuticular wax films upon interaction with nonionic surfactant c12e6. *Langmuir* **34**, 3395–3404. 10.1021/acs.langmuir.8b00143 (2018). 29444568 10.1021/acs.langmuir.8b00143

[48] Baales, J., Zeisler-Diehl, V. V., Malkowsky, Y. & Schreiber, L. Interaction of surfactants with barley leaf surfaces: Time-dependent recovery of contact angles is due to foliar uptake of surfactants. *Planta* **255**, 1. 10.1007/s00425-021-03785-z (2021). 34837118 10.1007/s00425-021-03785-z PMC8626361

[49] Schreiber, L. & Riederer, M. Ecophysiology of cuticular transpiration: Comparative investigation of cuticular water permeability of plant species from different habitats. *Oecologia* **107**, 426–432. 10.1007/BF00333931 (1996). 28307383 10.1007/BF00333931

[50] Buchholz, A. & Schönherr, J. Thermodynamic analysis of diffusion of non-electrolytes across plant cuticles in the presence and absence of the plasticiser tributyl phosphate. *Planta* **212**, 103–111. 10.1007/s004250000372 (2000). 11219574 10.1007/s004250000372

[51] Vittal, L. V. M., Rookes, J., Boyd, B. & Cahill, D. Analysis of plant cuticles and their interactions with agrochemical surfactants using a 3D printed diffusion chamber. *Plant Methods* **19**, 37. 10.1186/s13007-023-00999-y (2023). 37005584 10.1186/s13007-023-00999-y PMC10067233

[52] Shi, T., Schönherr, J. & Schreiber, L. Accelerators increase permeability of cuticles for the lipophilic solutes metribuzin and iprovalicarb but not for hydrophilic methyl glucose. *J. Agric. Food Chem.* **53**(7), 2609–2615. 10.1021/jf048242w (2005). 15796601 10.1021/jf048242w

[53] Ma, H. et al. Dynamical adsorption behavior prediction of dried tobacco leaf heterogeneous interfaces through simulation and image recognition techniques. *Langmuir* **40**, 19195–19208. 10.1021/acs.langmuir.4c02358 (2024). 39192631 10.1021/acs.langmuir.4c02358

[54] Schönherr, J. Calcium chloride penetrates plant cuticles via aqueous pores. *Planta* **212**, 112–118. 10.1007/s004250000373 (2000). 11219575 10.1007/s004250000373

[55] Fagerstrom, A. et al. Surfactant softening of plant leaf cuticle model wax – A differential scanning calorimetry (dsc) and quartz crystal microbalance with dissipation (qcm-d) study. *J. Colloid Interface Sci.* **426**, 22–30 (2014). 24863760 10.1016/j.jcis.2014.03.064

[56] Schonherr, J. A mechanistic analysis of penetration of glyphosate salts across astomatous cuticular membranes. *Pest Manag. Sci.* **58**, 343–351 (2002). 11975182 10.1002/ps.462

[57] Baur, P. & Aponte, J. Co-penetration of Actives and Adjuvants and Its Significance for the Matched Pair Liaison, Vol. 1171 of ACS Symposium Series, 23–39 (American Chemical Society, 2014). 10.1021/bk-2014-1171.ch002.0.

[58] Uhlig, B. & Wissemeier, A. Reduction of non-ionic surfactant phytotoxicity by divalent cations. *Crop Prot.* **19**, 13–19 (2000).

[59] Devkota, P. & Johnson, W. G. Efficacy of dicamba and glyphosate as influenced by carrier water pH and hardness. *Weed Technol.* **34**, 101–106 (2020).

[60] Schortgen, G. P. & Patton, A. J. Influence of hard water on 2,4-d formulations for the control of dandelion. *Weed Technol.* **35**, 371–379 (2021).

[61] de Ruiter, H. 2,4-d salts and adjuvants: Review and perspectives. In *Pesticide Formulations and Application Systems*: 23rd Volume, (ASTM International, 2003).

[62] Barthlott, W., Mail, M., Bhushan, B. & Koch, K. Plant surfaces: Structures and functions for biomimetic innovations. *Nano-Micro Lett.* **9**, 23. 10.1007/s40820-016-0125-1 (2017). 10.1007/s40820-016-0125-1 PMC6223843 30464998

[63] BARTHLOTT, W. et al. Classification and terminology of plant epicuticular waxes. *Bot. J. Linn. Soc.* **126**, 237–260 10.1111/j.1095-8339.1998.tb02529.x (2008).

[64] BARTHLOTT, W. et al. Classification and terminology of plant epicuticular waxes. *Bot. J. Linn. Soc.* **126**, 237–260 https://www.sciencedirect.com/science/article/pii/S0024407497901376 (1998).

[65] Grunhofer, P. & Schreiber, L. Humboldt review: Cutinized and suberized barriers in leaves and roots: Similarities and differences. *J. Plant Physiol.* **282**, 153921 https://www.sciencedirect.com/science/article/pii/S0176161723000159 (2023). 10.1016/j.jplph.2023.153921 36780757

[66] Seufert, P. et al. Building a barrier: The influence of different wax fractions on the water transpiration barrier of leaf cuticles. *Front. Plant Sci.* **12**, https://www.frontiersin.org/journals/plant-science/articles/10.3389/fpls.2021.766602 (2022). 10.3389/fpls.2021.766602 PMC8766326 35069622

[67] Schönherr, J. Water permeability of isolated cuticular membranes: The effect of ph and cations on diffusion, hydrodynamic permeability and size of polar pores in the cutin matrix. *Planta* **128**, 113–126. 10.1007/BF00390312 (1976). 24430686 10.1007/BF00390312

[68] Schonherr, J. & Huber, R. Plant cuticles are polyelectrolytes with isoelectric points around three 1. *Plant Physiol.* **59**, 145–150. 10.1104/pp.59.2.145 (1977). 16659804 10.1104/pp.59.2.145 PMC542352

[69] Yamada, Y., Wittwer, S. H. & Bukovac, M. J. Penetration of ions through isolated cuticles 123. *Plant Physiol.* **39**, 28–32. 10.1104/pp.39.1.28 (1964). 16655874 10.1104/pp.39.1.28 PMC550021

[70] Yamada, Y., Rasmussen, H. P., Bukovac, M. J. & Wittwer, S. H. Binding sites for inorganic ions and urea on isolated cuticular membrane surfaces. *Am. J. Bot.* **53**, 170–172 https://bsapubs.onlinelibrary.wiley.com/doi/abs/10.1002/j.1537-2197.1966.tb07317.x (1966).

[71] Williams, D. E. & Coleman, N. T. Cation exchange properties of plant root surfaces. *Plant Soil* **2**, 243–256. 10.1007/BF01852352 (1950).

[72] Mendes de Oliveira, D. et al. Binding of divalent cations to acetate: molecular simulations guided by raman spectroscopy. *Phys. Chem. Chem. Phys.* **22**, 24014–24027 10.1039/D0CP02987D (2020). 33078182

[73] Tredenick, E. C., Farrell, T. W., Forster, W. A. & Psaltis, S. T. P. Nonlinear porous diffusion modeling of hydrophilic ionic agrochemicals in astomatous plant cuticle aqueous pores: A mechanistic approach. *Front. Plant Sci.* **8**, https://www.frontiersin.org/journals/plant-science/articles/10.3389/fpls.2017.00746 (2017). 10.3389/fpls.2017.00746 PMC5423917 28539930

[74] Tredenick, E. C., Farrell, T. W. & Forster, W. A. Mathematical modeling of diffusion of a hydrophilic ionic fertilizer in plant cuticles: Surfactant and hygroscopic effects. *Front. Plant Sci.* **9**, https://www.frontiersin.org/journals/plant-science/articles/10.3389/fpls.2018.01888 (2018). 10.3389/fpls.2018.01888 PMC6306450 30619434

[75] Tredenick, E. C. & Farquhar, G. D. Dynamics of moisture diffusion and adsorption in plant cuticles including the role of cellulose. *Nat. Commun.* **12**, 5042. 10.1038/s41467-021-25225-y (2021). 34413297 10.1038/s41467-021-25225-y PMC8377085

[76] Kobayashi, T. et al. The solvation of na+ ions by ethoxylate moieties enhances adsorption of sulfonate surfactants at the air-water interface. *J. Colloid. Interface. Sci.* **682**, 924–933 (2025). 39657414 10.1016/j.jcis.2024.11.229

[77] Baur, P. Surfactant effects on cuticular penetration of neutral polar compounds: Dependence on humidity and temperature. *J. Agric. Food Chem.* **47**, 753–761. 10.1021/jf980507h (1999). 10563965 10.1021/jf980507h

[78] Van Der Spoel, D. et al. Gromacs: Fast, flexible, and free. *J. Comput. Chem.* **26**, 1701–1718 (2005). 16211538 10.1002/jcc.20291

[79] Pronk, S. et al. Gromacs 4.5: A high-throughput and highly parallel open source molecular simulation toolkit. *Bioinformatics* **29**, 845 (2013). 23407358 10.1093/bioinformatics/btt055 PMC3605599

[80] Abraham, M. J. et al. Gromacs: High performance molecular simulations through multi-level parallelism from laptops to supercomputers. *SoftwareX* **1**, 19–25 (2015).

[81] Jorgensen, W. L., Ghahremanpour, M. M., Saar, A. & Tirado-Rives, J. Opls/2020 force field for unsaturated hydrocarbons, alcohols, and ethers. *J. Phys. Chem. B* **128**(1), 250–262. 10.1021/acs.jpcb.3c06602 (2024). 38127719 10.1021/acs.jpcb.3c06602

[82] Ghahremanpour, M. M., Tirado-Rives, J. & Jorgensen, W. L. Refinement of the optimized potentials for liquid simulations force field for thermodynamics and dynamics of liquid alkanes. *J. Phys. Chem. B* **126**, 5896–5907. 10.1021/acs.jpcb.2c03686 (2022). 35914179 10.1021/acs.jpcb.2c03686 PMC9939004

[83] Koch, K., Bhushan, B. & Barthlott, W. Diversity of structure, morphology and wetting of plant surfaces. *Soft Matter* **4**, 1943–1963. 10.1039/B804854A (2008).

[84] Koch, K. & Ensikat, H.-J. The hydrophobic coatings of plant surfaces: Epicuticular wax crystals and their morphologies, crystallinity and molecular self-assembly. *Micron* **39**, 759–772 (2008). 18187332 10.1016/j.micron.2007.11.010

[85] Ensikat, H., Boese, M., Mader, W., Barthlott, W. & Koch, K. Crystallinity of plant epicuticular waxes: Electron and x-ray diffraction studies. *Chem. Phys. Lipids.* **144**, 45–59 (2006). 16879815 10.1016/j.chemphyslip.2006.06.016

[86] van Maarseveen, C. & Jetter, R. Composition of the epicuticular and intracuticular wax layers on *Kalanchoe daigremontiana* (Hamet et Perr. de la Bathie) leaves. *Phytochemistry* **70**, 899–906 (2009). 19446855 10.1016/j.phytochem.2009.04.011

[87] Buschhaus, C., Herz, H. & Jetter, R. Chemical composition of the epicuticular and intracuticular wax layers on adaxial sides of *Rosa canina* leaves. *Ann. Bot.* **100**, 1557–1564. 10.1093/aob/mcm255 (2007). 17933845 10.1093/aob/mcm255 PMC2759234

[88] Jetter, R. & Riederer, M. Localization of the transpiration barrier in the epi- and intracuticular waxes of eight plant species: Water transport resistances are associated with fatty acyl rather than alicyclic components. *Plant Physiol.* **170**, 921–934. 10.1104/pp.15.01699 (2015). 26644508 10.1104/pp.15.01699 PMC4734581

[89] Buschhaus, C. & Jetter, R. Composition differences between epicuticular and intracuticular wax substructures: How do plants seal their epidermal surfaces?. *J. Exp. Bot.* **62**, 841–853. 10.1093/jxb/erq366 (2011). 21193581 10.1093/jxb/erq366

[90] Reynhardt, E. C. & Riederer, M. Structures and molecular dynamics of plant waxes. *Eur. Biophys. J.* **23**, 59–70. 10.1007/BF00192206 (1994).

[91] Reynhardt, E. C. The role of hydrogen bonding in the cuticular wax of *Hordeum vulgare* L. *Eur. Biophys. J.* **26**, 195–201. 10.1007/s002490050071 (1997).

[92] Reynhardt, E. C. & Riederer, M. Structure and molecular dynamics of the cuticular wax from leaves of *Citrus aurantium* L.. *J. Phys. D. Appl. Phys.* **24**, 478. 10.1088/0022-3727/24/3/036 (1991).

[93] Riederer, M. & Schneider, G. The effect of the environment on the permeability and composition of citrus leaf cuticles. *Planta* **180**, 154–165. 10.1007/BF00193990 (1990). 24201939 10.1007/BF00193990

[94] Sasani, N., Bock, P., Felhofer, M. & Gierlinger, N. Raman imaging reveals in-situ microchemistry of cuticle and epidermis of spruce needles. *Plant Methods* **17**, 17. 10.1186/s13007-021-00717-6 (2021). 33557869 10.1186/s13007-021-00717-6 PMC7871409

[95] Hama, T. et al. Probing the molecular structure and orientation of the leaf surface of *Brassica oleracea* L. by polarization modulation-infrared reflection-absorption spectroscopy. *Plant. Cell. Physiol.* **60**, 1567–1580. 10.1093/pcp/pcz063 (2019). 31020320 10.1093/pcp/pcz063

[96] Shafer, W. E. & Bukovac, M. J. Studies on octylphenoxy surfactants: III. Sorption of Triton X–100 by isolated tomato fruit cuticles. *Plant. Physiol.* **85**, 965–970 (1987). 16665839 10.1104/pp.85.4.965 PMC1054377

[97] Hall, O. Limitations of surfactant and pH effects on herbicide behavior in woody plants. *Weed. Sci.* **21**, 221–223 (1973).

[98] Radulovic, J., Sefiane, K. & Shanahan, M. E. On the effect of ph on spreading of surfactant solutions on hydrophobic surfaces. *J. Colloid Interface Sci.* **332**, 497–504 https://www.sciencedirect.com/science/article/pii/S0021979709000186 (2009). 10.1016/j.jcis.2008.12.078 19185880

[99] Matsson, M. K., Kronberg, B. & Claesson, P. M. Adsorption of alkyl polyglucosides on the solid/water interface: Equilibrium effects of alkyl chain length and head group polymerization. *Langmuir* **20**, 4051–4058. 10.1021/la035959p (2004). 15969397 10.1021/la035959p

[100] de Ruiter, H., Straatman, K. R. & Meinen, E. Influence of two fatty amine surfactants on foliar absorption, translocation, and efficacy of 2,4-d triethanolamine salt. *J. Agric. Food Chem.* **43**, 3093–3097. 10.1021/jf00060a018 (1995).

[101] de Ruiter, H., Straatman, K. & Meinen, E. The influence of a fatty amine surfactant on foliar absorption and translocation of the trolamine salt and iso-octyl ester of 2,4-d. *Pestic. Sci.* **38**, 145–154 https://onlinelibrary.wiley.com/doi/abs/10.1002/ps.2780380209 (1993).

[102] Winkler, A. & Knoche, M. Penetration of sweet cherry skin by 45ca-salts: Pathways and factors. *Sci. Rep.* **11**, 11142. 10.1038/s41598-021-90727-0 (2021). 34045647 10.1038/s41598-021-90727-0 PMC8160134

[103] Winkler, A. & Knoche, M. Calcium uptake through skins of sweet cherry fruit: Effects of different calcium salts and surfactants. *Sci. Hortic.* **276**, 109761 (2021). https://www.sciencedirect.com/science/article/pii/S0304423820305896.

[104] Knoche, M., Petracek, P. D. & Bukovac, M. J. Finite dose diffusion studies: I. characterizing cuticular penetration in a model system using naa and isolated tomato fruit cuticles. *Pest Manag. Sci.* **56**, 1005–1015 https://scijournals.onlinelibrary.wiley.com/doi/abs/10.1002/1526-4998%28200012%2956%3A12%3C1005%3A%3AAID-PS188%3E3.0.CO%3B2-Y (2000).

[105] Staples, E. et al. The influence of sorbitol on the adsorption of surfactants at the air–liquid interface. *J. Colloid Interface Sci.* **184**, 391–398 https://www.sciencedirect.com/science/article/pii/S0021979796906335 (1996). 10.1006/jcis.1996.0633 8978541

[106] Li, J., Amador, C. & Wilson, M. R. Computational predictions of interfacial tension, surface tension, and surfactant adsorption isotherms. *Phys. Chem. Chem. Phys.* **26**, 12107–12120 10.1039/D3CP06170A (2024). 38587476

[107] Shi, L. et al. Adsorption isotherms of aqueous c12e6 and cetyltrimethylammonium bromide surfactants on solid surfaces in the presence of low molecular weight coadsorbents. *Langmuir* **25**, 5536–5544. 10.1021/la8041988 (2009). 19382783 10.1021/la8041988

[108] Lu, J. R. et al. Neutron reflection from a layer of monododecyl hexaethylene glycol adsorbed at the air-liquid interface: The configuration of the ethylene glycol chain. *J. Phys. Chem.* **97**, 8012–8020. 10.1021/j100132a034 (1993).

[109] Nikas, Y. J., Puvvada, S. & Blankschtein, D. Surface tensions of aqueous nonionic surfactant mixtures. *Langmuir* **8**, 2680–2689. 10.1021/la00047a018 (1992).

[110] Sottmann, T. & Strey, R. Ultralow interfacial tensions in water–n-alkane–surfactant systems. *J. Chem. Phys.* **106**, 8606–8615. 10.1063/1.473916 (1997).

[111] Cardenas, H. et al. Determining interfacial tension and critical micelle concentrations of surfactants from atomistic molecular simulations. *J. Colloid Interface Sci.* **674**, 1071–1082 https://www.sciencedirect.com/science/article/pii/S0021979724015066 (2024). 10.1016/j.jcis.2024.07.002 39013277

[112] Lidmar, J. Improving the efficiency of extended ensemble simulations: The accelerated weight histogram method. *Phys. Rev. E* **85**, 056708 https://link.aps.org/doi/10.1103/PhysRevE.85.056708 (2012). 10.1103/PhysRevE.85.056708 23004904

[113] Lindahl, V., Lidmar, J. & Hess, B. Accelerated weight histogram method for exploring free energy landscapes. *J. Chem. Phys.* **141**, 044110. 10.1063/1.4890371 (2014). 25084884 10.1063/1.4890371

[114] Frenkel, D. & Ladd, A. J. C. New Monte Carlo method to compute the free energy of arbitrary solids. Application to the fcc and hcp phases of hard spheres. *J. Chem. Phys.* **81**, 3188–3193 10.1063/1.448024 (1984).

[115] Torrie, G. & Valleau, J. Nonphysical sampling distributions in Monte Carlo free-energy estimation: Umbrella sampling. *J. Comput. Phys.* **23**, 187–199 (1977).

[116] Sresht, V., Lewandowski, E. P., Blankschtein, D. & Jusufi, A. Combined molecular dynamics simulation–molecular-thermodynamic theory framework for predicting surface tensions. *Langmuir* **33**, 8319–8329. 10.1021/acs.langmuir.7b01073 (2017). 28749139 10.1021/acs.langmuir.7b01073

[117] Miller, R., Aksenenko, E. & Fainerman, V. Dynamic interfacial tension of surfactant solutions. *Adv. Colloid Interface Sci.* **247**, 115–129 (2017). 28063521 10.1016/j.cis.2016.12.007

[118] Qazi, M. J. et al. Dynamic surface tension of surfactants in the presence of high salt concentrations. *Langmuir* **36**, 7956–7964. 10.1021/acs.langmuir.0c01211 (2020). 32545966 10.1021/acs.langmuir.0c01211 PMC7366510

[119] Chai, J. et al. Adsorption equilibrium and dynamic surface tension of alkyl polyglucosides and their mixed surfactant systems with CTAB and SDS in the surface of aqueous solutions. *J. Mol. Liq.* **264**, 442–450 (2018).

