# Supplementary material for: Surfactants promote the transport of hydrophilic compounds through hydrophobic nanopores in leaves: mechanistic insights
Source: Sci Rep. 2026 Mar 7;16:12535. doi: 10.1038/s41598-026-41943-z (PMC13086960; doi:10.1038/s41598-026-41943-z)
Supplement: Supplementary file 1 — Supplementary Information. [file 41598_2026_41943_MOESM1_ESM.pdf]

Supplementary Information for "Surfactants Promote the Transport of Hydrophilic Compounds through Hydrophobic Nanopores in Leaves: Mechanistic Insights"

Takeshi Kobayashi[a], Alexander Moriarty[a], Kristo Kotsi[a], Teng Dong[a], Ian McRobbie[b], Panagiota Angeli[a], Alberto Striolo[c]

[a]*Department of Chemical Engineering, University College London, Torrington Place WC1E 7JE, London, United Kingdom*
[b]*Innospec Ltd, Oil Sites Road, Ellesmere Port, Cheshire CH65 4EY, UK, United Kingdom*
[c]*School of Sustainable Chemical, Biological and Materials Engineering, The University of Oklahoma, Norman, OK 73019, United States*

Contents

A Molecular Structures of the Chemical Compounds 1

B Surface Structure of the Epicuticular Wax 2

C Molecular Dynamics (MD) Simulation Details 2
C.1 General remarks . . . 2
C.2 Force field validation . . . 2
C.3 Simulation Procedure for the Adsorption/Penetration of AIs into the Pore within Epicuticular Wax . . 6

D Calculations of Free Energy 8
D.1 Thermodynamics Integration (TI) . . . 8
D.2 Accelerated Weight Histogram Method . . . 8
D.3 Umbrella sampling . . . 8

E Calculation of The Penetration Rate 10

F Antagonistic Effect due to $CaCl_2$ on MG Uptake 11
F.1 The interaction between MG and Ca^{2+} . . . 11
F.2 Aggregation of $C_{12}E_6$ Monomers in Bulk . . . 11
F.3 Adsorption free energy of $C_{12}E_6$ on the leaf surface in the $CaCl_2$ solution . . . 12
F.4 Diffusion of $C_{12}E_6$ Due to Increased Viscosity Induced by $CaCl_2$ . . . 13
F.5 Binding Free Energy of Ca^{2+} and Na^+ to COO^- . . . 14
F.6 Change in the Adsorption Energy of $C_{12}E_6$ in the Presence of Bound Ca^{2+} on the Leaf Surface 14

G Surface adsorption of AIs 17

H Molecular Distributions in Each System Not Shown in the Main Manuscript 19

A. Molecular Structures of the Chemical Compounds

The chemical structures of the accelerators, used in this SI are presented in Figure. A.1.

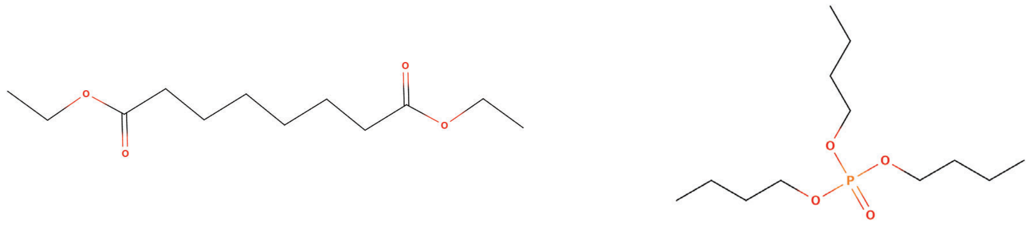

(a) DESU (b) TBP

Figure A.1: Chemical structures of (a) diethylsuberate (DESU), (b) tributyl phosphate (TBP).

B. Surface Structure of the Epicuticular Wax

In addition to the (001) surface (Figure. B.2 (a) and (b)) used in the main manuscript, other surface orientations can also be considered to account for different structural configurations. The surfaces exposing the hydrocarbon chains of the alkane to the liquid phase have two possible configurations, as illustrated in Figure. B.2 (c), (d), (e), and (f). These surfaces are defined as the (100) and (010) surfaces, respectively. While the adsorption free energy of surfactants may vary across the (001), (100), and (010) surfaces, the fundamental adsorption mechanism of the surfactants remains consistent across these orientations (results not shown).

C. Molecular Dynamics (MD) Simulation Details

C.1. General remarks

All simulations were conducted using the GROMACS 2021.5 software package [1, 2, 3]. Unless explicitly stated otherwise, the temperature in all simulations was maintained at T=300 K using a velocity-rescaling thermostat [4] with a coupling time constant of 0.1 ps. Electrostatic interactions were computed using the particle mesh Ewald (PME) method [5, 6], with a real-space cutoff of 1.2 nm, a grid spacing of 0.16 nm, and a fourth-order interpolation scheme. Lennard-Jones (LJ) interactions were truncated at a distance of 1.2 nm and shifted to zero at this cutoff and beyond. The equations of motion were integrated using the Leapfrog algorithm with a time step of 1.5 fs. A time step of 1.5 fs was chosen to ensure the stability of the simulations at high surfactant densities while maintaining the accuracy of the results. In our previous study [7], we tested a smaller time step of 0.5 fs and observed only negligible differences in the calculated surface tension. Longer timesteps were found to induce inaccuracies in the simulations. All bonds involving hydrogen atoms were constrained using the LINCS algorithm [8]. Prior to production simulations, energy minimization was performed using the conjugate-gradient method. Initial configurations for all systems were prepared using a combination of custom-developed Python scripts and the GROMACS utility ”gmx insert-molecules.” Additional system-specific details are provided in the following subsections.

C.2. Force field validation

We selected the force field for our all-atom MD simulations to ensure compatibility with the available experimental data. As our primary focus is on interfacial properties, we evaluated surface tension (ST) and interfacial tension (IFT) using various combinations of water models with the OPLS/2020 force field [9, 10], which was used to represent the surfactant and plant wax (alkane). The OPLS/2020 force field was specifically designed for alkanes, alcohols, and ethers, and it has been shown to accurately predict the density and heat of evaporation for a wide range of hydrocarbons, making it well-suited for this study. For the water models, we tested SPC/E [11], TIP4P/2005 [12], and OPC4 [13] covering three- and four-interaction-point models, which are known to provide improved agreement with experimental properties such as dielectric properties as well as ST [14, 15, 7]. In our previous study [7], these models were evaluated in the context of anionic/nonionic surfactant mixtures and demonstrated comparable accuracy in capturing the interactions of ions with non-ionic ethoxylated alcohol surfactants [7], except for the absolute values of ST and the free energy of interaction between ions and surfactants.

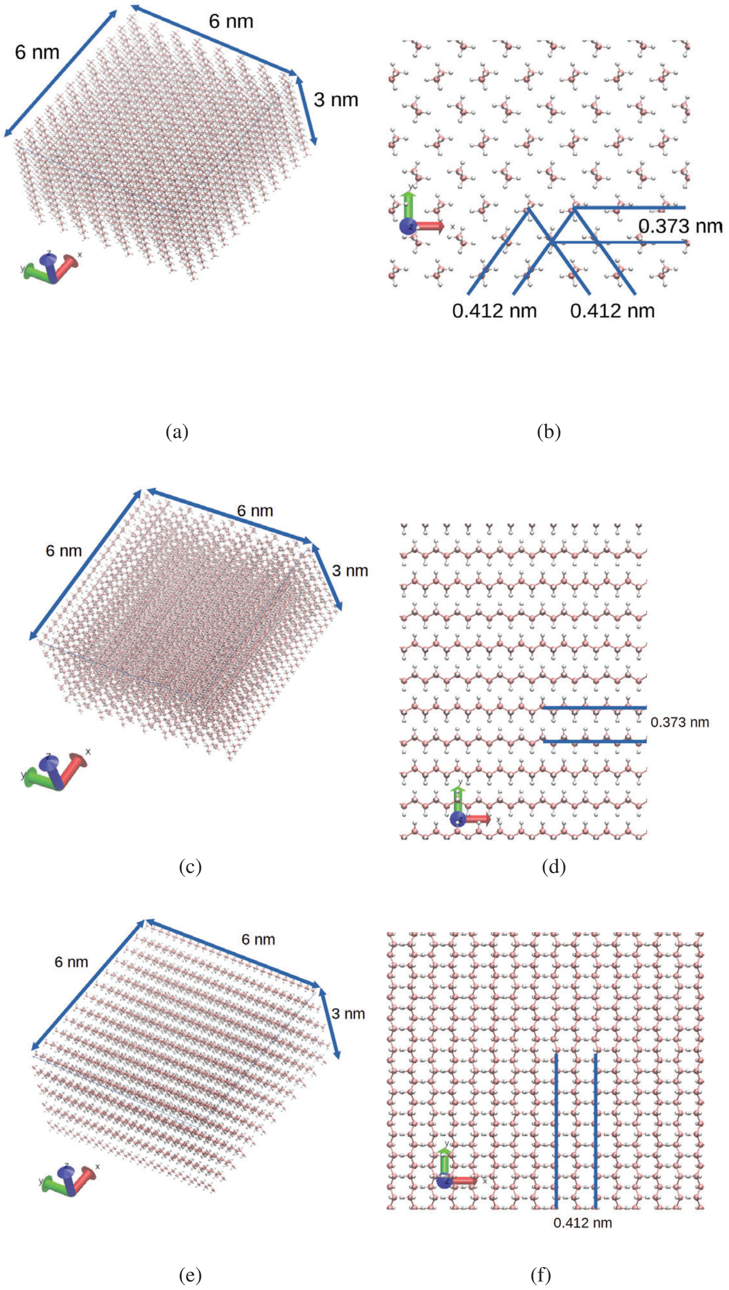

Figure B.2: The snapshots of the model leaf: (a) the unit cell of the (001) surface, (b) the top view of the (001) surface, (c) the unit cell of the (100) surface, (d) the top view of the (100) surface, (e) the unit cell of the (010) surface, and (f) the top view of the (010) surface.

For the ST calculations, initial configurations were prepared by placing surfactant molecules at the two surfaces formed at the top and bottom of an aqueous phase consisting of 10,000 water molecules in a simulation box with fixed dimensions of 6 nm × 6 nm × 50 nm. The aqueous phase extended to 50 nm in the z-direction (perpendicular to the surface) to ensure the presence of a vacuum phase (see snapshot in Figure C.4 (a)). Periodic boundary conditions were imposed along all three directions. Completely random initial configurations tended to evolve into states where surfactant molecules were adsorbed onto the surface, often resulting in an unequal number of surfactant molecules on the two surfaces. This behavior reflects the surface-active nature of the surfactant and is further supported by the free energy of adsorption of surfactants at the surface [7]. To ensure an equal number of surfactant molecules on both surfaces, we initially placed an equal number of surfactant molecules at each surface. Following energy minimization, equilibration was performed for 5 to 15 ns under constant volume and constant temperature (NVT) conditions. Given the slow dynamics of surfactant molecules at the interface, simulations at high surfactant concentrations were initially conducted at an elevated temperature (350 K) for 9 ns to accelerate equilibration, followed by 6 ns of equilibration at 300 K before initiating the production runs. Production runs were then conducted for 100 ns for ST without surfactant and 150 ns for ST with surfactant under NVT conditions.

The ST was computed as the average of the instantaneous ST values at each time step, using the following equation [16, 17]:

$$\gamma = \frac{L_z}{2}\left(P_{zz} - \frac{1}{2}\left(P_{xx} + P_{yy}\right)\right), \tag{1}$$

In Eq.(1), P_{xx}, P_{yy}, and P_{zz} are the volume averaged pressure in x, y, and z directions respectively, and L_z is the average box length in z direction. To ensure that the reported ST values represent equilibrium properties, simulations were performed at least twice, with the resulting values differing by less than 5% between runs. The ST was computed as the averaged value of the instantaneous ST over the entire trajectory using Eqn. (1) [17]. The representative snapshots are shown in Figure. C.4: (a) and (e) for $C_{12}E_6$, and (c) and (g) for $C_{12}G_1$.

For the IFT calculation, the vacuum phase in the equilibrated configurations from the ST calculations was filled with 1500 octane or decane molecules. After energy minimization, a short equilibration was performed for 1 ns under constant volume and constant temperature (NVT) conditions. Subsequently, the systems were equilibrated for 7.5 ns under constant temperature and pressure (NPT) conditions using the Berendsen barostat [18], with semi-isotropic coupling. The coupling constant was set to 1 ps, and the compressibility values were 0 /bar in the xy direction and 4.5 × 10^{-5} /bar in the z direction, under 1 bar pressure in both the xy and z directions. This procedure led to the shrinkage of the simulation box in the z direction to approximately 22–25 nm. Production runs were then conducted for 100 ns for IFT without surfactant and 150 ns for IFT with surfactant under NPT conditions using the Parrinello-Rahman barostat [19, 20], with the same coupling parameters as those used during the NPT equilibration. The representative snapshots are shown in Figure. C.4: (b) and (f) for $C_{12}E_6$, and (d) and (h) for $C_{12}G_1$.

In terms of the ST, OPC4 exhibited the closest value (70.3 mN/m) to the experimental value (72 mN/m [21]) compared to TIP4P/2005 (64.7 mN/m) and SPC/E (58.3 mN/m). However, for the water/octane and water/decane IFTs (51.16 and 51.98 mN/m [22]), TIP4P/2005 showed better agreement (54.6 and 55.6 mN/m) compared to OPC4 (57.6 and 58.6 mN/m). A comprehensive comparison of ST, the water/octane, and water/decane IFTs across the three water models is presented in Table S1. Since our primary focus is the interaction with plant wax (alkane), TIP4P/2005 was selected for this study. However, as reported in Ref. [7], different water models may not significantly influence the fundamental interactions of ionic species and non-ionic surfactants, except for the absolute values of ST or IFT, which follow the order OPC4 > TIP4P/2005 > SPC/E.

We calculated the ST and water/octane IFT for $C_{12}E_6$ at the saturated surfactant surface density at the CMC (6.7×10^{-5} mol/L [23]) at ambient temperature (~ 298 K). Although the adsorbed amount varies depending on the measurement method and the surface/interface properties [24, 25, 26, 27, 23] ($\Gamma = 3.2 \sim 3.7 \times 10^{-6}$ mol/m^2), we selected $\Gamma_{C_{12}E_6} = 3.5 \times 10^{-6}$ mol/m^2, at which the ST and water/octane IFT showed better agreement between experimental values (51.16 [22] and 3 [28] mN/m) and simulations at 298 ~ 300 K, without significant deformation of the surfactant monolayer at the interface. Similarly, we calculated ST and water/decane IFT for $C_{12}G_1$. Notably, decane was used instead of octane due to the limited availability of data for octane. The ST at CMC ($1.4 \sim 1.8 \times 10^{-4}$ M) at approximately 298 K is reported to range from 28 ~ 38 mN/m [29, 30] at a surface density of $\Gamma = 3.3 \sim 5.5 \times 10^{-6}$ mol/m^2 [29, 31]. The water/decane IFT at CMC is approximately 0.5 mN/m [31], but significant variations up to 18.29 mN/m have also been reported [29]. We selected $\Gamma_{C_{12}G_1} = 4.8 \times 10^{-6}$ mol/m^2 for $C_{12}G_1$ in the same manner as for

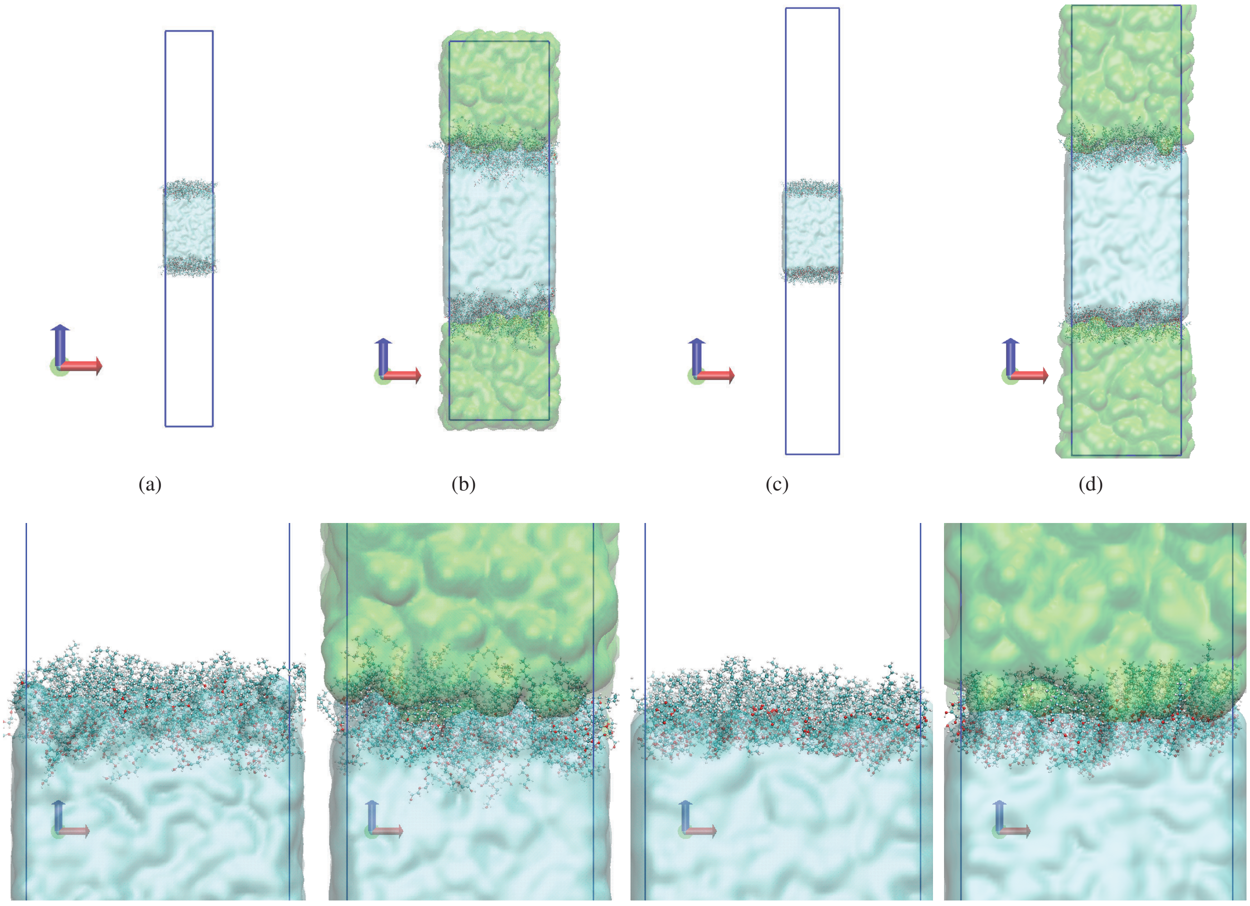

(a) (b) (c) (d)

(e) (f) (g) (h)

Figure C.3: Snapshots of the entire system at the vacuum/water interface for $C_{12}E_6$ (a) and $C_{12}G_1$ (b), and at the octane/water interface for $C_{12}E_6$ (c) and $C_{12}G_1$ (d). The bottom panels (e), (f), (g), and (h) provide close-up views of the interfacial region in the corresponding top panels (a), (b), (c), and (d), respectively.

$C_{12}E_6$. As a reference, the ST and IFT (water/decane) were also tested with $C_{12}G_2$. The reference surface density was about $\Gamma = 3.7 \times 10^{-6}$ mol/m^2 [32]. In our simulation, we selected $\Gamma_{C_{12}G_2} = 3.8 \times 10^{-6}$ mol/m^2, where we obtained similar ST and IFT (water/decane) values to experimental results of 35 ~ 36 mN/m for ST [33, 34, 30], and 3 ~ 5 mN/m for IFT [33, 34, 30]. Please note that alkylpolyglycosides (APGs) are generally a mixture of different degrees of polymerization, such as $C_{12}G_{1.3}$, and in some cases, they exhibit lower surface tension (ST) (29 mN/m) compared to the isomers $C_{12}G_1$ and $C_{12}G_2$ [32]. We tested a mixture of $C_{12}G_1$ and $C_{12}G_2$ to achieve an average degree of polymerization of 1.3 ($C_{12}G_{1.3}$), specifically a molar ratio of $C_{12}G_1$:$C_{12}G_2$ = 7:3. However, at the calculated ST value corresponding to the surface density determined by the same molar ratio ($\Gamma_{C_{12}G_{1.3}} = 0.7\Gamma_{C_{12}G_1} + 0.3\Gamma_{C_{12}G_2} = 4.5 \times 10^{-6}$ mol/m^2), no decrease in ST was observed (40 mN/m). Therefore, in our study, we use only the individual isomers, assuming that the improved ST for the mixture can be attributed to the higher packing density of the surfactants at the surface.

The adsorption amount of surfactant on the solid surface may not differ significantly from that on the air/water surface. As a general trend, smaller head groups with lower glycoside polymerization result in higher surfactant densities at the interface [32]. All the reference values for SF and IFT at CMC, as well as the simulated values, are summarized in Table S1.

	air/water	water/octane	water/decane	$C_{12}E_6$		$C_{12}G_1$		$C_{12}G_2$	
	ST	IFT	IFT	ST	IFT	ST	IFT	ST	IFT
Experiment	72^{*a}	51.16^{*b}	51.98^{*b}	32 ~ 33^{*c}	3^{*d}	28 ~ 38^{*e}	0.5^{*f}	35 ~ 36^{*h}	3~ 5^{*i}
Simulation									
(SPC/E)	58.3 ± 0.2^{*g}	46.6 ± 0.2	47.2 ± 0.3	-	-	-	-	-	-
(TIP4P/2005)	64.7 ± 0.1^{*g}	54.6 ± 0.3	55.6 ± 0.4	31.0 ± 0.7	1.5 ± 1.3	37.3 ± 0.9	3.7 ± 1.5	37.2 ± 2.7	2.3 ± 2.4
(OPC4)	70.3 ± 0.2^{*g}	57.6 ± 0.3	58.6 ± 0.4	-	-	-	-	-	-

Table S1: Experimental vs simulated values of ST and IFT (mN/m) at CMC. The IFT is calculated at water/octane interface for $C_{12}E_6$, and at water/decane interface for $C_{12}G_1$. The surface surfactant density for the simulations are $\Gamma = 3.5 \times 10^{-6}$ mol/m^2 for $C_{12}E_6$, $\Gamma = 4.8 \times 10^{-6}$ mol/m^2 for $C_{12}G_1$, and $\Gamma = 3.8 \times 10^{-6}$ mol/m^2 for $C_{12}G_2$.
^{*a} From Ref. [21]. ^{*b} From Ref. [22]. ^{*c} From Ref. [27, 35]. ^{*d} From Ref. [28]. ^{*e} From Ref. [29, 30]. ^{*f} From Ref. [31, 36]. ^{*g} From Ref. [7]. ^{*h} From Ref. [33, 34, 30]. ^{*i} From Ref. [36].

MG showed a slight decrease in surface tension (ST) at 2.2 mol/L to 63.4 ± 0.3 mN/m, compared with pure water (64.7 ± 0.1 mN/m). Sugars such as glucose are known to increase ST [37], whereas other studies report an ST decrease for dextran [38]. Thus, the surface activity of MG remains controversial. Nevertheless, we can reasonably assume that any surface activity of MG is very weak. For our purposes, its hydrophilic nature arising from –OH groups and its weak surface activity are important for its solvation in water and weak adsorption on the model leaf.

Regarding the force field for inorganic ions (Ca^{2+}, Na^+, and Cl^-), we utilize the Madrid 2019 model [39] with a charge rescaling factor of 0.85. This model, being compatible with the TIP4P/2005 water model, accurately predicts the viscosity and density of various electrolyte solutions, including ternary mixtures. Furthermore, the salts do not precipitate at concentrations close to the experimentally observed solubility limits in water, which are 81.3 g/100 g (7.3 mol/L) for $CaCl_2$ and 36.0 g/100 g (6.2 mol/L) for NaCl [40, 41]. Since conventional force fields suffer from salt precipitation at significantly lower concentrations, this model is well-suited for the high-concentration $CaCl_2$ and NaCl solution used in this study.

C.3. Simulation Procedure for the Adsorption/Penetration of AIs into the Pore within Epicuticular Wax

The penetration simulations were conducted in three steps. First, a solution containing AIs and surfactants/accelerators was prepared in the vacuum phase. Then, a 10 ns equilibration simulation was performed, following the same procedure as described in the previous ST and IFT calculations. For certain systems, an additional 150 ns of simulations were conducted to evaluate bulk properties or ST.

Second, the equilibrated system in the vacuum phase was placed on top of the flat epicuticular wax without a pore with a thickness of 3 nm. After energy minimization, the system was then equilibrated through a 50 ns simulation under NVT condition, with an elevated temperature of 320 K applied from 10 ns to 30 ns to facilitate equilibration. An additional 100 ns of simulation at 300 K under NVT was performed to collect bulk properties and generate different initial configurations for the subsequent penetration simulations. During the simulations, the atoms in the leaf wax were fixed. The removal of this constraint did not result in significant differences in the molecular distribution within

the solution. Additionally, no dissolution of wax molecules into the solution was observed. A periodic boundary condition was imposed in the xy-direction, parallel to the leaf surface, creating an infinite slab representation of the leaf surface with the solution on top. In the z-direction, perpendicular to the surface, two Lennard-Jones walls were placed at z = 0 nm and z = 50 nm. The bottom wall at z = 0 nm consists of pseudo-carbon atoms, while the top wall at z = 30 nm consists of pseudo-repulsive carbon atoms, both with a density of 41 nm^{-3}. These walls interact with surrounding molecules via a 9-3 Lennard-Jones potential, mimicking the interactions of a bulk alkane phase beneath the bottom wall and a short-range repulsive force at the top. Additionally, three pseudo-empty layers were introduced behind the walls to eliminate unphysical electrostatic interactions between periodic images while maintaining the effect of the bulk wax layer below the bottom wall and preventing molecules from escaping the system to the top.

Finally, at least five configurations of the solution were extracted from the 100 ns simulations on the leaf surface at 0, 20, 40, 60, and 80 ns. If necessary, additional configurations were extracted at different time points to confirm that no $CaCl_2$ uptake occurs, and to confirm the solvation of Na^+ and MG into $C_{12}E_6$ by running several short simulations. These independent configurations were then placed on the leaf surface containing a pore with a thickness of 15 nm. After energy minimization, simulations were conducted for 200 ns under NVT condition to monitor the penetration of molecules into the pore. Since the solution configurations were already equilibrated on the leaf surface, the initial configurations accurately represent the state of the system at the onset of penetration. The same Lennard-Jones walls were applied at z = 0 nm and z = 50 nm for consistency with the previously described setup. When molecules reached the bottom boundary (z = 0 nm), penetration ceased due to the presence of the wall.

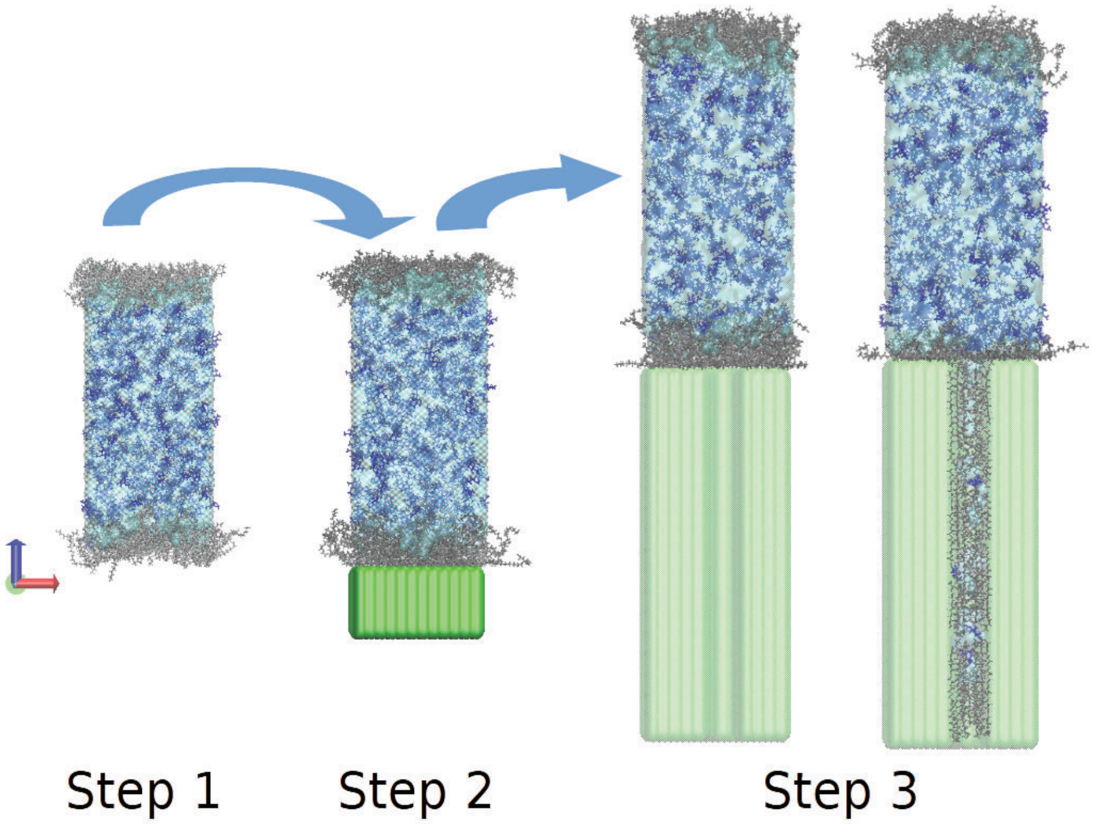

Figure C.4: The schematic explanation of the process of the penetration simulation. The example of the MG penetration with $C_{12}E_6$.

D. Calculations of Free Energy

D.1. Thermodynamics Integration (TI)

The solvation free energy (ΔG) is calculated using thermodynamic integration (TI)[42]. In this method, a target molecule is selected as a probe, and its partial charges (Coulomb interactions) and LJ interactions are gradually turned off in a procedure referred as 'alchemical transformation'. A coupling parameter, $\lambda \in$ [[0, 0], [0.05, 0], [0.1, 0], [0.15, 0], [0.2, 0], [0.25, 0], [0.30, 0], [0.35, 0], [0.4, 0], [0.45, 0], [0.5, 0], [0.55, 0], [0.6, 0], [0.65, 0], [0.7, 0], [0.75, 0], [0.8, 0], [0.85, 0], [0.9, 0], [0.95, 0], [1, 0], [1, 0.05], [1, 0.1], [1, 0.15], [1, 0.2], [1, 0.25], [1, 0.3], [1, 0.35], [1, 0.4], [1, 0.45], [1, 0.5], [1, 0.55], [1, 0.6], [1, 0.65], [1, 0.7], [1, 0.75], [1, 0.8], [1, 0.85], [1, 0.9], [1, 0.95], [1, 1]] is employed to progressively switch the interactions in the Hamiltonian U(λ) from a fully interacting ($\lambda = 0$) to a non-interacting ($\lambda = 1$) between a probe surfactant molecule and the other molecules in the system. The first and the second numbers in each set [$\lambda^{\text{elec}}, \lambda^{\text{LJ}}$] correspond to the coupling parameter for the electrostatic interaction and the Lennard-Jones interaction, respectively. Thermodynamic Integration was performed by first linearly turning off the electrostatic interactions λ^{elec} from 0 to 1, while maintaining full LJ interactions $\lambda^{\text{LJ}} = 0$. Then, the LJ interactions were gradually turned off using the soft-core LJ potentials [43] implemented in GROMACS to avoid singularities at the vanishing LJ potential ($\lambda = 1$) while the electrostatic interaction is kept off ($\lambda^{\text{elec}} = 1$). Accordingly, the "creating" free energy of a target molecule in solvent (ex . octane, octanol, water) is then computed using the following equation:

$$\Delta G = -\left(\int_0^1 \left\langle \frac{\partial U(\lambda^{\text{elec}})}{\partial \lambda} \right\rangle d\lambda^{\text{elec}} \Bigg|_{\lambda^{\text{LJ}}=0} + \int_0^1 \left\langle \frac{\partial U(\lambda^{\text{LJ}})}{\partial \lambda} \right\rangle d\lambda^{\text{LJ}} \Bigg|_{\lambda^{\text{elec}}=1} \right) \quad (2)$$

$$= \Delta G^{\text{elec}} + \Delta G^{\text{LJ}} \quad (3)$$

The first term corresponds to the electrostatic contribution ΔG^{elec}, and the second term corresponds to ΔG^{LJ}. Simulations at each λ were run for 5 ns, of which equilibration was run for 1 ns under the NVT ensemble as described above. The derivative $\frac{\partial U(\lambda)}{\partial \lambda}$ was computed every 1 ps.

D.2. Accelerated Weight Histogram Method

The accelerated weight histogram (AWH) method [44, 45] implemented in GROMACS is used to calculate the potential of mean force (PMF) for adsorption, single-surfactant penetration, and two-surfactant penetration free energies. In this approach, the PMF is updated by applying bias potentials on the fly during the simulation based on the histogram of sampled reaction coordinates until convergence is achieved. Consequently, it is not necessary to predefine sampling windows or sampling lengths. This feature is particularly advantageous for two-surfactant penetration, in which it is challenging to optimize appropriate grid points and sampling effort for each point along the reaction coordinates.

AWH biasing was enabled using a single bias potential in a one- or two-dimensional formulation. The biasing weights were updated every 5000 samples, providing a balance between rapid initial adaptation and stable convergence of the free-energy estimate. The AWH bias was applied along one or two reaction coordinates corresponding to the centers of mass of the surfactant molecules, each sampled over the interval from 0 nm to 4 nm for adsorption and from −4 nm to 0 nm for single-surfactant penetration, and −3 nm to 0 nm for two-surfactant penetration. Harmonic restraining potentials with a force constant of 2000 kJ mol^{-1} nm^{-2} were applied in both dimensions to confine sampling within the defined ranges while allowing efficient exploration. The diffusion parameter was set to 5×10^{-3} for both dimensions, controlling the expected mobility along the reaction coordinates and influencing the rate of bias adaptation. The initial free-energy uncertainty was set to 5 kJ mol^{-1}, allowing rapid initial exploration while avoiding overly aggressive bias updates. For initial configurations, surfactant molecules were located randomly at the different position within the interval of reaction coordinates.

D.3. Umbrella sampling

While the above mentioned TI methods are advantages for the calculating the difference between different environment such as solvent, alternative approaches can be implemented to calculate transfer free energies with respect to a specific coordinate, called reaction coordinate. One common algorithm is umbrella-sampling [46] [47], which however presents certain disadvantages. For instance, pulling the test surfactant out of a deformable assembly can

lead to local distortions, as observed in Ref. [48]. Because TI does not follow a physical path (cartecian coordinate) during the integration (i.e, see Eq. (3)), it can be used for complex systems, including highly concentrated surface assemblies. On the contrary, the umbrella sampling algorithm has been implemented here to quantify the free energy of adsorption for molecules as a function of the coordinate perpendicular to the surface. This method is used for the free energy calculations with one reaction coordinate throughout this SI. Umbrella sampling, coupled with the weighted histogram analysis method (WHAM) [49] is used to enhance the sampling along the reaction coordinates. In our umbrella sampling analysis, the reaction coordinate ξ is the position of the center of mass of surfactant along the axis (z axis) perpendicular to the leaf surface. For the calculation of free energy of adsorption, one target molecule is restrained by a harmonic potential of 2000 kJ·mol^{-1}·nm^{-1} at a certain reaction coordinate ξ, which is equally separated by $\approx$ 0.05 nm. The total length extends to 3 to 4 nm, corresponding to 60 to 80 different simulations (windows) along the reaction coordinate. 2000 kJ is chosen to efficiently sample the high free energy gradient region near the leaf wax. The initial configurations of each simulation are generated by pulling a molecule along the reaction coordinate slowly (1 nm/ns) from the already equilibrated system. Then a further 1 ns of equilibration is performed under NVT conditions, and a further 5 ns of production run is performed under NVT condition for the input of WHAM analysis. This generates enough sampling data for each window to overlap with the neighboring ones, thus generating a reliable PMF. The error of the free energy profile was estimated by the bootstrap technique [50] (the number of bootstraps = 3) implemented in the GROMACS package.

E. Calculation of The Penetration Rate

As mentioned in the manuscript, the rate constant A is calculated by fitting the data through the following equation:

$$z(t) = \frac{M_0}{B}(1 - e^{-At}) \tag{4}$$

M_0 is the applied amount of AI, and B is a coefficient representing the quantity of AI penetrating together with the surfactants. The plots of $z(t)$ for different systems are shown in Figure. E.5. Because of the limited size of the simulation domain, the maximum penetration length is constrained to 15 nm.

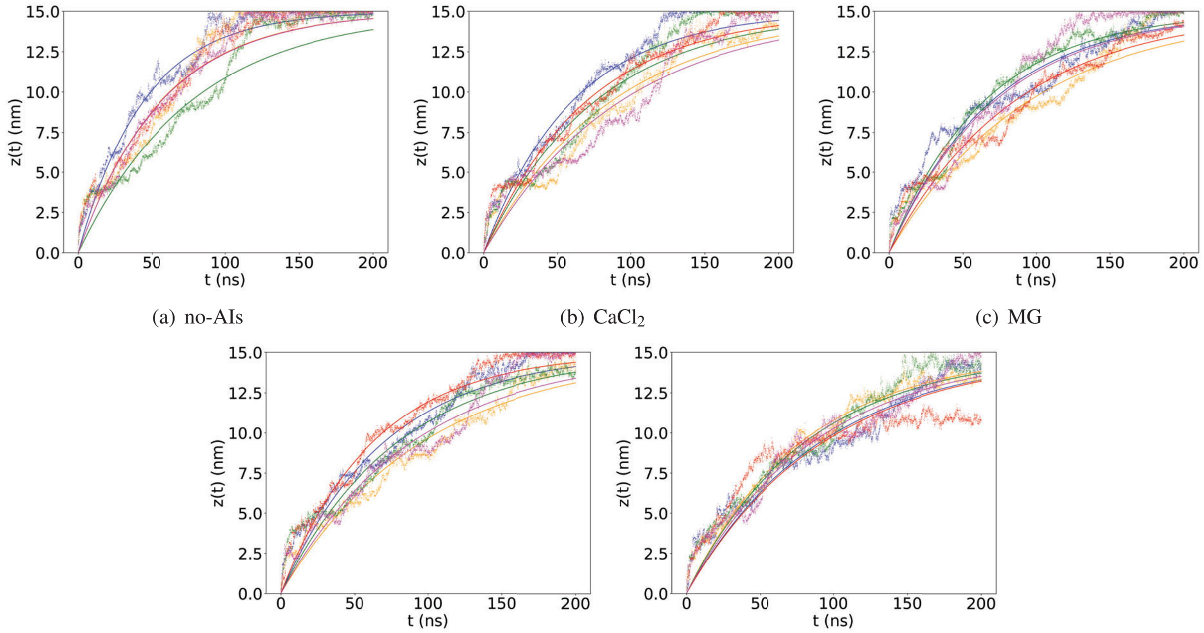

(a) no-AIs (b) $CaCl_2$ (c) MG

(d) MG + $CaCl_2$ (e) $NaCl_2$

Figure E.5: The position of the tip of $C_{12}E_6$ within the pore in wax as a function of time in each system studied. The surface of wax is located at z = 0 nm and the positive value of z corresponds toward the inside of the pore. Different colors in each figure represent five independent simulations. The points represent the original data, while the lines shows the Gaussian fitted profile for the same color of data.

F. Antagonistic Effect due to $CaCl_2$ on MG Uptake

To complement the results presented in the main manuscript, the following aspects are examined:

1. Interaction between MG and Ca^{2+}.
2. Aggregation of $C_{12}E_6$ monomers in bulk solution.
3. Change in adsorption free energy of $C_{12}E_6$ on the leaf surface in the presence of $CaCl_2$.
4. Slower diffusion of $C_{12}E_6$ due to the increased viscosity by $CaCl_2$.
5. Stronger binding of Ca^{2+} to COO^- compared to Na^+.
6. Change of the adsorption free energy of $C_{12}E_6$ on the surface with COO^- and COOH.

F.1. The interaction between MG and Ca^{2+}

Since $CaCl_2$ alone does not penetrate the nanopores in the wax layer, as shown in the main manuscript, a strong interaction between MG and Ca^{2+} may hinder the penetration of MG into the pores. To investigate this possibility, we compare the solvation energy of MG in water and in a $CaCl_2$ solution (1.1 mol/L) using the TI method described above. The difference is negligible (ΔG_{CaCl_2} - ΔG_{water} = 0.6 ± 0.8 kT). Therefore, it can be concluded that the interaction between MG and Ca^{2+} is not the primary cause of the antagonistic effect.

F.2. Aggregation of $C_{12}E_6$ Monomers in Bulk

Since larger aggregations, which lead to an increased hydrodynamic radius, result in slower aggregation or micelle dynamics, we computed the aggregation free energy of $C_{12}E_6$ monomers using the umbrella sampling method described above. The reaction coordinate ξ is defined as the distance between the centers of mass of $C_{12}E_6$ as depicted in Figure. F.6 (a). Furthermore, to examine the aggregation free energy of third molecule, the free energy is calculated as a function of the distance between the dimer and the monomer as depicted in Figure. F.6 (b). The distance between the monomers within the dimer is constrained at 0.75 nm which corresponds to the minima of PMF for the dimer formation. If aggregation plays a significant role, a similar antagonistic effect should also occur with monovalent cations such as Na^+. To investigate this, we compared three cases: pure water, a $CaCl_2$ solution (1.1 mol/L), and an NaCl solution (2.2 mol/L). The corresponding free energy profiles are shown in Figure. F.6 (c), (d), and (e). Slightly more negative values are observed for the $CaCl_2$ solution in (d) (-0.5 kT) and the NaCl solution in (e) (-2 kT) for dimer formation (orange lines at ξ = 0.75 nm) compared to the pure water case (~ 0 kT) in (c), indicating that aggregation occurs more readily in the presence of $CaCl_2$ or NaCl. For trimer formation, all three cases exhibit more negative values than for dimer formation, with a more pronounced effect in the $CaCl_2$ and NaCl solutions (-5 kT) compared to pure water (-2.5 kT). A comparison between the $CaCl_2$ and NaCl solutions reveals that $C_{12}E_6$ molecules are more likely to aggregate in the NaCl solution, leading to a "salting out" effect. In Ref. [51, 52], the authors attribute the "salting in" effect to the formation of a complex between the cation and the ethylene oxide (EO) chains in alkyl ethoxylates (AEs). However, in our study, while Na^+ ions form a complex with $C_{12}E_6$, Ca^{2+} does not, as shown in Figure. F.6 (f) and (g). The binding free energy calculated using the umbrella sampling method also indicates the preferential solvation of Na^+ ions into the EO chain of $C_{12}E_6$ at a distance of ξ = 0.25 nm. Here, the reaction coordinate ξ is defined as the distance between the center of mass of $C_{12}E_6$ and either Ca^{2+} or Na^+. Furthermore, the comparison of the solvation free energy of $C_{12}E_6$ monomer into $CaCl_2$ solution and NaCl solution using the TI methods showed a slightly unfavorable solvation of $C_{12}E_6$ into $CaCl_2$ compared to pure water (ΔG_{CaCl_2} - ΔG_{water} = 1.8 ± 0.3 kT), while slightly less unfavorable solvation into NaCl solution (ΔG_{NaCl} - ΔG_{water} = 0.4 ± 0.3 kT) was observed. This finding is consistent with previous studies. For instance, polyethylene glycol (PEG) with a long EO chain and a molecular weight of 6000–7500 preferentially binds cations in the order of $Cs^+ > Na^+ >> Li^+ \approx Mg^{2+}$ [53]. Ref. [54] reports that Ba^{2+} exhibits stronger interactions with PEG in ethanol compared to K^+, which is consistent with the findings of Ref. [55], where among divalent cations, only Ba^{2+} demonstrated significant binding to PEG.

Based on these results, the increased aggregation or "salting in" effect of $CaCl_2$ may not be directly related to cation-surfactant complex formation and is unlikely to be a primary factor contributing to the antagonistic effect of hard water on the adsorption of $C_{12}E_6$.

F.3. *Adsorption free energy of $C_{12}E_6$ on the leaf surface in the $CaCl_2$ solution*

Although the analysis above is based on small aggregates of up to three monomers, experimental evidence suggests that NaCl induces ”salting out” of AEs with long ethylene oxide (EO) chains (> 8), whereas Ca^{2+} tends to ”salt in” AEs [51, 56]. The addition of a ”salting out” salt, such as NaCl, increases the air/water surface adsorption of $C_{12}E_8$, whereas a ”salting in” salt, such as NaSCN, reduces adsorption [56]. At first glance, this may explain the antagonistic effect of $CaCl_2$, as it is a ”salting in” salt that may reduce the adsorption of $C_{12}E_6$. However, as noted in Ref. [56], the ”salting in” effect depends on the length of the EO chain in AE-type surfactants. In the presence of the ”salting in” salt NaSCN, the adsorption of $C_{12}E_{12}$ increased, exhibiting behavior similar to that observed with NaCl. Indeed, the calculation of the adsorption free energy of $C_{12}E_6$ on the leaf surface in the $CaCl_2$ solution using the umbrella sampling methods revealed a slightly enhanced adsorption of $C_{12}E_6$ in presence of $CaCl_2$ compared to pure water by -5 kT as shown in Figure. F.6 (i). Secondly, if ”salting out” were the dominant factor, synergism rather than antagonism would be expected for ”salting out” salts such as NaCl or KCl. However, KCl also exhibits antagonistic effects on AE adsorption, albeit weaker than $CaCl_2$ [57]. Finally, during evaporation, the surfactant concentration exceeds the CMC leading to the saturated adsorption of AEs on the leaf surface. Consequently, changes in AE adsorption on the leaf surface due to aggregation behavior in bulk may not be directly relevant.

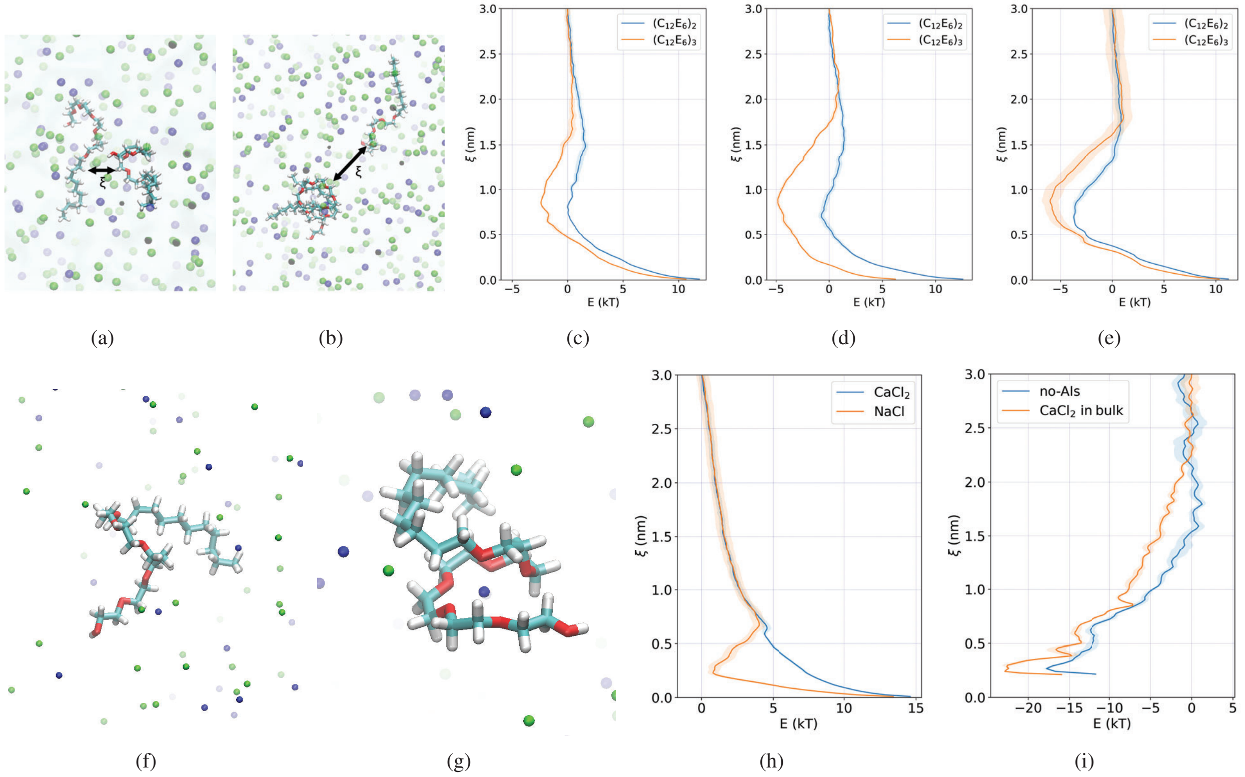

Figure F.6: (a), (b) Schematic illustration of the reaction coordinate ξ used to calculate the aggregation free energy of $C_{12}E_6$ monomers. (a) Formation of a dimer. (b) Formation of a trimer. Both (a) and (b) correspond to $CaCl_2$ solutions. The blue and green spheres represent Ca^{2+} and Cl^- ions, respectively. (c) Aggregation free energy profiles of $C_{12}E_6$ monomers in pure water. (d) Aggregation free energy profiles of $C_{12}E_6$ monomers in a 1.1 mol/L $CaCl_2$ solution. (e) Aggregation free energy profiles of $C_{12}E_6$ monomers in a 2.2 mol/L NaCl solution. The blue lines correspond to dimer formation, while the orange lines correspond to trimer formation.
(f) Snapshot of a $C_{12}E_6$ monomer in a 1.1 mol/L $CaCl_2$ solution. The blue and green spheres represent Ca^{2+} and Cl^- ions, respectively. (g) Snapshot of a $C_{12}E_6$ monomer forming a complex with Na^+ ion in a 2.2 mol/L NaCl solution. The blue and green spheres represent Na^+ and Cl^- ions, respectively. (h) Binding free energy of Ca^{2+} and Na^+ ions with $C_{12}E_6$ in water. Cl^- ions are present to neutralize the positive charge in the system. (i) Adsorption free energy of $C_{12}E_6$ on the leaf surface in 1.1 mol/L $CaCl_2$ solution. The reaction coordinate ξ is set as a distance from the leaf surface to the center of mass of $C_{12}E_6$. For the fee energy calculation the reference energy (E = 0 kT) is taken at ξ = 3 nm.

F.4. Diffusion of $C_{12}E_6$ Due to Increased Viscosity Induced by $CaCl_2$

To determine whether the primary factor contributing to the slow penetration of $C_{12}E_6$ is the reduced diffusion rate of the surfactant in the aqueous phase, we compare the diffusion constants of $C_{12}E_6$ monomers in water and in solutions of $CaCl_2$ (1.1 mol/L), NaCl (3.3 mol/L), and MG (2.2 mol/L). To prevent the aggregation of $C_{12}E_6$ in the aqueous phase, only single surfactant molecules were solvated into the solution, and the diffusion of these individual molecules was monitored for 100 ns. Assuming Brownian motion, the probability distribution of the displacement over a time interval Δt, denoted as $P(\mathbf{r}, \Delta t)$, is related to the diffusion constant D by the following equation [58]:

$$P(\mathbf{r}, \Delta t) = \frac{1}{(4\pi D\Delta t)^{2/3}} \exp\left(-\frac{|\mathbf{r}|^2}{4D\Delta t}\right) \tag{5}$$

where $\mathbf{r}$ represents the three-dimensional displacement of the molecule over the time interval Δt, and D is the diffusion constant. This relationship is generally valid for $\Delta t \gg \frac{m}{6\pi a\eta}$, where a is the radius of the molecule (assuming a spherical shape) and η denotes the viscosity of the solution. By integrating Eq. (7) over the angles θ and ϕ in spherical coordinates, we obtain the Maxwell-Boltzmann distribution of displacement as a function of the radial distance $|\mathbf{r}|$:

$$P(|\mathbf{r}|, \Delta t) = \int\int \frac{4\pi}{(4\pi D\Delta t)^{2/3}} \exp\left(-\frac{|\mathbf{r}|^2}{4D\Delta t}\right) d\theta d\phi \tag{6}$$

$$= \frac{4\pi|\mathbf{r}|^2}{(4\pi D\Delta t)^{2/3}} \exp\left(-\frac{|\mathbf{r}|^2}{4D\Delta t}\right) \tag{7}$$

To ensure the accuracy of diffusion constant calculations using these equations, Δt must be sufficiently large. In our simulations, we selected $\Delta t = 200$ ps to achieve adequate statistical reliability. Nevertheless, a qualitative comparison can be made by examining the profile of $P(r, \Delta t)$ across different solutions. The corresponding results are presented in Figure. F.7.

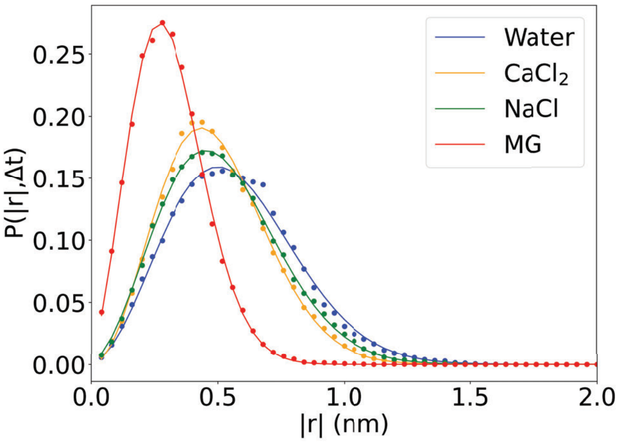

Figure F.7: Probability distribution of the displacement of $C_{12}E_6$ monomers in water and solutions of $CaCl_2$ (1.1 mol/L), NaCl (3.3 mol/L), and MG (2.2 mol/L) with $\Delta t = 200$ ps. Data points represent simulation results, while the lines correspond to fits using Eq. (7).

The estimated diffusion constants for $C_{12}E_6$ in water, $CaCl_2$, NaCl, and MG are 3.4, 2.4, 2.9, and 1.1×10^{-6} cm^2/s, respectively. If diffusion were the primary factor governing the slow adsorption of $C_{12}E_6$, the MG solution should exhibit the slowest adsorption. However, experimental evidence indicates that MG has a negligible effect on the adsorption of AEs [59, 60, 61]. Therefore, we conclude that reduced diffusion is not the dominant factor responsible for the slow adsorption of AEs in $CaCl_2$ solutions.

F.5. Binding Free Energy of Ca^{2+} and Na^{+} to COO^{-}

To demonstrate the stronger binding affinity of Ca^{2+} compared to monovalent cations (e.g., Na^{+}) on the leaf surface [62, 63], we analyzed the difference in binding free energy between Ca^{2+} and Na^{+} to COO^{-} groups located on the leaf surface, as illustrated in Figure. F.8 (a). The reaction coordinate ξ is defined as the distance between the COO^{-} group and the cation. The corresponding free energy profiles are presented in Figure. F.8 (b). While Na^{+} exhibits weak binding (compared to a separation distance of $\xi = 2.75$ nm), Ca^{2+} demonstrates significantly stronger binding with a free energy decrease of -4 kT. This result clearly indicates that Ca^{2+} binds more strongly to the leaf surface in the presence of COO^{-} compared to Na^{+}.

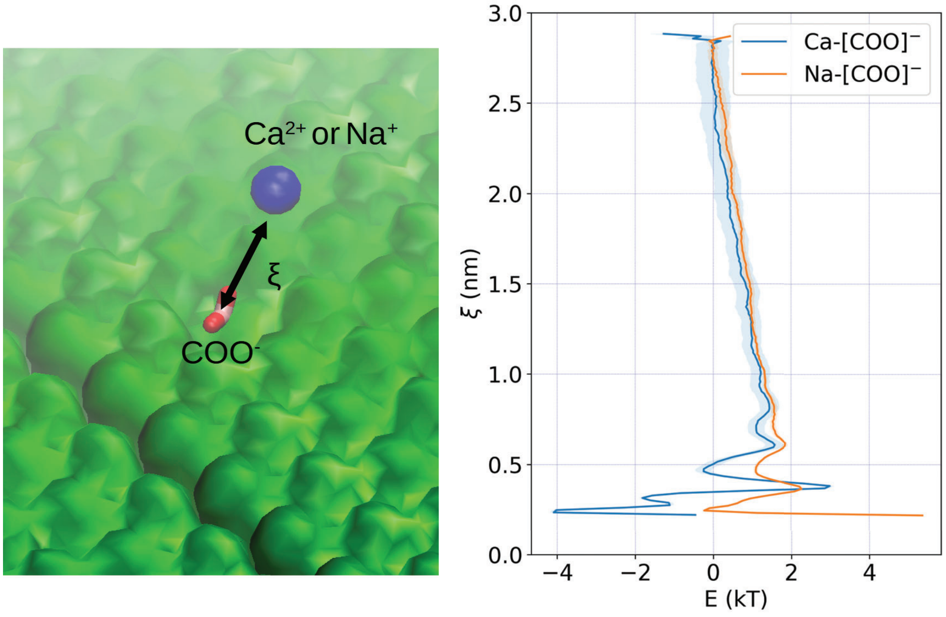

(a) (b)

Figure F.8: (a) Schematic illustration of the reaction coordinate ξ for the calculation of binding free energy between COO^{-} and Ca^{2+} or Na^{+}. The COO^{-} group is attached to the leaf surface (green). In the case of Ca^{2+}, an additional COO^{-} group is present on the leaf surface at a sufficiently large distance to prevent interaction. (b) Free energy profiles as a function of the reaction coordinate ξ for Ca^{2+} and Na^{+} binding to COO^{-}.

F.6. Change in the Adsorption Energy of $C_{12}E_{6}$ in the Presence of Bound Ca^{2+} on the Leaf Surface

To confirm the desorption or detachment of the adsorbed $C_{12}E_{6}$ monolayer from the leaf surface in the presence of bound Ca^{2+}, we calculated the adsorption free energy in systems where Ca^{2+} is bound to unesterified COO^{-} groups. For this analysis, a single $C_{12}E_{6}$ molecule was solvated in a $CaCl_2$ solution (1.1 mol/L). To examine the influence of COO^{-} groups, a relatively high density (3% of the total carbon atoms) of COO^{-} groups was attached to both the top and bottom surfaces of the leaf. To maintain charge neutrality, an equivalent number of Cl^{-} ions were removed from the solution. Additionally, to confirm that COOH groups have no significant effect on the adsorption of $C_{12}E_{6}$, we conducted a control study using a surface with the same number of COOH groups. The reaction coordinate ξ was defined as the distance from the leaf surface to the center of mass of the $C_{12}E_{6}$ molecule. Snapshots of the systems examined are presented in Figure. F.9 (a), (b), and (c). In the presence of COO^{-} groups, Ca^{2+} ions bind to the surface regardless of the specific COO^{-} locations (panels (a) and (b)), due to electrostatic interactions between Ca^{2+} and COO^{-}. Conversely, in the presence of COOH groups, Ca^{2+} ions do not bind to the surface (panel (c)). In all cases, Cl^{-} ions do not exhibit surface binding.

The corresponding adsorption free energy profiles are shown in Figure. F.9 (d), including a reference profile for a surface without COO^{-} or COOH groups (red line). Regardless of the specific COO^{-} location (blue and orange lines), the free energy increases near the surface compared to the bulk region ($\xi = 3$ nm), indicating a preference for

desorption of $C_{12}E_6$. In contrast, in the presence of COOH groups, no noticeable increase in free energy is observed near the surface compared to the clean surface (red line), suggesting favorable adsorption of $C_{12}E_6$. These findings support the experimentally observed weak adsorption of $C_{12}E_6$ in the presence of $CaCl_2$, as discussed in the main manuscript.

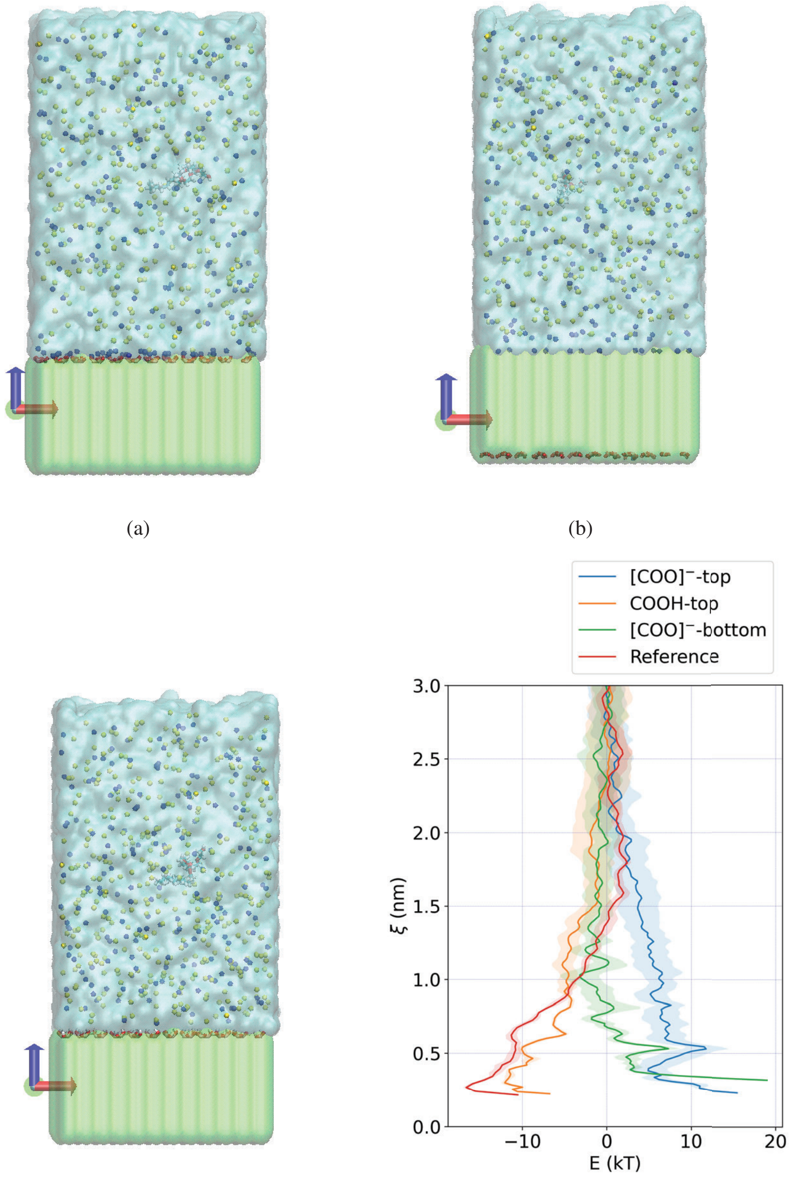

Figure F.9: (a) Snapshot illustrating the system with COO^- groups (red) attached to the top leaf surface (green) and the binding of Ca^{2+} ions (blue). Yellow spheres represent Cl^- ions. (b) Same as (a), but with COO^- groups attached to the bottom leaf surface. (c) Same as (a), but with COOH groups replacing COO^-. (d) Adsorption free energy profiles of $C_{12}E_6$ as a function of the reaction coordinate ξ, defined as the distance from the leaf surface to the center of mass of $C_{12}E_6$. The red line represents the reference case without functional groups on the surface.

G. Surface adsorption of AIs

To analyze the surface adsorption of MG, the adsorption free energies was calculated using the umbrella sampling method. The resulting free energy profiles are shown in Figure.G.10(a). It is evident that MG exhibits negligible surface adsorption ($\sim 0\ k_{\mathrm{B}}T$).

In the presence of $C_{12}E_6$, MG (2.2 mol/L) does not adsorb onto the leaf surface due to the significantly stronger surface activity of $C_{12}E_6$ ($\sim -17.5\ k\mathrm{B}T$). As shown in the average density profiles along the z-direction (perpendicular to the surface) in Figure. G.10(c), MG shows a higher density near the surface in the absence of $C_{12}E_6$, reflecting its weak surface affinity observed at the minima in the adsorption free energy in Figure.G.10(a). However, in the presence of $C_{12}E_6$, this surface adsorption disappears due to competitive exclusion by the surfactant. In contrast, $CaCl_2$ solution (1.1 mol/L) shows that both Ca^{2+} and Cl^- ions are repelled from the surface or remain solvated in the bulk aqueous phase, indicating weak or negligible surface activity.

Solvation of AIs into $C_{12}E_6$ may influence their ability to penetrate. Except for certain cations such as Na^+ which forms cation-EO complex, most ions are not favorably solvated in $C_{12}E_6$, as shown in the binding free energy profiles in Figure.G.10(b). These calculations were performed in the same manner as described in Figure.F.6. As the ether oxygen atoms in the EO groups preferentially bind cations, anions such as Cl^- exhibit repulsive interactions with $C_{12}E_6$. Neutral specie like MG is also repelled, due to its preferential solvation into aqueous phase (see at $\xi = 0.25$ nm and $\xi = 3.0$ nm). Consequently, in the presence of a saturated $C_{12}E_6$ monolayer at the interface, the adsorption of these species is suppressed due to steric and electrostatic repulsion.

Interestingly, Na^+, which exhibits strong binding to $C_{12}E_6$, penetrates the nanopore even though it is not surface active. This contrasts with $CaCl_2$, where neither Ca^{2+} nor Cl^- penetrate, not due to surface activity, but due to unfavorable solvation into $C_{12}E_6$. Cl^-, in particular, exhibits both unfavorable surface adsorption and solvation, thereby hindering the penetration of co-solvated ionic species via strong electrostatic interactions.

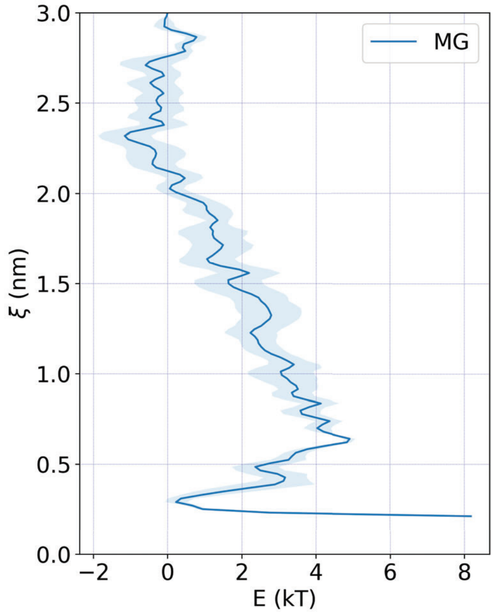

(a)

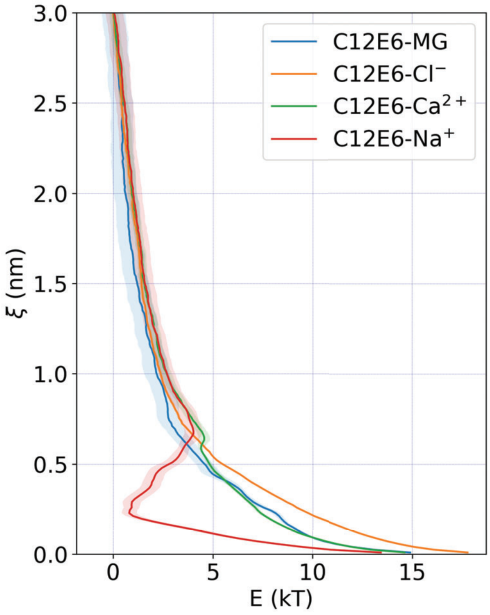

(b)

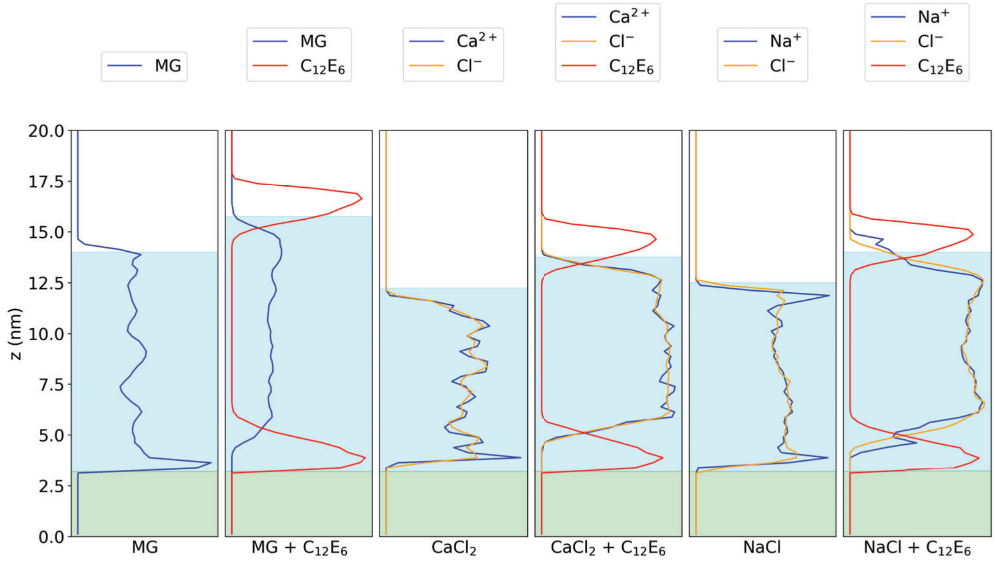

(c)

Figure G.10: (a) Adsorption free energy profiles of MG on the leaf surface as a function of the reaction coordinate ξ, defined as the distance from the leaf surface to the center of mass of the solute. (b) Binding free energy profiles of various ions to $C_{12}E_6$ in aqueous solution, plotted as a function of the reaction coordinate ξ, defined as the distance between the center of mass of the ion and that of $C_{12}E_6$. Cl^- ions are included to maintain charge neutrality. (c) Relative density profiles of species in solutions containing MG, MG + $C_{12}E_6$, $CaCl_2$, $CaCl_2$ + $C_{12}E_6$, NaCl, and NaCl + $C_{12}E_6$. The concentrations are 2.2 mol/L for MG and NaCl, and 1.1 mol/L for $CaCl_2$. The surface density of $C_{12}E_6$ is 3.5×10^{-6} mol/m^2 at both the air/water and leaf/water interfaces. The green-shaded region denotes the leaf wax layer, and the cyan-shaded region indicates the aqueous phase.

H. Molecular Distributions in Each System Not Shown in the Main Manuscript

Here, we present the spatial distribution of molecules in the systems discussed in the main manuscript, as well as additional auxiliary systems that support our observations. The examined systems are listed in Table S2. In these auxiliary systems, DESU and TBP are not treated as surfactants but are instead added to the bulk solution alongside the AIs.

System name	Explanation
$CaCl_2$	$CaCl_2$ (1.1 mol/L) with $C_{12}E_6$ ($\Gamma = 3.5 \times 10^{-6}$ mol/m^2).
Concentrated $CaCl_2$	High concentration of $CaCl_2$ (11 mol/L) with $C_{12}E_6$ ($\Gamma = 3.5 \times 10^{-6}$ mol/m^2).
MG + $CaCl_2$	Mixture of MG (2.2 mol/L) and $CaCl_2$ (1.1 mol/L) with $C_{12}E_6$ ($\Gamma = 3.5 \times 10^{-6}$ mol/m^2).
$C_{12}E_8$	$C_{12}E_8$ ($\Gamma = 3.0 \times 10^{-6}$ mol/m^2).
MG + $C_{12}E_8$	MG (2.2 mol/L) with $C_{12}E_8$ ($\Gamma = 3.0 \times 10^{-6}$ mol/m^2).
MG/DESU	Mixture of MG (2.2 mol/L) and DESU (1.1 mol/L).
MG/TBP	Mixture of MG (2.2 mol/L) and TBP (1.1 mol/L).
Hydrophilic pore	Penetration of $C_{12}G_1$ ($\Gamma = 4.8 \times 10^{-6}$ mol/m^2) into hydrophilic pore.
$C_{12}G_1/C_{12}G_2$	Penetration of $C_{12}G_1$ ($\Gamma = 3.4 \times 10^{-6}$ mol/m^2) and $C_{12}G_2$ ($\Gamma = 1.4 \times 10^{-6}$ mol/m^2) mixture.

Table S2: The list of systems examined. Below the dashed line is the auxiliary systems.

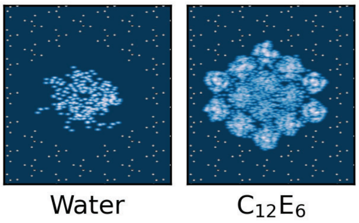

(a)

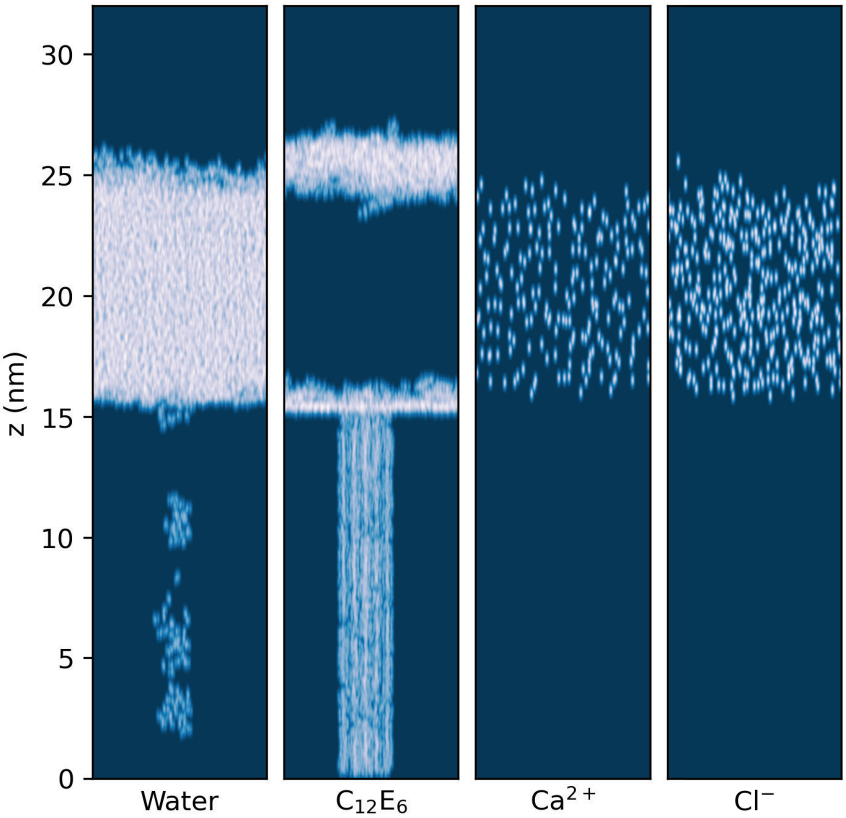

(b)

Figure H.11: Molecular distributions in $CaCl_2$ after 200 ns of the penetration simulation, as listed in Table S2. (a) Top view within the pore. (b) Side view, including the bulk phase above the leaf surface.

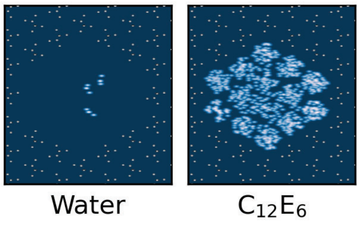

(a)

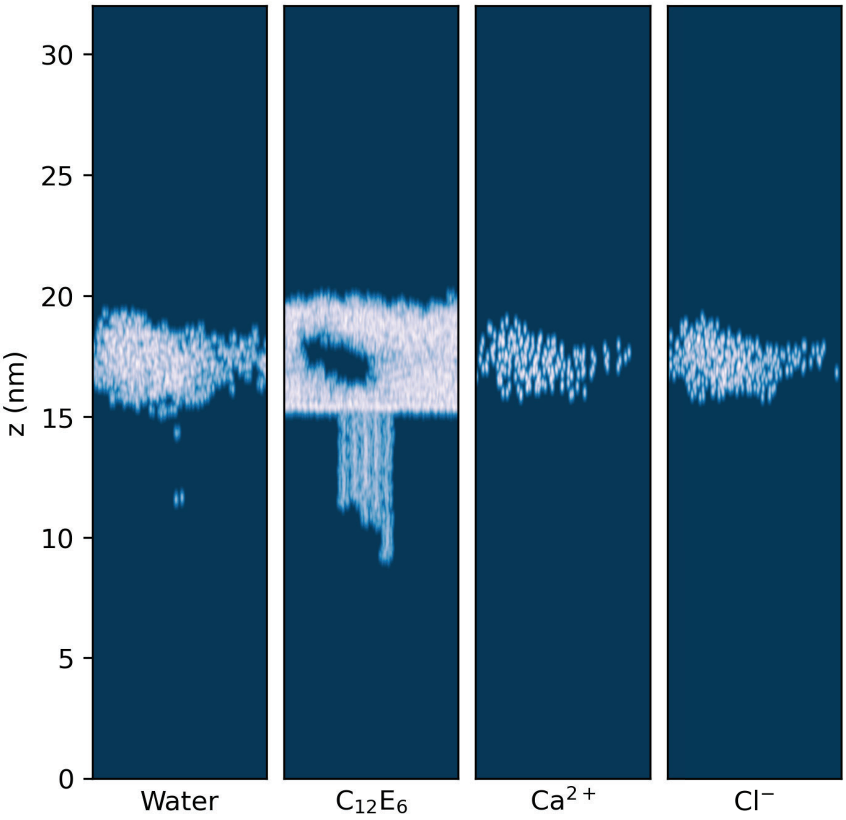

(b)

Figure H.12: Molecular distributions in concentrated $CaCl_2$ after 200 ns of the penetration simulation, as listed in Table S2. (a) Top view within the pore. (b) Side view, including the bulk phase above the leaf surface.

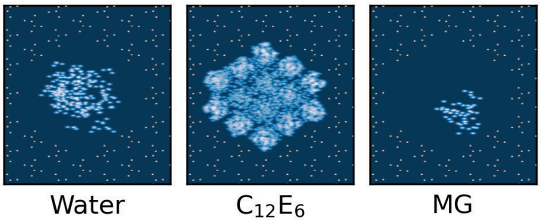

(a)

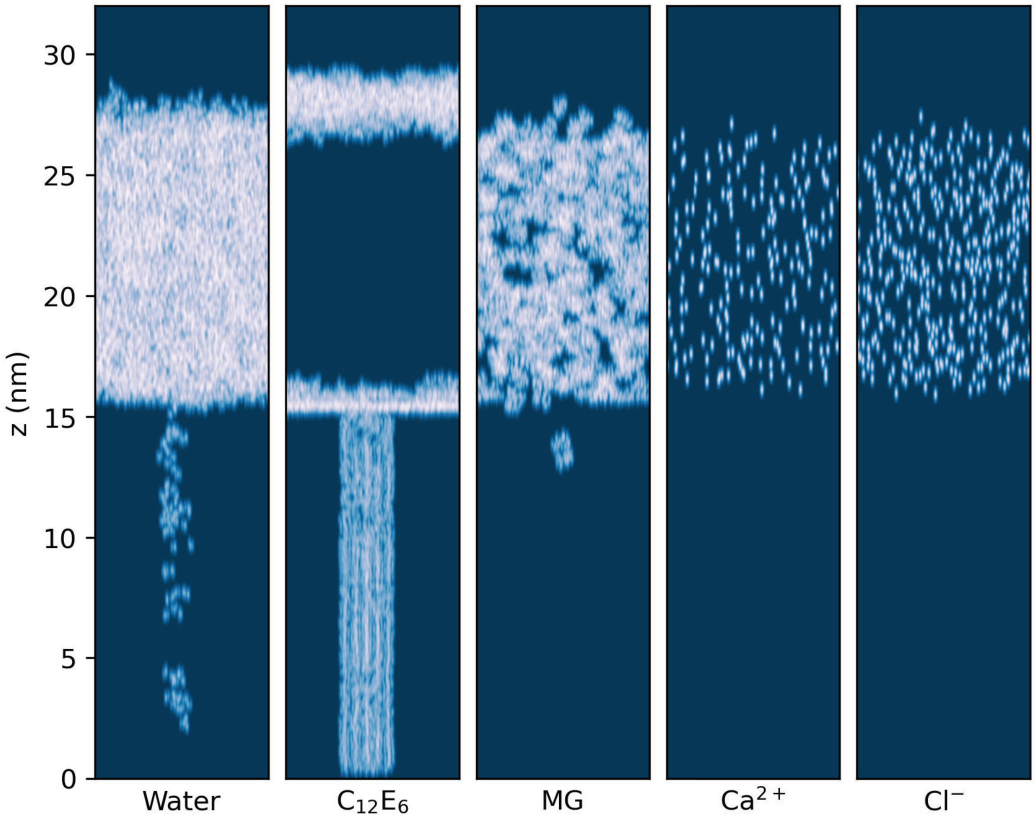

(b)

Figure H.13: Molecular distributions in concentrated MG + $CaCl_2$ after 200 ns of the penetration simulation, as listed in Table S2. (a) Top view within the pore. (b) Side view, including the bulk phase above the leaf surface.

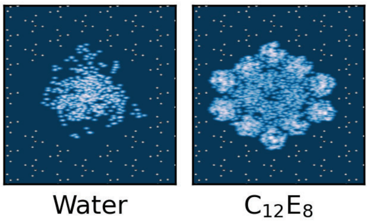

(a)

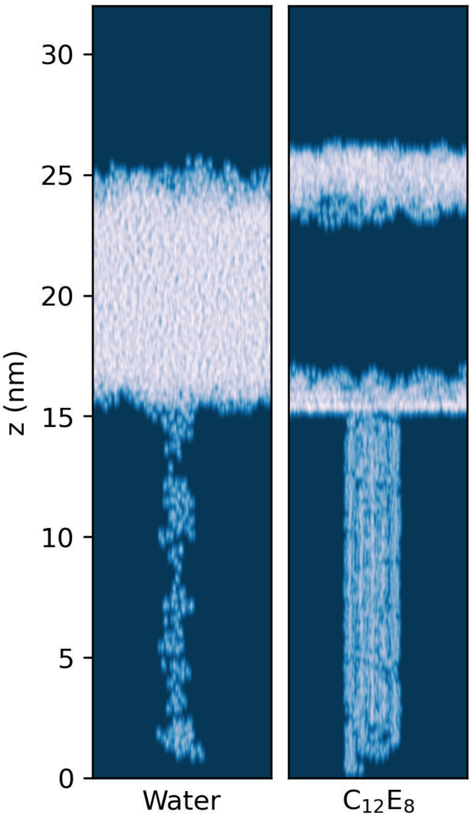

(b)

Figure H.14: Molecular distributions in C12E8 after 200 ns of the penetration simulation, as listed in Table S2. (a) Top view within the pore. (b) Side view, including the bulk phase above the leaf surface.

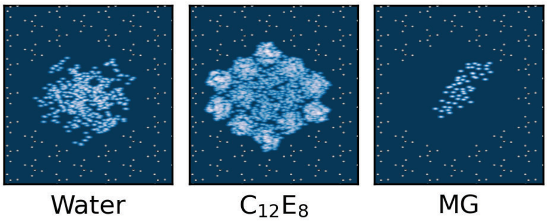

(a)

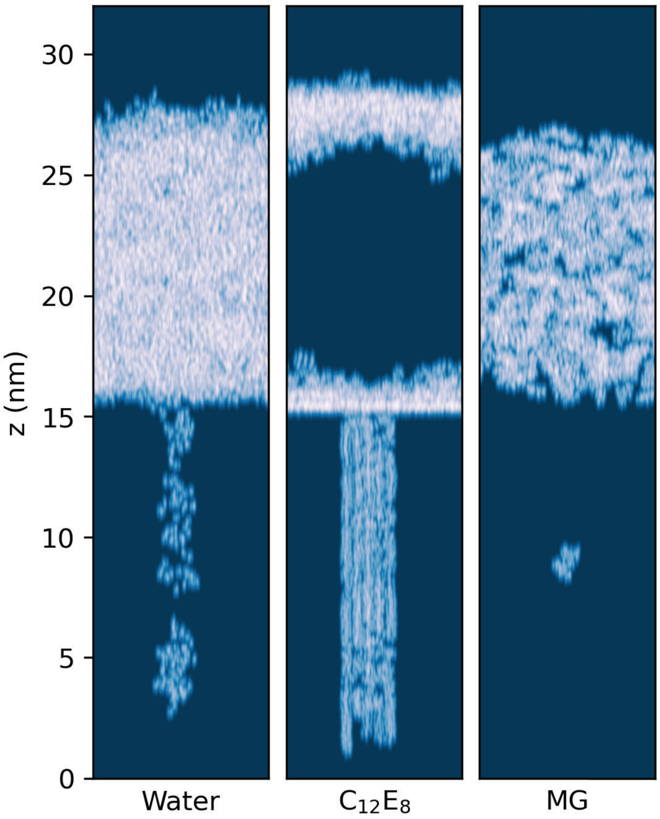

(b)

Figure H.15: Molecular distributions in MG + C12E8 after 200 ns of the penetration simulation, as listed in Table S2. (a) Top view within the pore. (b) Side view, including the bulk phase above the leaf surface.

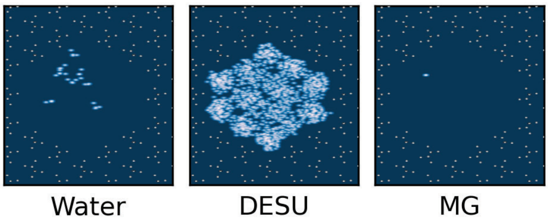

(a)

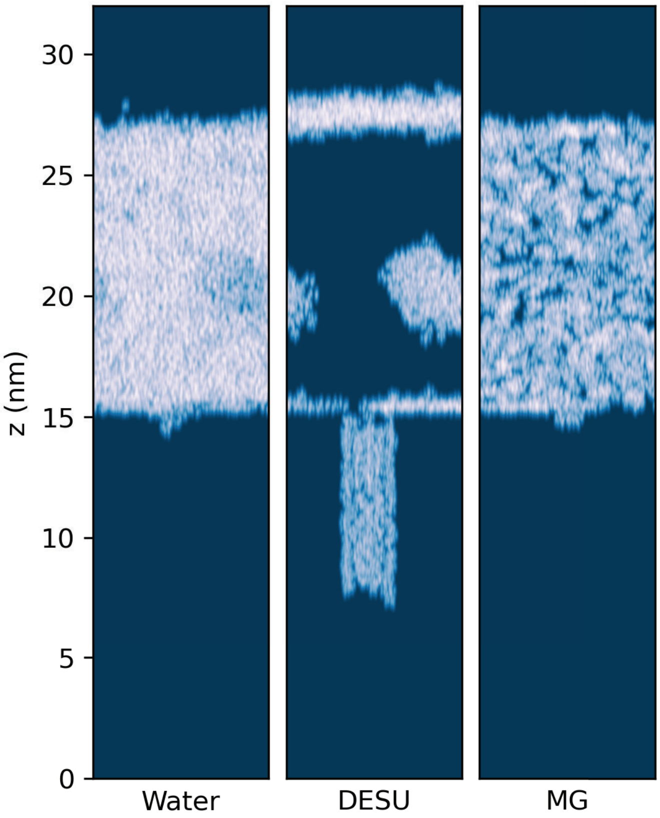

(b)

Figure H.16: Molecular distributions in MG/DESU after 200 ns of the penetration simulation, as listed in Table S2. (a) Top view within the pore. (b) Side view, including the bulk phase above the leaf surface.

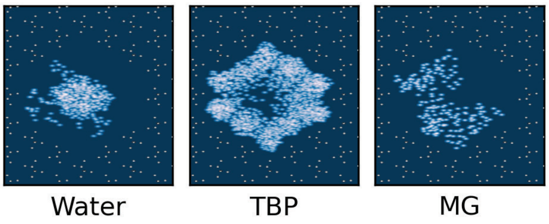

(a)

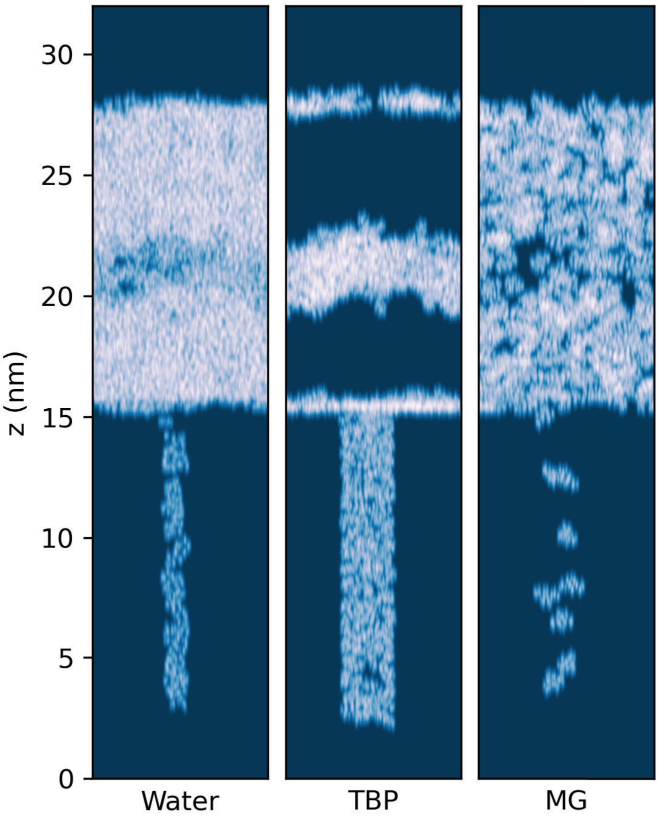

(b)

Figure H.17: Molecular distributions in MG/TBP after 200 ns of the penetration simulation, as listed in Table S2. (a) Top view within the pore. (b) Side view, including the bulk phase above the leaf surface.

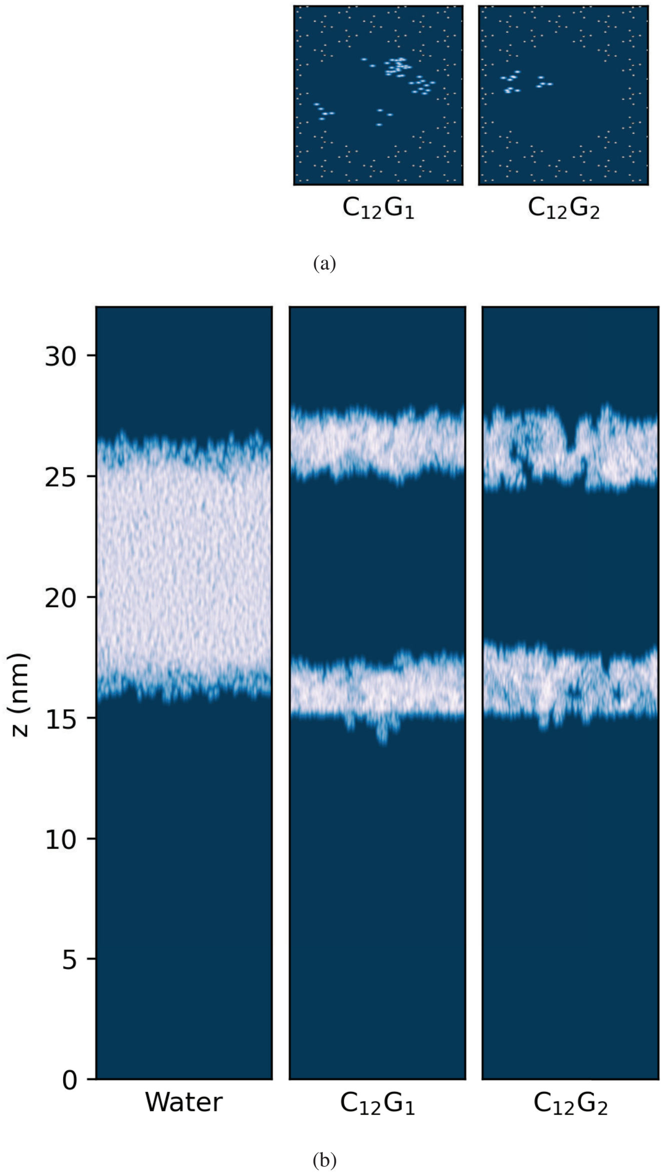

Figure H.18: Molecular distributions in $C_{12}G_1$/$C_{12}G_2$ after 200 ns of the penetration simulation, as listed in Table S2. (a) Top view within the pore. (b) Side view, including the bulk phase above the leaf surface.

References

[1] D. Van Der Spoel, E. Lindahl, B. Hess, G. Groenhof, A. E. Mark, H. J. Berendsen, Gromacs: fast, flexible, and free, J. Comput. Chem. 26 (16) (2005) 1701–1718.

[2] S. Pronk, S. Pall, R. Schulz, P. Larsson, P. Bjelkmar, R. Apostolov, M. R. Shirts, J. C. Smith, P. M. Kasson, D. van der Spoel, B. Hess, E. Lindahl, Gromacs 4.5: a high-throughput and highly parallel open source molecular simulation toolkit, Bioinformatics 29 (7) (2013) 845.

[3] M. J. Abraham, T. Murtola, R. Schulz, S. Páll, J. C. Smith, B. Hess, E. Lindahl, Gromacs: High performance molecular simulations through multi-level parallelism from laptops to supercomputers, SoftwareX 1 (2015) 19–25.

[4] G. Bussi, D. Donadio, M. Parrinello, Canonical sampling through velocity rescaling, J. Chem. Phys. 126 (1) (2007) 014101.

[5] T. Darden, D. York, L. Pedersen, Particle mesh ewald: An n log (n) method for ewald sums in large systems, J. Chem. Phys. 98 (12) (1993) 10089–10092.

[6] U. Essmann, L. Perera, M. L. Berkowitz, T. Darden, H. Lee, L. G. Pedersen, A smooth particle mesh ewald method, J. Chem. Phys. 103 (19) (1995) 8577–8593.

[7] T. Kobayashi, K. Kotsi, T. Dong, I. McRobbie, A. Moriarty, P. Angeli, A. Striolo, The solvation of na+ ions by ethoxylate moieties enhances adsorption of sulfonate surfactants at the air-water interface, Journal of Colloid and Interface Science 682 (2025) 924–933. doi:https://doi.org/10.1016/j.jcis.2024.11.229.
URL `https://www.sciencedirect.com/science/article/pii/S0021979724028170`

[8] B. Hess, H. Bekker, H. J. C. Berendsen, J. G. E. M. Fraaije, LINCS: A linear constraint solver for molecular simulations, J. Comput. Chem. 18 (1997) 1463–1472.

[9] W. L. Jorgensen, M. M. Ghahremanpour, A. Saar, J. Tirado-Rives, Opls/2020 force field for unsaturated hydrocarbons, alcohols, and ethers, The Journal of Physical Chemistry B 128 (1) (2024) 250–262, pMID: 38127719. arXiv:https://doi.org/10.1021/acs.jpcb.3c06602, doi:10.1021/acs.jpcb.3c06602.
URL `https://doi.org/10.1021/acs.jpcb.3c06602`

[10] M. M. Ghahremanpour, J. Tirado-Rives, W. L. Jorgensen, Refinement of the optimized potentials for liquid simulations force field for thermodynamics and dynamics of liquid alkanes, The Journal of Physical Chemistry B 126 (31) (2022) 5896–5907, pMID: 35914179. arXiv:https://doi.org/10.1021/acs.jpcb.2c03686, doi:10.1021/acs.jpcb.2c03686.
URL `https://doi.org/10.1021/acs.jpcb.2c03686`

[11] H. J. C. Berendsen, J. R. Grigera, T. P. Straatsma, The missing term in effective pair potentials, The Journal of Physical Chemistry 91 (24) (1987) 6269–6271. arXiv:https://doi.org/10.1021/j100308a038, doi:10.1021/j100308a038.
URL `https://doi.org/10.1021/j100308a038`

[12] J. L. F. Abascal, C. Vega, A general purpose model for the condensed phases of water: TIP4P/2005, The Journal of Chemical Physics 123 (23) (2005) 234505. arXiv:https://pubs.aip.org/aip/jcp/article-pdf/doi/10.1063/1.2121687/15377081/234505_1_online.pdf, doi:10.1063/1.2121687.
URL `https://doi.org/10.1063/1.2121687`

[13] S. Izadi, R. Anandakrishnan, A. V. Onufriev, Building water models: A different approach, The Journal of Physical Chemistry Letters 5 (21) (2014) 3863–3871, pMID: 25400877. arXiv:https://doi.org/10.1021/jz501780a, doi:10.1021/jz501780a.
URL `https://doi.org/10.1021/jz501780a`

[14] C. Vega, E. de Miguel, Surface tension of the most popular models of water by using the test-area simulation method, The Journal of Chemical Physics 126 (15) (2007) 154707. arXiv:https://doi.org/10.1063/1.2715577, doi:10.1063/1.2715577.
URL `https://doi.org/10.1063/1.2715577`

[15] L. F. Sedano, S. Blazquez, C. Vega, Accuracy limit of non-polarizable four-point water models: TIP4P/2005 vs OPC. Should water models reproduce the experimental dielectric constant?, The Journal of Chemical Physics 161 (4) (2024) 044505. arXiv:https://pubs.aip.org/aip/jcp/article-pdf/doi/10.1063/5.0211871/20072867/044505_1_5.0211871.pdf, doi:10.1063/5.0211871.
URL `https://doi.org/10.1063/5.0211871`

[16] J. G. Kirkwood, F. P. Buff, The statistical mechanical theory of surface tension, The Journal of Chemical Physics 17 (3) (1949) 338–343. arXiv:https://doi.org/10.1063/1.1747248, doi:10.1063/1.1747248.
URL `https://doi.org/10.1063/1.1747248`

[17] Gromacs 2021.5 manual.
URL `https://doi.org/10.5281/zenodo.5849961`

[18] H. J. C. Berendsen, J. P. M. Postma, W. F. van Gunsteren, A. DiNola, J. R. Haak, Molecular dynamics with coupling to an external bath, The Journal of Chemical Physics 81 (8) (1984) 3684–3690. arXiv:https://pubs.aip.org/aip/jcp/article-pdf/81/8/3684/18950084/3684_1_online.pdf, doi:10.1063/1.448118.
URL `https://doi.org/10.1063/1.448118`

[19] M. Parrinello, A. Rahman, Polymorphic transitions in single crystals: A new molecular dynamics method, Journal of Applied Physics 52 (12) (1981) 7182–7190. arXiv:https://pubs.aip.org/aip/jap/article-pdf/52/12/7182/18394754/7182_1_online.pdf, doi:10.1063/1.328693.
URL `https://doi.org/10.1063/1.328693`

[20] S. Nose, M. Klein, Constant pressure molecular dynamics for molecular systems, Molecular Physics 50 (5) (1983) 1055–1076. arXiv:https://doi.org/10.1080/00268978300102851, doi:10.1080/00268978300102851.
URL `https://doi.org/10.1080/00268978300102851`

[21] K. Kotsi, T. Dong, T. Kobayashi, I. Mc Robbie, A. Striolo, P. Angeli, Synergistic effects between a non-ionic and an anionic surfactant on the micellization process and the adsorption at liquid/air surfaces, Soft Matter (2024) –doi:10.1039/D3SM01454A.
URL `http://dx.doi.org/10.1039/D3SM01454A`

[22] S. Zeppieri, J. Rodriguez, A. L. Lopez de Ramos, Interfacial tension of alkane + water systems, Journal of Chemical Engineering Data 46 (5) (2001) 1086–1088. arXiv:https://doi.org/10.1021/je000245r, doi:10.1021/je000245r.
URL `https://doi.org/10.1021/je000245r`

[23] E. Pambou, X. Hu, Z. Li, M. Campana, A. Hughes, P. Li, J. R. P. Webster, G. Bell, J. R. Lu, Structural features of reconstituted cuticular wax films upon interaction with nonionic surfactant c12e6, Langmuir 34 (11) (2018) 3395–3404, pMID: 29444568. arXiv:https://doi.org/10.1021/acs.langmuir.8b00143, doi:10.1021/acs.langmuir.8b00143.
URL https://doi.org/10.1021/acs.langmuir.8b00143

[24] E. Staples, L. Thompson, I. Tucker, J. Penfold, R. Thomas, J. Lu, The influence of sorbitol on the adsorption of surfactants at the air–liquid interface, Journal of Colloid and Interface Science 184 (2) (1996) 391–398. doi:https://doi.org/10.1006/jcis.1996.0633.
URL https://www.sciencedirect.com/science/article/pii/S0021979796906335

[25] J. Li, C. Amador, M. R. Wilson, Computational predictions of interfacial tension, surface tension, and surfactant adsorption isotherms, Phys. Chem. Chem. Phys. 26 (2024) 12107–12120. doi:10.1039/D3CP06170A.
URL http://dx.doi.org/10.1039/D3CP06170A

[26] L. Shi, M. Ghezzi, G. Caminati, P. Lo Nostro, B. P. Grady, A. Striolo, Adsorption isotherms of aqueous c12e6 and cetyltrimethylammonium bromide surfactants on solid surfaces in the presence of low molecular weight coadsorbents, Langmuir 25 (10) (2009) 5536–5544, pMID: 19382783. arXiv:https://doi.org/10.1021/la8041988, doi:10.1021/la8041988.
URL https://doi.org/10.1021/la8041988

[27] J. R. Lu, Z. X. Li, R. K. Thomas, E. J. Staples, I. Tucker, J. Penfold, Neutron reflection from a layer of monododecyl hexaethylene glycol adsorbed at the air-liquid interface: the configuration of the ethylene glycol chain, The Journal of Physical Chemistry 97 (30) (1993) 8012–8020. arXiv:https://doi.org/10.1021/j100132a034, doi:10.1021/j100132a034.
URL https://doi.org/10.1021/j100132a034

[28] T. Sottmann, R. Strey, Ultralow interfacial tensions in water–n-alkane–surfactant systems, The Journal of Chemical Physics 106 (20) (1997) 8606–8615. arXiv:https://pubs.aip.org/aip/jcp/article-pdf/106/20/8606/19305487/8606_1_online.pdf, doi:10.1063/1.473916.
URL https://doi.org/10.1063/1.473916

[29] H. Cardenas, M. A. H. Kamrul-Bahrin, D. Seddon, J. Othman, J. T. Cabral, A. Mejia, S. Shahruddin, O. K. Matar, E. A. Muller, Determining interfacial tension and critical micelle concentrations of surfactants from atomistic molecular simulations, Journal of Colloid and Interface Science 674 (2024) 1071–1082. doi:https://doi.org/10.1016/j.jcis.2024.07.002.
URL https://www.sciencedirect.com/science/article/pii/S0021979724015066

[30] D. Balzer, Cloud point phenomena in the phase behavior of alkyl polyglucosides in water, Langmuir 9 (12) (1993) 3375–3384. arXiv:https://doi.org/10.1021/la00036a009, doi:10.1021/la00036a009.
URL https://doi.org/10.1021/la00036a009

[31] E.-M. Kutschmann, G. H. Findenegg, D. Nickel, W. von Rybinski, Interfacial tension of alkylglucosides in different apg/oil/water systems, Colloid and Polymer Science 273 (6) (1995) 565–571. doi:10.1007/BF00658686.
URL https://doi.org/10.1007/BF00658686

[32] M. K. Matsson, B. Kronberg, P. M. Claesson, Adsorption of alkyl polyglucosides on the solid/water interface: Equilibrium effects of alkyl chain length and head group polymerization, Langmuir 20 (10) (2004) 4051–4058, pMID: 15969397. arXiv:https://doi.org/10.1021/la035959p, doi:10.1021/la035959p.
URL https://doi.org/10.1021/la035959p

[33] C. J. Drummond, G. G. Warr, F. Grieser, B. W. Ninham, D. F. Evans, Surface properties and micellar interfacial microenvironment of n-dodecyl .beta.-d-maltoside, The Journal of Physical Chemistry 89 (10) (1985) 2103–2109. arXiv:https://doi.org/10.1021/j100256a060, doi:10.1021/j100256a060.
URL https://doi.org/10.1021/j100256a060

[34] B. B. Niraula, T. K. Chun, H. Othman, M. Misran, Dynamic-interfacial properties of dodecyl-
beta-d-maltoside and dodecyl-
beta-d-fructofuranosyl-
alpha-d-glucopyranoside at dodecane/water interface, Colloids and Surfaces A: Physicochemical and Engineering Aspects 248 (1) (2004) 157–166. doi:https://doi.org/10.1016/j.colsurfa.2004.08.073.
URL https://www.sciencedirect.com/science/article/pii/S0927775704005771

[35] Y. J. Nikas, S. Puvvada, D. Blankschtein, Surface tensions of aqueous nonionic surfactant mixtures, Langmuir 8 (11) (1992) 2680–2689. arXiv:https://doi.org/10.1021/la00047a018, doi:10.1021/la00047a018.
URL https://doi.org/10.1021/la00047a018

[36] S. Iglauer, Y. Wu, P. Shuler, Y. Tang, W. A. Goddard, Analysis of the influence of alkyl polyglycoside surfactant and cosolvent structure on interfacial tension in aqueous formulations versus n-octane, Tenside Surfactants Detergents 47 (2) (2010) 87–97 [cited 2024-12-30]. doi:doi:10.3139/113.110056.
URL https://doi.org/10.3139/113.110056

[37] A. Docoslis, R. Giese, C. van Oss, Influence of the water–air interface on the apparent surface tension of aqueous solutions of hydrophilic solutes, Colloids and Surfaces B: Biointerfaces 19 (2) (2000) 147–162. doi:https://doi.org/10.1016/S0927-7765(00)00137-5.
URL https://www.sciencedirect.com/science/article/pii/S0927776500001375

[38] M. Hoorfar, M. A. Kurz, Z. Policova, M. L. Hair, A. W. Neumann, Do polysaccharides such as dextran and their monomers really increase the surface tension of water?, Langmuir 22 (1) (2006) 52–56, pMID: 16378399. arXiv:https://doi.org/10.1021/la0512805, doi:10.1021/la0512805.
URL https://doi.org/10.1021/la0512805

[39] I. M. Zeron, J. L. F. Abascal, C. Vega, A force field of li+, na+, k+, mg2+, ca2+, cl, and so42 in aqueous solution based on the tip4p/2005 water model and scaled charges for the ions, The Journal of Chemical Physics 151 (13) (2019) 134504. arXiv:https://pubs.aip.org/aip/jcp/article-pdf/doi/10.1063/1.5121392/14052200/134504_1_online.pdf, doi:10.1063/1.5121392.
URL https://doi.org/10.1063/1.5121392

[40] PubChem, Calcium chloride, https://pubchem.ncbi.nlm.nih.gov/compound/Calcium-Chloride, accessed: 2025-2-19.

[41] PubChem, Sodium chloride, https://pubchem.ncbi.nlm.nih.gov/compound/Sodium-Chloride, accessed: 2025-2-19.

[42] D. Frenkel, A. J. C. Ladd, New Monte Carlo method to compute the free energy of arbitrary solids. Application to the fcc and hcp phases of hard spheres, The Journal of Chemical Physics 81 (7) (1984) 3188–3193. arXiv:https://pubs.aip.org/aip/jcp/article-pdf/81/7/3188/18950036/3188_1_online.pdf, doi:10.1063/1.448024.
URL https://doi.org/10.1063/1.448024
[43] T. C. Beutler, A. E. Mark, R. C. van Schaik, P. R. Gerber, W. F. van Gunsteren, Avoiding singularities and numerical instabilities in free energy calculations based on molecular simulations, Chemical Physics Letters 222 (6) (1994) 529–539. doi:https://doi.org/10.1016/0009-2614(94)00397-1.
URL https://www.sciencedirect.com/science/article/pii/0009261494003971
[44] J. Lidmar, Improving the efficiency of extended ensemble simulations: The accelerated weight histogram method, Phys. Rev. E 85 (2012) 056708. doi:10.1103/PhysRevE.85.056708.
URL https://link.aps.org/doi/10.1103/PhysRevE.85.056708
[45] V. Lindahl, J. Lidmar, B. Hess, Accelerated weight histogram method for exploring free energy landscapes, The Journal of Chemical Physics 141 (4) (2014) 044110. arXiv:https://pubs.aip.org/aip/jcp/article-pdf/doi/10.1063/1.4890371/13386596/044110$_{1o}nline.pdf, doi$: 10.1063/1.4890371.
URLhttps://doi.org/10.1063/1.4890371
[46] G. Torrie, J. Valleau, Nonphysical sampling distributions in monte carlo free-energy estimation: Umbrella sampling, Journal of Computational Physics 23 (2) (1977) 187–199. doi:https://doi.org/10.1016/0021-9991(77)90121-8.
URL https://www.sciencedirect.com/science/article/pii/0021999177901218
[47] V. Sresht, E. P. Lewandowski, D. Blankschtein, A. Jusufi, Combined molecular dynamics simulation–molecular-thermodynamic theory framework for predicting surface tensions, Langmuir 33 (33) (2017) 8319–8329, pMID: 28749139. arXiv:https://doi.org/10.1021/acs.langmuir.7b01073, doi:10.1021/acs.langmuir.7b01073.
URL https://doi.org/10.1021/acs.langmuir.7b01073
[48] H. Fan, D. E. Resasco, A. Striolo, Amphiphilic silica nanoparticles at the decanewater interface: Insights from atomistic simulations, Langmuir 27 (9) (2011) 5264–5274, pMID: 21449581. arXiv:https://doi.org/10.1021/la200428r, doi:10.1021/la200428r.
URL https://doi.org/10.1021/la200428r
[49] S. Kumar, J. M. Rosenberg, D. Bouzida, R. H. Swendsen, P. A. Kollman, The weighted histogram analysis method for free-energy calculations on biomolecules. i. the method, Journal of Computational Chemistry 13 (8) (1992) 1011–1021. arXiv:https://onlinelibrary.wiley.com/doi/pdf/10.1002/jcc.540130812, doi:https://doi.org/10.1002/jcc.540130812.
URL https://onlinelibrary.wiley.com/doi/abs/10.1002/jcc.540130812
[50] J. S. Hub, B. L. de Groot, D. van der Spoel, g_wham—a free weighted histogram analysis implementation including robust error and autocorrelation estimates, Journal of Chemical Theory and Computation 6 (12) (2010) 3713–3720. arXiv:https://doi.org/10.1021/ct100494z, doi:10.1021/ct100494z.
URL https://doi.org/10.1021/ct100494z
[51] H. Schott, Salting in of nonionic surfactants by complexation with inorganic salts, Journal of Colloid and Interface Science 43 (1) (1973) 150–155. doi:https://doi.org/10.1016/0021-9797(73)90358-5.
URL https://www.sciencedirect.com/science/article/pii/0021979773903585
[52] T. M. Doscher, G. E. Myers, D. C. Atkins, The behavior of nonionic surface active agents in salt solutions, Journal of Colloid Science 6 (3) (1951) 223–235. doi:https://doi.org/10.1016/0095-8522(51)90041-4.
URL https://www.sciencedirect.com/science/article/pii/0095852251900414
[53] C. Maltesh, P. Somasundaran, Effect of binding of cations to polyethylene glycol on its interactions with sodium dodecyl sulfate, Langmuir 8 (8) (1992) 1926–1930. arXiv:https://doi.org/10.1021/la00044a008, doi:10.1021/la00044a008.
URL https://doi.org/10.1021/la00044a008
[54] Y. Fang, M. Giesecke, I. Furo, Complexing cations by poly(ethylene oxide): Binding site and binding mode, The Journal of Physical Chemistry B 121 (9) (2017) 2179–2188, pMID: 28198620. arXiv:https://doi.org/10.1021/acs.jpcb.6b12381, doi:10.1021/acs.jpcb.6b12381.
URL https://doi.org/10.1021/acs.jpcb.6b12381
[55] M. Giesecke, F. Hallberg, Y. Fang, P. Stilbs, I. Furo, Binding of monovalent and multivalent metal cations to polyethylene oxide in methanol probed by electrophoretic and diffusion nmr, The Journal of Physical Chemistry B 120 (39) (2016) 10358–10366, pMID: 27622602. arXiv:https://doi.org/10.1021/acs.jpcb.6b08923, doi:10.1021/acs.jpcb.6b08923.
URL https://doi.org/10.1021/acs.jpcb.6b08923
[56] J. Penfold, E. Staples, I. Tucker, L. Thompson, R. Thomas, Adsorption of nonionic mixtures at the air–water interface: Effects of temperature and electrolyte, Journal of Colloid and Interface Science 247 (2) (2002) 404–411. doi:https://doi.org/10.1006/jcis.2001.8042.
URL https://www.sciencedirect.com/science/article/pii/S0021979701980427
[57] B. Uhlig, A. Wissemeier, Reduction of non-ionic surfactant phytotoxicity by divalent cations, Crop Protection 19 (1) (2000) 13–19. doi:https://doi.org/10.1016/S0261-2194(99)00076-9.
URL https://www.sciencedirect.com/science/article/pii/S0261219499000769
[58] S. Chandrasekhar, Stochastic problems in physics and astronomy, Rev. Mod. Phys. 15 (1943) 1–89. doi:10.1103/RevModPhys.15.1.
URL https://link.aps.org/doi/10.1103/RevModPhys.15.1
[59] P. Baur, J. Aponte, Co-penetration of Actives and Adjuvants and Its Significance for the Matched Pair Liaison, Vol. 1171 of ACS Symposium Series, American Chemical Society, 2014, pp. 23–39, 0. doi:10.1021/bk-2014-1171.ch002.
URL https://doi.org/10.1021/bk-2014-1171.ch002
[60] P. Baur, J. Schonherr, B. T. Grayson, Polydisperse ethoxylated fatty alcohol surfactants as accelerators of cuticular penetration. 2: Separation of effects on driving force and mobility and reversibility of surfactant action, Pesticide Science 55 (8) (1999) 831–842. arXiv:https://onlinelibrary.wiley.com/doi/pdf/10.1002/%28SICI%291096-9063%28199908%2955%3A8%3C831%3A%3AAID-PS25%3E3.0.CO%3B2-5, doi:https://doi.org/10.1002/(SICI)1096-9063(199908)55:8¡831::AID-PS25¿3.0.CO;2-5.
URL https://onlinelibrary.wiley.com/doi/abs/10.1002/%28SICI%291096-9063%28199908%2955%3A8%3C831%3A%3AAID-PS25%3E3.0.CO%3B2-5

[61] P. Baur, Surfactant effects on cuticular penetration of neutral polar compounds: Dependence on humidity and temperature, Journal of Agricultural and Food Chemistry 47 (2) (1999) 753–761, pMID: 10563965. arXiv:https://doi.org/10.1021/jf980507h, doi:10.1021/jf980507h.
URL https://doi.org/10.1021/jf980507h
[62] Y. Yamada, S. H. Wittwer, M. J. Bukovac, Penetration of Ions through Isolated Cuticles 123, Plant Physiology 39 (1) (1964) 28–32. arXiv:https://academic.oup.com/plphys/article-pdf/39/1/28/35668357/plphys_v39_1_28.pdf, doi:10.1104/pp.39.1.28.
URL https://doi.org/10.1104/pp.39.1.28
[63] Y. Yamada, H. P. Rasmussen, M. J. Bukovac, S. H. Wittwer, Binding sites for inorganic ions and urea on isolated cuticular membrane surfaces, American Journal of Botany 53 (2) (1966) 170–172. arXiv:https://bsapubs.onlinelibrary.wiley.com/doi/pdf/10.1002/j.1537-2197.1966.tb07317.x, doi:https://doi.org/10.1002/j.1537-2197.1966.tb07317.x.
URL https://bsapubs.onlinelibrary.wiley.com/doi/abs/10.1002/j.1537-2197.1966.tb07317.x
